# Cx43 levels guide apicobasal polarity in regenerating airway epithelial cells

**DOI:** 10.1038/s42003-026-10413-7

**Published:** 2026-06-12

**Authors:** Mehdi Badaoui, Alexandre Luscher, Mengjie Ma, Marc Bacchetta, Christian van Delden, Thilo Köhler, Arantxa Tabernero, Marc Chanson

**Affiliations:** 1 Department of Cell Physiology & Metabolism, Faculty of Medicine, University of Geneva, Geneva, Switzerland; 2 Department of Microbiology & Molecular Medicine, Faculty of Medicine, University of Geneva, Geneva, Switzerland; 3 Neuroscience Institute of Castile-Leon (INCYL), iBRAINS-IN-CyL, Department of Biochemistry and Molecular Biology, Biomedical Research Institute of Salamanca (IBSAL), University of Salamanca, Salamanca, Spain; 4 Department of Pediatrics, Gynecology and Obstetrics, Faculty of Medicine, University of Geneva, Geneva, Switzerland; 5 Geneva Center for Inflammation Research (GCIR), Faculty of Medicine, University of Geneva, Geneva, Switzerland

**Keywords:** Apicobasal polarity, Mechanisms of disease

## Abstract

Apicobasal polarity plays key roles in airway epithelial maturation and regeneration. Once polarized, the airway epithelium functions as a barrier against opportunistic pathogens. Yet, the mechanisms that temporally regulate polarization, a process which is disrupted in cystic fibrosis (CF), remain unclear. Here, we demonstrate the functional importance of connexin 43 (Cx43), a gap junction protein, which is normally repressed during regeneration of the airway epithelium. We show that prolonged post-transcriptional stabilization of Cx43 leads to altered collective cell orientation and compromised CF airway epithelial barrier. We report a bidirectional cooperation between Cx43 hemichannel function and adenosine signaling in disrupting ER-Golgi secretory axis, cytoskeleton dynamic and ectopic apical fibronectin production. Genetic and pharmacological inhibition of Cx43 channels re-established polarity and spatial organization of CF airway epithelial cells. Finally, targeting Cx43 hemichannels with mimetic peptides normalizes the CF-dependent fibronectin ectopic expression and prevents the enhanced *Pseudomonas aeruginosa* trapping to the CF epithelium. Our findings reveal a mechanism in which temporally controlled Cx43-mediated intercellular communication is crucial for coordinating intrinsic polarity programs and maintaining airway epithelium integrity.

## Abstract (web summary)

Mechanistic dissection of airway epithelium repair shows how aberrant Cx43 stabilization alters cell orientation, secretory axis structure, and fibronectin secretion, leading to barrier defects and increased *P. aeruginosa* adherence in cystic fibrosis.

## Introduction

Apicobasal polarity is a hallmark of epithelial cell organization that enables the functional compartmentalization required for maintaining tissue homeostasis^1^. It involves the segregation of the apical domain, which faces the epithelial lumen, from the basolateral domain, which interfaces with the extracellular matrix (ECM) component of the basement membrane. Polarity establishment and maintenance is orchestrated by a tightly regulated network involving intercellular junctions, cytoskeletal elements, and vesicular trafficking pathways. Among other functions, the actin cytoskeleton plays a critical role in forming tight junctions (TJs) delimiting the apical surface^2^, while microtubules are essential for directed vesicle transport and the spatial organization of organelles, such as the endoplasmic reticulum (ER) and Golgi apparatus^3^. The dynamic regulation of the cytoskeleton is orchestrated by small-GTPases, whose activity is tightly controlled by guanine nucleotide exchange factors (GEFs) and GTPase-activating proteins (GAPs). This regulation supports the integrity of the ER-Golgi intermediate compartment and facilitates vesicle movement along cytoskeletal tracks^4–6^. The ER-Golgi network thus serves as a central hub for the polarized trafficking of proteins.

In tightly organized epithelial tissues, directed protein secretion regulates cell-to-cell and cell-to-ECM communications to coordinate collective cell orientation. At the basal membrane, β1 integrin clustering promotes stable mechanical adhesion to the ECM^7^. The local concentration of fibronectin, a key ECM component that binds β1 integrin, acts as an adhesion-dependent stop signal that contributes to cell polarization^8^. TJs and adherens junctions (AJs), located at the lateral surfaces, provide mechanical linkage between adjacent cells, ensuring cohesive tissue architecture^7^. Synchronization of cellular responses to this multilayered interaction requires finely tuned coupling mechanisms between epithelial cells^9^. Connexin 43 (Cx43), a major component of gap junctions (GJs), plays a key role in cell coupling by enabling the rapid exchange of small molecules, ions, and second messengers between adjacent cells^10^. Connexin 43 is synthesized in the ER as a monomeric transmembrane protein and is post-translationally assembled into hexamers, known as connexons, in the trans-Golgi network^10–12^. These connexons are then transported in vesicles along microtubule tracks to the plasma membrane, where they form hemichannels (HCs). Hemichannels permit the release of adenosine triphosphate (ATP) into the extracellular environment, where it can influence neighboring cells via activation of P1 and P2 purine receptors^13^, while the docking of connexons from neighboring cells results in the formation of GJ channels^10^. The trafficking and assembly of Cx43 are tightly regulated by small-GTPases^12,14^, which coordinate its movement through the secretory pathway, influencing the formation of both HCs and GJs. Beyond its role in direct and paracrine intercellular communication, the non-channel function of Cx43 also contributes to the regulation of microtubule dynamics, organelle positioning, and establishment of cell polarity^15–18^.

In the airway epithelium, proper apicobasal polarity underlies critical functions, such as mucociliary clearance, epithelial integrity and barrier defense against inhaled pathogens. We and others have recently demonstrated that this polarity is partially disrupted in cystic fibrosis (CF), enhancing susceptibility to *Pseudomonas aeruginosa* (*Pa*) infections^19,20^. Defect of the cystic fibrosis transmembrane conductance regulator (CFTR) channel is associated with increased expression of the GEF Vav3, which coincides with mechanical TJs and AJs destabilization^19,21,22^. This, in turn, causes aberrant accumulation of fibronectin at the apical surface and ectopic β1 integrin localization on the luminal pole of the CF airway epithelium, promoting *Pa* adhesion^19,20,23,24^. In addition to the widespread defects in epithelial polarity, CFTR dysfunction may also impair the proper assembly, gating and localization of GJs. Irregular Cx43 trafficking and disorganized patterns have been reported in F508del-CFTR expressing human airway epithelial cells^25–28^, the commonest CF-causing mutation affecting CFTR trafficking^29^. While significant progress has been made in understanding the consequences of CFTR dysfunction, the underlying mechanisms linking CFTR defect and intercellular communication to these polarity defects remain underexplored. This gap in knowledge limits our ability to understand fully how cell–cell interactions and epithelial orientation are altered in CF, and how these changes may exacerbate the pathogenesis of CF and hinder effective tissue regeneration.

To bridge this gap, we employed a combination of primary cultures of human airway epithelial cells and CRISPR-edited Calu-3 cells, a human airway epithelial cell line to uncover persistent Cx43 expression during regeneration and polarity establishment of the CF airway epithelium. We report that Cx43 dysregulation affected the microtubule organization and the correct positioning of the ER-Golgi axis, which consequently perturbed the polarized fibronectin accumulation and bacterial adhesion. In addition, we show that this phenotype was sustained by a reinforcing feedback loop between Cx43 expression, activity, and CD73-fueled adenosine signaling. Finally, targeting Cx43 with connexins mimetic peptides in CRISPR-edited CFTR knockdown and F508del-CFTR primary airway epithelial cells restored the typical fibronectin asymmetry, epithelial integrity and efficiently reduced *Pa* adhesion to the cell surface. Thus, this work decodes polarity establishment in CF and provides an effective anti-adhesive intervention against respiratory infections.

## Results

### Persistent Cx43 expression in polarizing CF HAECs

**Fig. 1:**
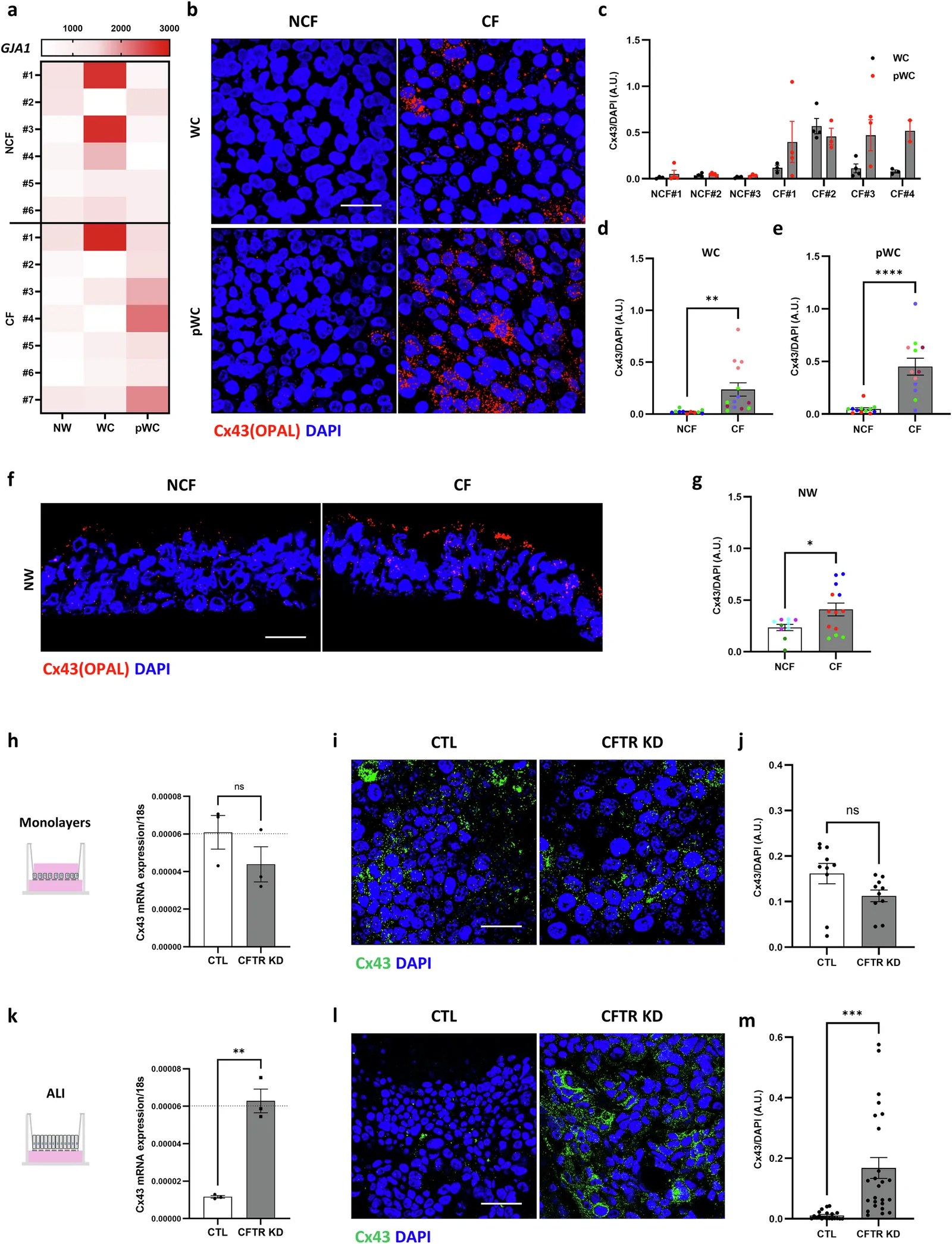
Persistent Cx43 expression in CF HAECs undergoing polarization. **a** Heatmap showing levels of *GJA1* gene expression in repairing NCF and CF primary HAECs in the non-wounded (NW) condition, at wound-closure (WC) and 48 h post-wound-closure (pWC). **b** Representative confocal images of Cx43 OPAL™ immunostaining (red) on NCF and CF primary HAECs at WC (upper panels) and pWC (bottom panels). Nuclei were stained with DAPI (blue). Scale bar: 20 μm. **c** Quantitative analysis of Cx43 immunofluorescence at WC and pWC for each donor. Pooled quantification of Cx43 expression per genotype at WC (**d**, 3 NCF donors, 4 CF donors, *n* = 11–14, **p = 0.0071), and pWC (**e**, 3 NCF donors, 4 CF donors, *n* = 12, ****p < 0.0001). The number of images acquired per donor varied depending on the number of Transwells® inserts that were successfully wounded and processed. *n* represents the total number of images analyzed per condition across all donors. Each donor is represented by a distinct color. **f** Representative confocal images of Cx43 OPAL™ immunostaining (red) of NW primary HAEC cultures from NCF and CF cryosections. Nuclei were stained with DAPI (blue). Scale bar: 20 μm. **g** Pooled quantification of Cx43 expression per genotype in NW primary HAECs (3 NCF donors, 3 CF donors, *n* = 10–13, *p = 0.0326). Each donor is represented by a distinct color, and *n* represents the total number of analyzed cryosections per condition across all donors. **h** Schematic of Calu-3 cells forming a monolayer, and RT-qPCR analysis of Cx43 mRNA expression in CTL and CFTR KD cells (3 biological replicates, not significant (ns) *p* = 0.2573). **i** Representative single focal plane images showing Cx43 immunostaining (green) in non-polarized CTL and CFTR KD cells. Nuclei were stained with DAPI (blue). Scale bar: 30 μm. **j** Quantitative analysis of Cx43 immunofluorescence in non-polarized CTL and CFTR KD cells (3 biological replicates, *n* = 10, not significant (ns) *p* = 0.0744). **k** Schematic of polarized Calu-3 cells grown at air-liquid interface (ALI), and RT-qPCR analysis of Cx43 mRNA expression in CTL and CFTR KD cells (3 biological replicates, **p = 0.0013). **l** Representative single focal plane images showing Cx43 immunostaining (green) in polarized CTL and CFTR KD cells. Nuclei were stained with DAPI (blue). Unstained regions correspond to areas where nuclei/cells are positioned outside the focal plane. Scale bar: 30 μm. **m** Quantitative analysis of Cx43 immunofluorescence in polarized CTL and CFTR KD cells (3 biological replicates, *n* = 20-25, ***p = 0.0002). *n* represents the total number of analyzed images per condition (**j**, **m**). Data are presented as mean ± s.e.m.; two-tailed unpaired Student’s *t* test (**d**, **e**, **g**, **h**, **j**, **k**, **m**).

Differentiated primary cultures of human airway epithelial cells (HAECs) at air-liquid interface (ALI) have the potential to re-establish their apicobasal polarity during the regeneration process. From a previous comparative transcriptomic profiling of regenerating non-CF (NCF) and F508del-CFTR (CF) primary HAECs^30^, we identified the top-expressed connexins’ isoforms as *GJA1*, *GJB2*, *GJB3,* and *GJB6*, encoding respectively Cx43, Cx26, Cx31, and Cx30 (Supplementary Fig. 1a). In response to mechanical wounding, both genotypes showed an overall transient increase in their mRNA levels during wound closure (WC), except for *GJA1*, which remained elevated in CF cultures at 48 h post-WC (pWC) (Fig. 1a and Supplementary Fig. 1a). To examine the prolonged *GJA1* mRNA expression at the protein level, we performed Cx43 immunostaining on repairing NCF and CF HAECs using the OPAL™ Tyramide Signal Amplification method. At WC and pWC, images were acquired in areas that had been previously damaged and subsequently repopulated by epithelial cells undergoing repair. While protein translation of the *GJA1* gene was barely detectable in NCF cultures, marked Cx43 expression was found in CF HAECs at both WC and pWC (Fig. 1b). Quantification of immunostainings at WC and pWC in 3 NCF and 4 CF cultures (Fig. 1c) revealed that Cx43 is abnormally upregulated in repairing CF primary HAECs (Fig. 1d, e, pooled values per genotype). Supplementary Fig. 1b shows co-staining of Cx43 with phalloidin at pWC, providing spatial context for Cx43 distribution within the regenerating CF airway epithelium. Representative confocal z-stack images highlight Cx43 localization at both the cell membrane and in the cytoplasm, depending on the basal, middle, or apical focal plane. This increased Cx43 expression was maintained in well-differentiated, non-wounded (NW), CF primary HAEC cultures (Fig. 1f, g). Immunostainings performed on cryosections revealed that Cx43 is detected throughout the differentiated CF airway epithelium, also at the apical membrane (Fig. 1f).

To confirm the causal relationship between CFTR dysfunction and temporal Cx43 expression defect, we compared Calu-3 cells knockdown for CFTR (CFTR KD) with their control (CTL) counterparts^31^. Cells were grown on Transwell® inserts until they formed a tight monolayer and then switched at ALI to drive the maturation of Calu-3 monolayers into a polarized airway epithelial model that recapitulates key features of the native epithelial barrier, including apicobasal polarity and tight junction–dependent integrity^21–23^. In this system, epithelial polarity and barrier function in CFTR KD cells persist beyond early maturation stages, remaining detectable up to 21 days post-ALI^21,22^. Day 14 (D14) post-ALI was selected to assess epithelial polarity, as this time point is widely used as a mature endpoint where proliferation is nil in both CTL and CFTR KD cells^23^. In monolayers, CTL and CFTR KD cells similarly expressed Cx43 at the mRNA (Fig. 1h) and protein levels (Fig. 1i, j). Again, polarization at ALI was associated with a strong repression of Cx43 expression in CTL cells, a process that was not observed in CFTR KD cultures, both at mRNA (Fig. 1k) and protein levels (Fig. 1l, m). Orthogonal projections of z-stack images acquired through the full height of the CFTR KD epithelium also revealed that some Cx43 is detected at the apical membrane (Supplementary Fig. 1c). These results indicate that Cx43 expression abnormally persists during polarization of CF HAECs.

### Post-transcriptional stabilization of Cx43 in CF HAECs leads to its close proximity with Vav3 in the ER

**Fig. 2:**
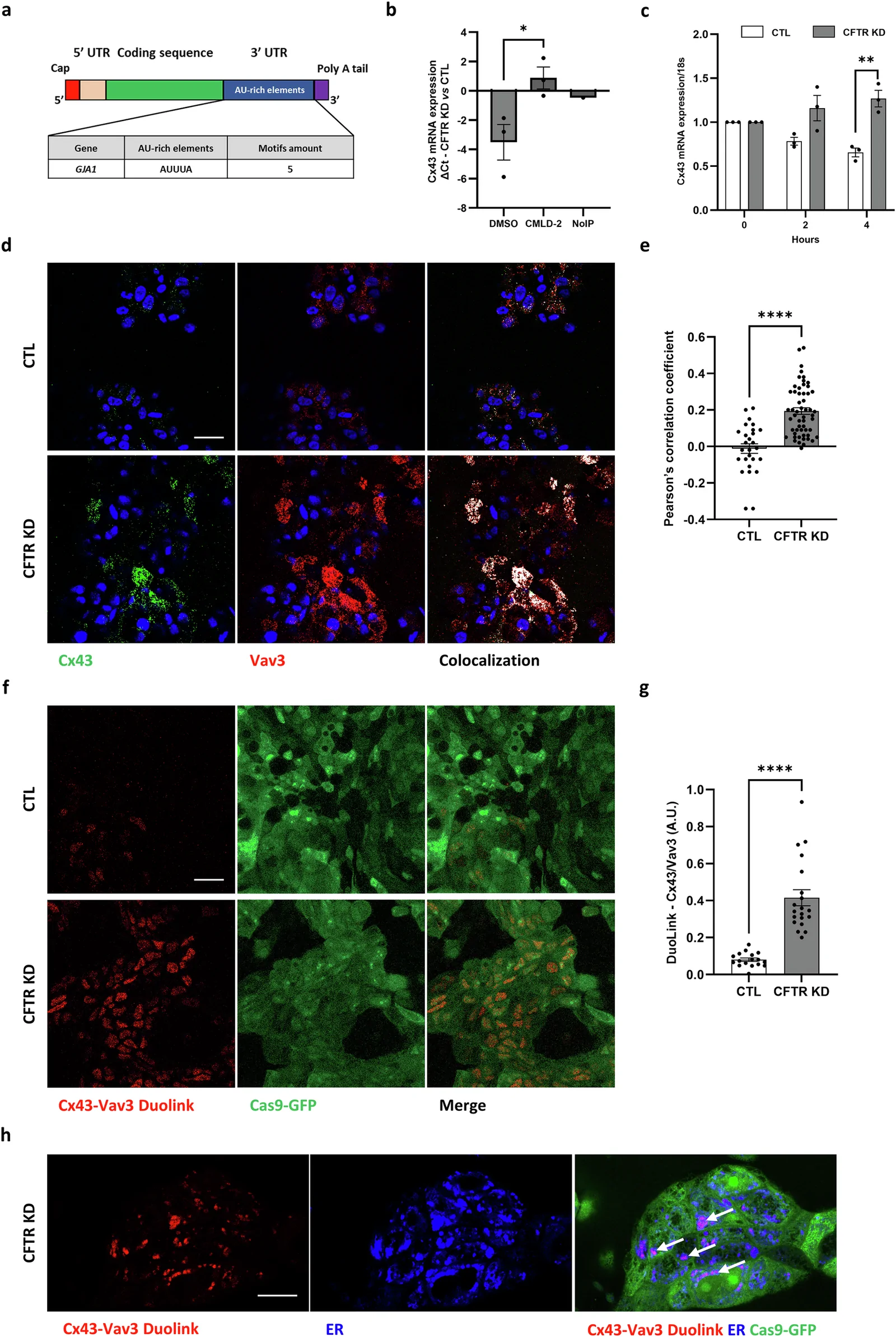
Cx43 mRNA stabilization in CFTR KD HAECs results in enhanced proximity with Vav3 in the ER. **a** Schematic of the motif and number of AU-rich elements (AREs) mapped in the 3′ untranslated region (UTR) of Cx43 mRNA. **b** RT-qPCR of Cx43 mRNA expression in the total mRNA extracted after HuR ribonucleoprotein immunoprecipitation in polarized CTL and CFTR KD Calu-3 cells treated or not with CMLD-2. The NoIP condition, where HuR antibody was omitted, served as a negative control. Values are represented as mean ΔCt (Ct_CFTR KD_ − Ct_CTL_) (3 biological replicates, *p = 0.0369). **c** Cx43 mRNA decay analyzed by RT-qPCR in polarized CTL and CFTR KD cells after transcription inhibition by actinomycin-D (3 biological replicates, **p = 0.0014). **d** Representative single focal plane images of Cx43 (green) and Vav3 (red) immunostaining in polarized CTL and CFTR KD cells, and their colocalization (white) using ZEN software. Nuclei were stained with DAPI (blue). Scale bar: 20 μm. **e** Colocalization between the green (Cx43) and red (Vav3) fluorescent signals was quantified using Pearson’s correlation coefficient (3 biological replicates, *n* = 27–55, ****p < 0.0001). **f** Detection of Cx43 and Vav3 proximity in polarized CTL and CFTR KD cells using Duolink® proximity ligation assay (PLA). Red dots show ligation events in single focal plane images, indicating that Cx43 and Vav3 were in close proximity (~40 nm). GFP-Cas9 (green) was used to visualize the cytoplasm. Scale bar: 20 μm. **g** Quantification of Cx43/Vav3 PLA signal (3 biological replicates, *n* = 17–20, ****p < 0.0001). **h** Localization of Cx43/Vav3 proximity was monitored by PLA with ER labeling using ER-tracker blue-white DPX dye (blue). GFP-Cas9 (green) allowed visualizing the cytoplasm. The merged single focal plane image shows colocalization of the red dots with the ER marker (white arrows), indicating that the interaction occurs in the ER compartment. Scale bar: 20 μm. *n* represents the total number of analyzed images per condition (**e**, **g**). Data are presented as mean ± s.e.m.; two-tailed unpaired Student’s *t* test (**b**, **e**, **g**); Two-way ANOVA with post hoc Tukey’s multiple comparison test (**c**).

We previously reported that primary CF HAECs and CFTR KD Calu-3 cells exhibit disrupted apicobasal polarity, where overexpression of the GEF Vav3 contributes to the ectopic relocalization of fibronectin and its receptor β1 integrin on the apical surface^19^. Vav3 expression was induced by HuR overexpression^24^, a post-transcriptional regulator that stabilizes mRNAs containing AU-rich elements (AREs) in the 3′-untranslated regions (UTRs)^24^. The 3′UTR of Cx43 mRNA exhibits five AUUUA motifs, which belong to the AREs core sequence (Fig. 2a). We then performed ribonucleoprotein immunoprecipitation experiments to isolate mRNAs specifically bound to HuR in CTL and CFTR KD cells, as previously reported^24^. Cx43 transcripts were detected in total mRNA extracted after HuR pull-down, with significant enrichment in CFTR KD cells (Fig. 2b). This increased interaction was lost upon treatment with CMLD-2, an inhibitor that competitively binds to HuR and disrupts its interaction with AREs, or when HuR antibody was omitted (NoIP). Next, we examined whether Cx43 persistent expression in CFTR KD cells is due to post-transcriptional stabilization by HuR. mRNA decay was monitored in CTL and CFTR KD cells at ALI D14 using actinomycin-D chase experiments. In CTL cells, almost 50% of Cx43 mRNA was degraded after 4 h of actinomycin-D treatment, whereas it remained stable over the same period in CFTR KD cells (Fig. 2c). These results indicate that increased interaction between Cx43 mRNA and HuR leads to post-transcriptional stabilization and persistence of Cx43 expression.

The exit of newly formed connexins from the ER to the Golgi, where they oligomerize before their transport to the plasma membrane are regulated by small-GTPases^12,14^. As GEF regulates small-GTPases activity, we investigated whether Vav3 and Cx43 intersect. Co-immunofluorescence experiments on CFTR KD cells at ALI showed increased colocalization between Vav3 and Cx43 (Fig. 2d), as evidenced by a significant increase in the Pearson’s correlation coefficient (Fig. 2e). Complementary Duolink® proximity ligation assay (PLA), which provides higher spatial resolution, indicated that the enhanced Vav3/Cx43 proximity occurs predominantly in the cytoplasmic compartment (Fig. 2f, g). In addition, PLA experiments in the presence of an ER marker show that Vav3 and Cx43 closeness is highly restricted to the ER (Fig. 2h). As shown in Supplementary Fig. 2a, z-stack confocal imaging on CFTR KD cells confirmed Cx43 detection not only at areas of cell-cell contacts (yellow arrows), but also in intracellular compartments (red arrows). We further examined the Vav3 and Cx43 relationship by co-immunostaining following OPAL™ tyramide signal amplification of Cx43 in cryosections of fully differentiated primary HAECs (Supplementary Fig. 2b). Quantitative analysis using Pearson’s correlation coefficient revealed increased colocalization in CF primary cultures (Supplementary Fig. 2c), consistent with findings in CFTR KD cells. These results indicate that Vav3 and Cx43 are in close proximity in the ER of CF HAECs.

### Cx43 expression disrupts polarization of the ER-Golgi axis in CF HAECs

**Fig. 3:**
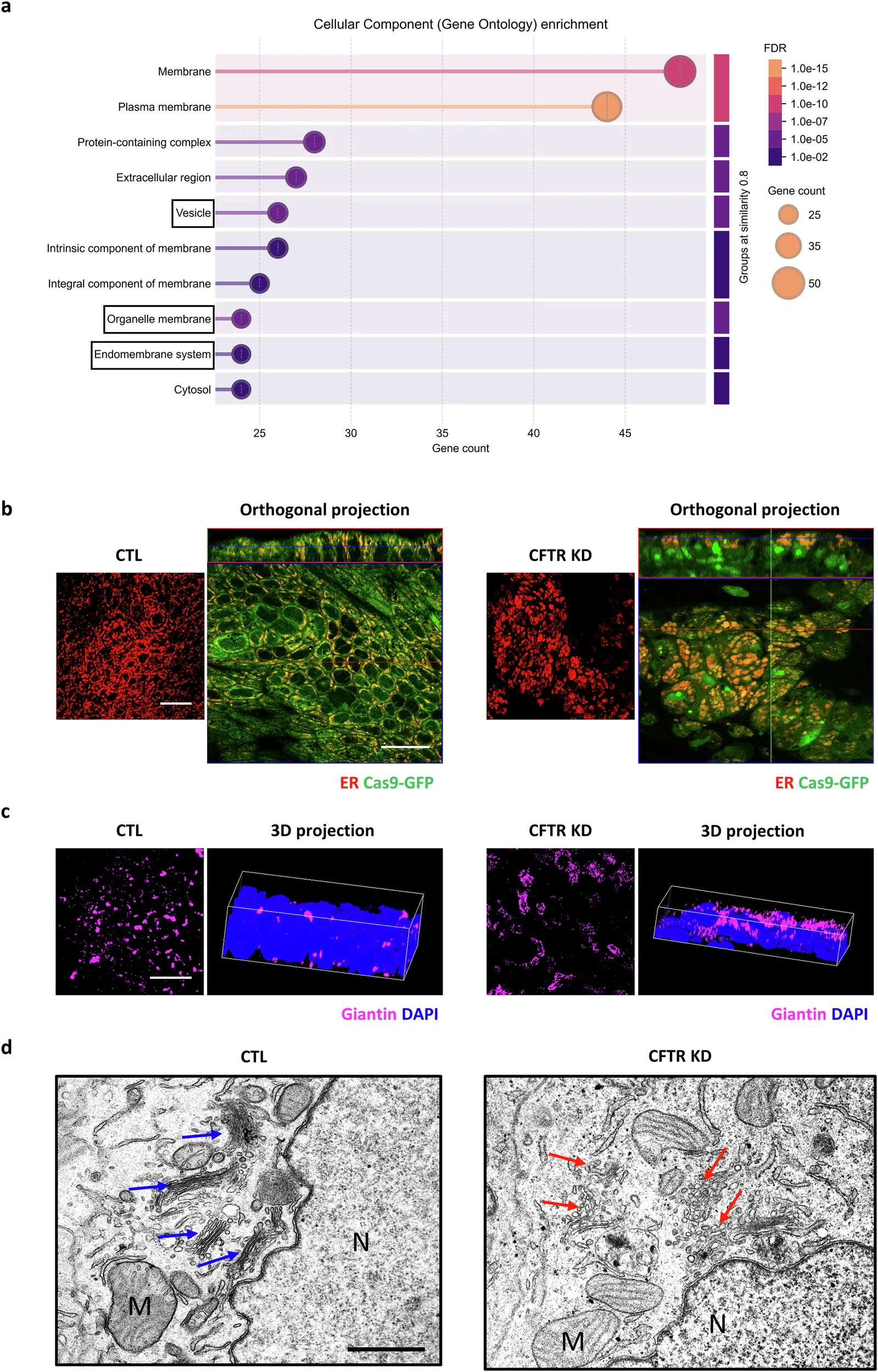
Cx43 expression disorganizes the ER-Golgi axis in CFTR KD HAECs. **a** STRING analysis to determine over-represented Gene Ontology cellular component (CC) terms for pathways identified by gene set enrichment analysis. Statistical significance is indicated as FDR. **b** Representative top view of 3D reconstructed confocal images showing ER (red) labeling using ER-tracker blue-white DPX dye in polarized CTL and CFTR KD cells. GFP-Cas9 (green) allows visualization of the cytoplasm. For each condition, orthogonal views of z-stack confocal images are shown on the right panels, providing yz-plane side views of ER distribution across the cells. Scale bar: 20 μm. **c** Representative confocal images of Golgi immunostaining (magenta) in polarized CTL and CFTR KD cells using anti-giantin antibody. Nuclei were stained with DAPI (blue). For each condition, a 3D projection of z-stack confocal images focusing on the apical surface of the epithelium is shown, providing a comprehensive view of the localization and distribution of the Golgi around the nucleus. Scale bar: 20 μm. **d** Transmission electron microscopy reveals Golgi structure in polarized Calu-3 cells, with breakdown of the typical stacked cisternal organization (blue arrows) into smaller and dispersed elements in CFTR KD cells (red arrows). N: nucleus, M: mitochondria. Scale bar: 1 μm.

Directed protein transport and trafficking during polarity establishment rely on the proper orientation of the ER, Golgi, and vesicles’ axis. To gain insights into the secretory pathway, we analyzed the transcriptomic profiles of polarized CTL and CFTR KD Calu-3 cells. Gene set enrichment analysis revealed that “synaptic vesicle pathway/cycle” and “axon guidance” pathways were significantly dysregulated in CFTR KD cells (Supplementary Fig. 2d). Moreover, functional enrichment visualization using STRING database indicated that the set of genes significantly altered in these pathways are component of interconnected organelles represented by the “endomembrane system”, “organelle membrane”, “vesicles” and “plasma membrane” (Fig. 3a). Next, we imaged the morphology and the orientation of the ER/Golgi organelles in CTL and CFTR KD cells at ALI D14. Labeling the ER with the blue-white DPX tracker in CTL cells showed perinuclear organization of the ER network that extends toward the apical and basolateral surfaces of the cells, creating a structured pathway for cargo transport to the appropriate membrane (Fig. 3b). In contrast, CFTR KD cells displayed ER staining clustered in distinct subdomains and disorganization of the secretory axis, often detected toward the apical pole (Fig. 3b). Immunostaining for giantin also revealed higher Golgi dispersal in CFTR KD cells (Fig. 3c). We then performed transmission electron microscopy (TEM), which confirmed Golgi dispersal at the ultrastructural level. TEM images showed a breakdown of the typical stacked cisternal organization of the Golgi into smaller, dispersed units in CFTR KD cells (red arrows), consistent with the punctate and dispersed pattern observed by immunofluorescence (Fig. 3d). These results provide complementary evidence that the ER-Golgi axis is specifically disrupted in polarizing CFTR KD cells, which may affect protein asymmetric delivery to the basolateral and apical membranes.

The disrupted ER-Golgi axis in CFTR KD cells at ALI D14 coincides with persistent expression of Cx43. To determine if there is a causal relationship between Cx43 expression and secretory axis disorganization, we generated double knockdown Calu-3 cells for *CFTR* and *GJA1* (Cx43/CFTR KD cells) using CRISPR-Cas9 gene editing, which efficiently reduced Cx43 expression (Supplementary Fig. 3a, b). The efficiency of CFTR knockdown in Cx43/CFTR KD cells was determined by Ussing chamber measurements of CFTR-dependent transepithelial ion transport. As shown in Supplementary Fig. 3c, d, electrophysiology confirmed robust CFTR silencing and demonstrated that Cx43 depletion does not affect CFTR knockdown efficiency. We also evaluated for compensation of Cx43 knockdown by expression of other connexins known to be expressed in primary HAECs. As shown in Supplementary Fig. 3e, *GJB3* and *GJB6* transcripts were undetectable while *GJB2* was near the limit of detection without compensatory upregulation.

**Fig. 4:**
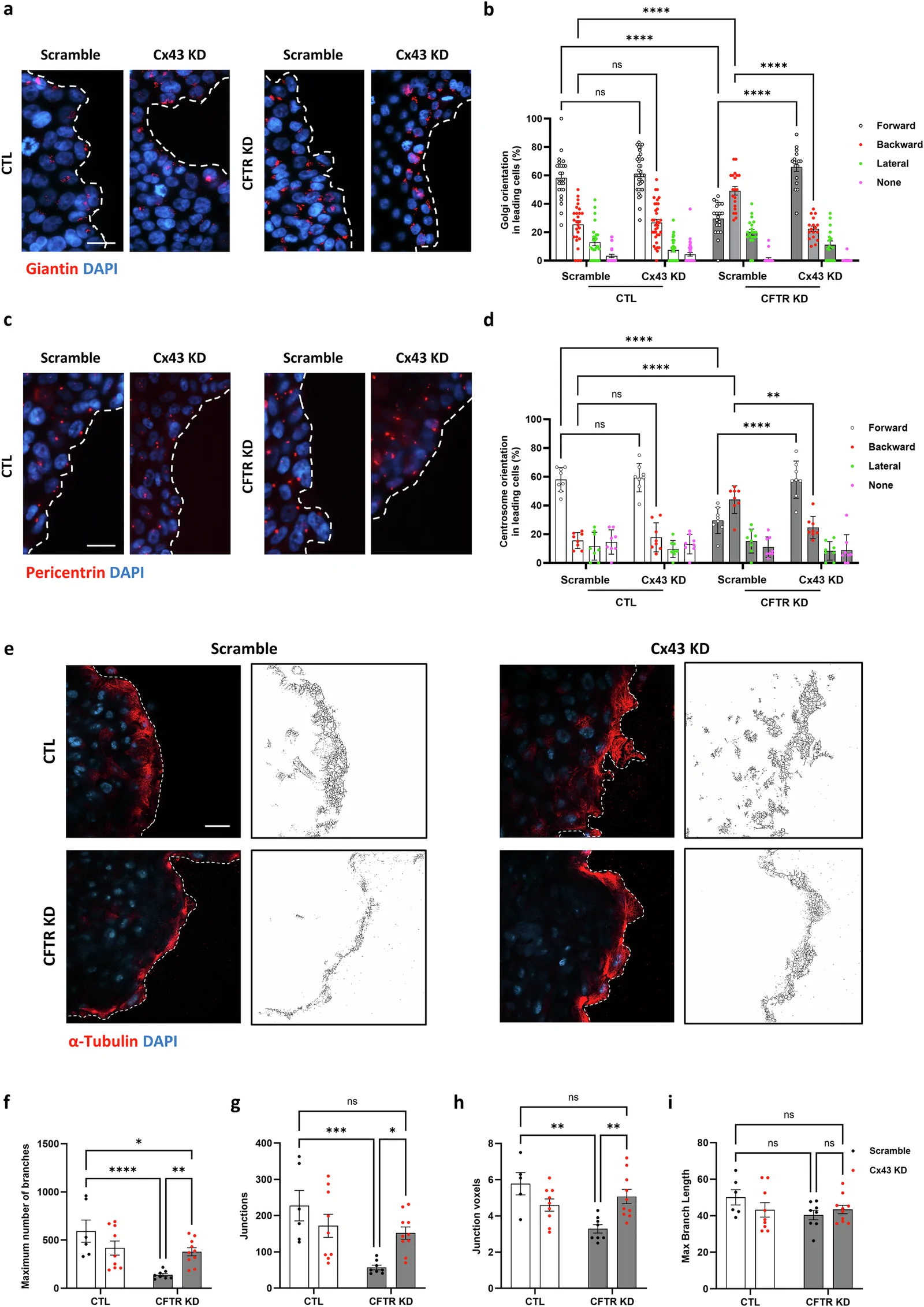
Cx43 expression disrupts Golgi-centrosome orientation and microtubule branching in repairing CFTR KD HAECs. **a**–**d** Representative images for giantin (Golgi marker, red (**a**)) and pericentrin (centrosome marker, red (**c**)) immunostainings performed in repairing CTL and CFTR KD Calu-3 cells grown on coverslips depleted or not for Cx43. The orientation of the Golgi and centrosome in leading edge cells was assessed 3 h post-scratch. Nuclei were stained with DAPI (blue). The dashed lines indicate the edge of the wounds. Scale bar: 20 μm. At the leading edge, cells were categorized based on the orientation of the Golgi (**b**) and centrosome (**d**): forward, backward, lateral, or none (no-orientation). The percentage of cells in each category was calculated relative to the total number of cells at the leading edge (**b** 3 biological replicates, *n* = 17–35, ****p < 0.0001; **d** 3 biological replicates, *n* = 9, **p = 0.0023, ****p < 0.0001). **e** Representative confocal images of α-tubulin immunostaining, performed during wound healing of CTL and CFTR KD grown on coverslips depleted or not for Cx43 (left panels for each condition, red). Skeletonization function using ImageJ was applied to reduce the microtubule network to a simplified, one-pixel-wide structure, enabling quantitative analysis (right panels for each condition). Microtubule networks in leading-edge cells are shown 3 h post-wound. Nuclei were stained with DAPI (blue). Scale bar: 20 μm. Quantification of the maximum number of branches (**f** the total count of points in the network where microtubules branch out from an existing structure, 3 biological replicates, *n* = 6–10, *p = 0.0293, **p = 0.0092, ****p < 0.0001), the number of junctions (**g** the count of points where multiple microtubule segments intersect or converge, 3 biological replicates, *n* = 6–10, *p = 0.0473, ***p = 0.0009), junction voxels (**h** where multiple microtubule branches meet, 3 biological replicates, *n* = 6–10, **p = 0.0019, **p = 0.0082), and the maximum branch length (**i** 3 biological replicates, *n* = 6–10, not significant (ns) *p* > 0.05). *n* represents the total number of analyzed images per condition (**b**, **d**, **f**–**i**). Data are shown as mean ± s.e.m.; two-way ANOVA with post hoc Tukey’s multiple comparison test (**b**, **d**, **f**–**i**).

Golgi repositioning typically occurs during directed cell migration, facilitating polarized trafficking and membrane delivery of newly synthesized proteins toward cells at the leading edge. This spatial reorganization, regulated by centrosome positioning, serves as a hallmark of cell polarity^32^. Thus, we used a wound healing assay to investigate early spatial reorganization in cells at the wound edge. Golgi and centrosome orientation in leading edge cells was assessed 3 h post-wounding by immunostaining for giantin and pericentrin in CTL and CFTR KD cells (Fig. 4a, c). Quantification was classified according to their positioning relative to the nuclei (forward, backward, lateral, or none when giantin staining overlapped with DAPI), as illustrated in Supplementary Fig. 4a. For each image, the percentage of cells displaying each orientation was calculated relative to the total number of cells at the leading edge. In CFTR KD cells, a lower percentage of leading cells showing forward Golgi and centrosome positioning was observed when compared to their control counterparts (Fig. 4b, d). They showed mostly backward orientation of the Golgi and centrosome, even 3 h post-wounding. In contrast, Cx43 depletion in CFTR KD cells restored the forward orientation of the leading cells to the same level as CTL cells (Fig. 4b, d). To ensure robustness and reproducibility of this wounding assay, Golgi and centrosome orientation were additionally quantified using automated segmentation and angle measurements relative to the wound edge (Supplementary Fig. 4b). The absolute Golgi angle measurement in leading edge cells indicates the magnitude of deviation between the nucleus-Golgi/centrosome axis and the nucleus-wound axis. This independent, automated analysis showed restoration of forward-oriented angles of Golgi and centrosome positioning upon Cx43 depletion in CFTR KD cells (Supplementary Fig. 4c, d).

The regulation of Golgi positioning and organization by the centrosome is microtubule-dependent^33^. Moreover, Golgi itself can regulate microtubule architecture^34^. Hence, we characterized microtubule organization by α-tubulin imaging in cells at the leading edge 3 h post-wounding. Skeletonization of the microtubules was achieved to resolve their branching properties in CFTR KD and Cx43/CFTR KD cells (Fig. 4e). The maximum number of branches, junctions and junctions with minimum two branches (junction voxels) were markedly reduced in CFTR KD cells as compared to CTL cells (Fig. 4f–h). Importantly, the branching capacity was restored after Cx43 knockdown (Fig. 4f–h), an effect that did not affect the maximum branch length (Fig. 4i). To evaluate whether these alterations were associated with differences in cell proliferation between CFTR KD and Cx43/CFTR KD cells, cell growth was determined during the proliferative phase (24, 48, and 72 h post-seeding). However, no significant differences were observed between all Calu-3 cell models at any time point (Supplementary Fig. 4e). Also, no differences in Cx43/CFTR KD wound closure rates when compared to CFTR KD cells were found (Supplementary Fig. 4f). Finally, Golgi/centrosome misalignment and reduced microtubule branching during wound repair (Supplementary Fig. 5a–h), as well as dispersed Golgi elements (Supplementary Fig. 6a, b), were recapitulated in one primary CF HAEC culture, supporting the relevance of our findings in CFTR KD Calu-3 cells. Collectively, these results indicate that Cx43 persistence disrupts microtubule organization and polarization of the Golgi/centrosome axis in CF HAECs, which is necessary for spatial cell orientation.

### Cx43 expression promotes ectopic fibronectin deposition on the apical surface of CFTR KD HAECs

**Fig. 5:**
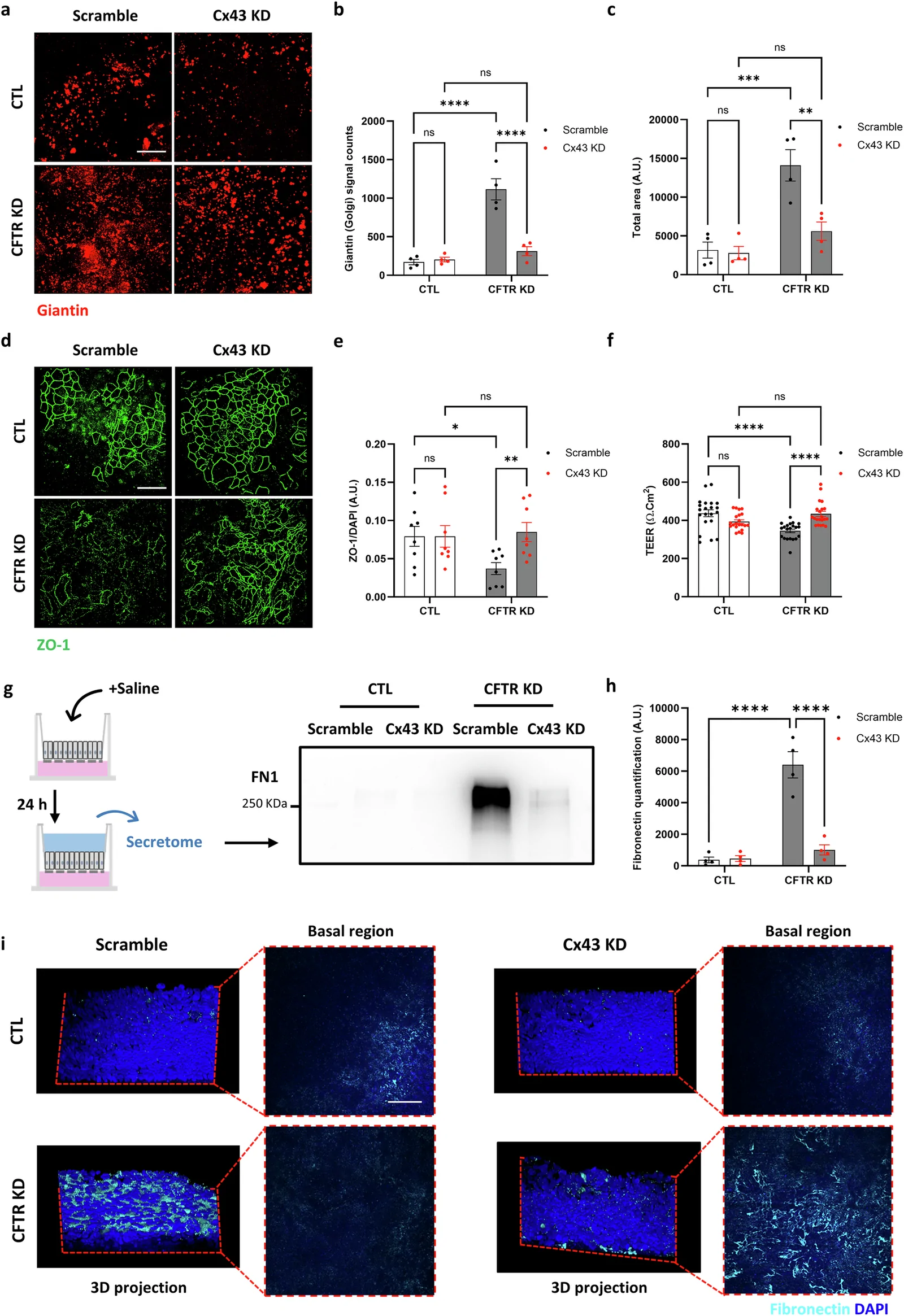
Cx43 depletion restores epithelial integrity and fibronectin asymmetry in polarized CFTR KD HAECs. **a** Representative top view of 3D reconstructed confocal images showing giantin immunostaining (red), performed in polarized CTL and CFTR KD Calu-3 cells depleted or not for Cx43. Scale bar: 20 μm. Quantification of Golgi dispersal (**b** 4 biological replicates, ****p < 0.0001) and the total area covered by the fluorescent signal (**c** 4 biological replicates, **p = 0.0065, ***p = 0.0003). **d** Representative top view of 3D reconstructed confocal images showing ZO-1 immunostaining (green), performed in polarized CTL and CFTR KD depleted or not for Cx43. Scale bar: 40 μm. **e** Quantification of the ZO-1 immunofluorescence (3 biological replicates, *n* = 8, *p = 0.0206, **p = 0.0094). *n* represents the total number of analyzed images per condition. **f** Transepithelial electrical resistance (TEER) was measured to assess the integrity and functional status of tight junctions in 3 biological replicates of polarized CTL and CFTR KD cells depleted or not for Cx43. TEER values are expressed as Ω.cm² (4 biological replicates, *n* = 21, ****p < 0.0001). *n* represents the total number of Transwell® inserts analyzed per condition. **g** Representative Western blot depicting fibronectin (FN1) detection in the apical secretome collected 24 h after incubation of saline on top of polarized CTL and CFTR KD cells, depleted or not for Cx43. Schematic in the left panel illustrates how the secretomes were collected. **h** Fibronectin band intensity was quantified for all collected secretomes (*n* = 4 biological replicates, ****p < 0.0001). **i** Representative confocal images of fibronectin (cyan) immunostaining in polarized CTL and CFTR KD Calu-3 cells depleted or not for Cx43. Nuclei were stained with DAPI (blue). For each condition, the left panels show the 3D projection of z-stack confocal images providing a comprehensive view of the apical deposition of fibronectin, and the images on the right depict a single focal plane image at the basal side of the epithelium. Scale bar: 50 μm. Data are presented as mean ± s.e.m.; two-way ANOVA with post hoc Tukey’s multiple comparison test (**b**, **c**, **e**, **f**, **h**).

Given the critical effect of Cx43 on spatial cell orientation, we next sought to investigate whether persistent Cx43 expression in CFTR KD Calu-3 cells drives the apicobasal polarity breaking. At ALI D14, we observed that the counts of dispersed Golgi signals and the total area immunostained by giantin, which were increased in CFTR KD cells, were reduced back to control levels by Cx43 depletion, indicating reduced Golgi scattering and dispersal (Fig. 5a–c). Next, we immunostained the tight junction protein ZO-1, a key marker of polarity establishment and used to distinguish the apical and basolateral domains. While ZO-1 assembly at the cortical side of the CFTR KD epithelia was altered, Cx43 depletion was associated with redistribution of ZO-1 at TJs (Fig. 5d, e). Orthogonal views of giantin and ZO-1 signals co-stained with DAPI are shown in Supplementary Fig. 7a, b, respectively. Restoration of epithelial integrity was confirmed in Cx43/CFTR KD cells, as indicated by increased transepithelial electrical resistance (TEER) to levels comparable to the CTL cells (Fig. 5f); reduction in TEER is a feature of CF HAECs, as shown in Supplementary Fig. 6c. Finally, we investigated whether Cx43 depletion affected apical secretion of fibronectin. To this end, the apical secretome of Calu-3 cells cultured at ALI was collected after incubation with saline for 24 h. Western blot experiments showed that detection of fibronectin was strongly reduced in secretomes of Cx43/CFTR KD cultures as compared to CFTR KD cells (Fig. 5g, h). Importantly, 3D projection of confocal z-stack images showed that fibronectin was now detected at the basal side of the Cx43/CFTR KD epithelium (Fig. 5i). These results strongly suggest that persistent Cx43 expression in CF HAECs causes the apicobasal polarity breaking responsible for abnormal production of fibronectin at the apical surface.

### Cx43 channel activity and adenosine signaling are functionally connected to sustain apical fibronectin deposition in CFTR KD HAECs

**Fig. 6:**
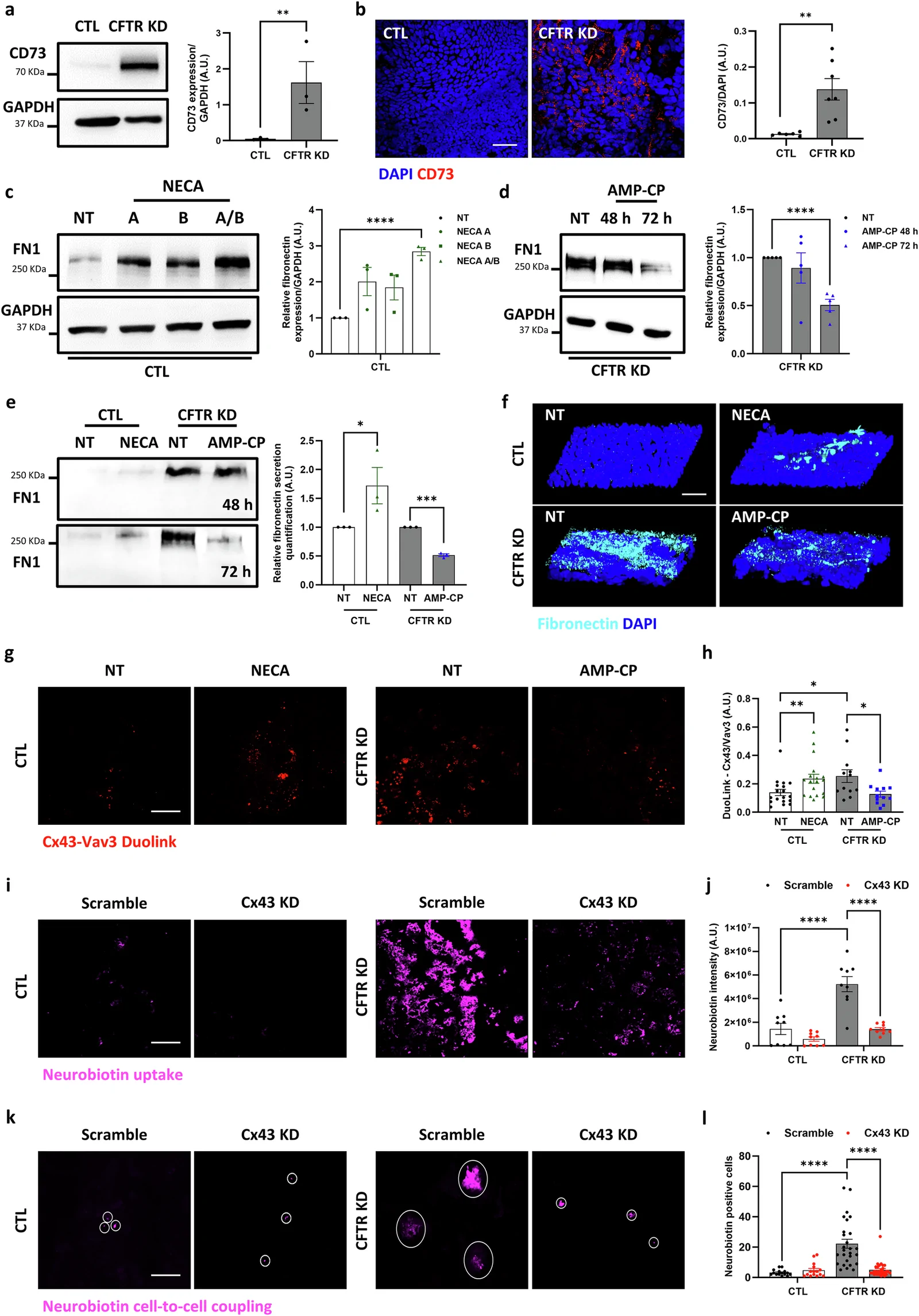
Cx43 channel function and adenosine signaling cooperate to maintain apical fibronectin production in CFTR KD HAECs. **a** Representative Western blot depicting CD73 expression in polarized CTL and CFTR KD Calu-3 cells (left panel) and quantification (right panel). GAPDH was used as a loading control. The bands were quantified by densitometry, and CD73 expression was normalized to GAPDH (3 biological replicates, **p = 0.0021). **b** Representative top view of 3D reconstructed confocal images showing CD73 immunostaining (red), performed in polarized CTL and CFTR KD cells. Nuclei were stained with DAPI (blue). Scale bar: 50 μm. CD73 immunofluorescence was quantified from 3 biological replicates. The intensity of the CD73 signal was normalized to DAPI staining to account for cell number (3 biological replicates, *n* = 6–7, **p = 0.0012). Representative Western blot depicting fibronectin (FN1) expression in polarized CTL (**c**) and CFTR KD cells (**d**), respectively, treated with NECA (apically (A), basally (B) or both (A/B)) for 72 h or AMP-CP for 48 and 72 h. GAPDH was used as a loading control. The bands were quantified by densitometry (right panel of each gel), and FN1 expression was normalized to GAPDH (CTL, 3 biological replicates, ****p < 0.0001; CFTR KD, 5 biological replicates, ****p < 0.0001). **e** Representative Western blot depicting FN1 detection in the apical secretome collected 24 h after incubation of saline on top of polarized CTL and CFTR KD cells, respectively, treated with NECA or AMP-CP for 48 h and 72 h. Fibronectin band intensity at 72 h was quantified (right panel) for all collected secretomes (3 biological replicates, *p = 0.0440, ***p = 0.0001). **f** Representative 3D projection of z-stack confocal images providing a comprehensive view of the apical deposition of fibronectin (cyan) in polarized CTL and CFTR KD cells, respectively treated with NECA or AMP-CP for 72 h. Nuclei were stained with DAPI (blue). Scale bar: 40 μm. **g** Detection of Cx43 and Vav3 proximity using Duolink® proximity ligation assay (PLA) in polarized CTL and CFTR KD cells, respectively treated with NECA or AMP-CP for 72 h. Red signal indicates ligation events when Cx43 and Vav3 are in close proximity (~40 nm). Scale bar: 20 μm. **h** Quantification of Cx43/Vav3 PLA signal (3 biological replicates, *n* = 12–18, **p = 0.0023 (CTL); *p = 0.0372 (CFTR KD)). **i** Confocal images showing neurobiotin dye uptake as a measure of hemichannel activity in polarized CTL and CFTR KD cells depleted or not for Cx43. Intracellular neurobiotin was labeled with streptavidin-Alexa Fluor 647 (magenta). Scale bar: 20 μm. **j** Quantification of intracellular neurobiotin fluorescence intensity from 3 biological replicates (3 biological replicates, *n* = 9, ****p < 0.0001). **k** Representative images showing neurobiotin cell-to-cell coupling in polarized CTL and CFTR KD cells, depleted or not for Cx43. Each circle indicates neurobiotin transfer to neighboring cells 3 min post-microinjection of a single cell. Neurobiotin-positive cells were labeled with streptavidin-Alexa Fluor 647 (magenta). Scale bar: 100 μm. **i** Quantification of neurobiotin positive cells (3 biological replicates, *n* = 15–30, ****p < 0.0001). *n* represents the total number of analyzed images (**b**, **h**, **j**) or quantified microinjections (**l**) per condition. Data are presented as mean ± s.e.m.; two-tailed unpaired Student’s *t* test (**a**–**e**, **h**); two-way ANOVA with post hoc Tukey’s multiple comparison test (**j**, **l**).

To elucidate the relationship between Cx43 expression and ectopic fibronectin secretion, we examined the effects of extracellular adenosine, which was previously reported to enhance luminal fibronectin secretion in human intestinal cells^35^. Adenosine is an endogenous signaling molecule that modulates physiological responses by activating P1 purine receptors. Adenosine is generated from the sequential enzymatic conversion of extracellular ATP (eATP) by the ecto-nucleotidases CD39 and CD73, which are membrane-bound enzymes^36^. Interestingly, Western blot (Fig. 6a) and immunofluorescence (Fig. 6b) experiments revealed an increased expression of CD73 in CFTR KD Calu-3 cells at ALI, indicating their higher predisposition to produce adenosine. Orthogonal views of CD73 are shown in Supplementary Fig. 7c. Next, we stimulated CTL cells with NECA (Fig. 6c), a potent agonist of P1 receptors^37^, and CFTR KD cells with AMP-CP (Fig. 6d), an inhibitor of CD73 activity^38^. As shown in Fig. 6c, fibronectin expression was strongly induced in CTL cells treated either apically (A), basally (B), or both (A/B) with NECA for 72 h. In contrast, fibronectin expression was markedly reduced in CFTR KD cells treated for 72 h with AMP-CP (Fig. 6d). Of note, control treatments of CTL and CFTR KD cells with AMP-CP or NECA, respectively, had no significant effects, which is expected regarding CD73 expression levels in these cells (Supplementary Fig. 8a, b). Fibronectin was also detected in the apical secretome (Fig. 6e) and at the surface (Fig. 6f) of CTL cells exposed to NECA for 72 h. Treatment of CFTR KD cells with AMP-CP, however, highly reduced apical detection of fibronectin (Fig. 6e, f) after 72 h of treatment. These results indicate that adenosine signaling is upregulated in CFTR KD cells and contributes to fibronectin apical production.

We have previously shown that extracellular adenosine stimulates the activity of Cx43 in Calu-3 cells^39^. This led us to hypothesize that released ATP through Cx43 HCs could be converted to adenosine, thus contributing to a feed-forward regulatory loop by activation of adenosine-dependent intracellular signaling. In agreement with this idea, Duolink® PLA showed that modulation of adenosine signaling with NECA in CTL cells or AMP-CP in CFTR KD cells correlated positively with Cx43/Vav3 closeness (Fig. 6g, h). As Vav3/Cx43 proximity might deliver more Cx43 HCs to the apical plasma membrane, we performed dye uptake experiments, a widely used assay to determine Cx43 HC activity (Supplementary Fig. 8c). Detection of neurobiotin with Alexa Fluor^TM^ streptavidin indicated increased uptake in CFTR KD cells (Fig. 6i, j) as compared to CTL cells. This increase was virtually abolished in Cx43/CFTR KD cells (Fig. 6i, j), indicating that the increased HC activity in CFTR KD cells at ALI is Cx43 specific. Furthermore, the adenosine-sustained delivery of Cx43 HCs to the plasma membrane may ultimately increase the formation of GJs. Gap junctional intercellular communication (GJIC) was then monitored by dye coupling through intracellular microinjection of neurobiotin (Supplementary Fig. 8d). Cx43-dependent increase in GJIC was confirmed in CFTR KD cells, as revealed by Alexa Fluor^TM^ streptavidin detection of neurobiotin that diffused from the injected cells to its neighbors (Fig. 6k, l). Again, modulation of adenosine signaling with NECA in CTL cells or AMP-CP in CFTR KD cells correlated with the extent of GJIC (Supplementary Fig. 8e), confirming the positive feedback loop between adenosine signaling, Cx43 expression and Cx43 channel formation in CFTR KD cells. Finally, we measured the amount of adenosine released in the apical secretome of CTL and CFTR KD cells. Adenosine level was strongly increased only in CFTR KD cells, an effect that was inhibited in the presence of the gap junction inhibitor 18α-glycyrrhetinic acid (αGA), indicating that it is dependent on Cx43 HC opening (Supplementary Fig. 8f). Collectively, these results indicate that persistent Cx43 expression and channel activity fuel the adenosine-dependent apical fibronectin production and deposition in CFTR KD cells.

### Targeting Cx43 reduces adenosine-dependent apical fibronectin production in CFTR KD HAECs

**Fig. 7:**
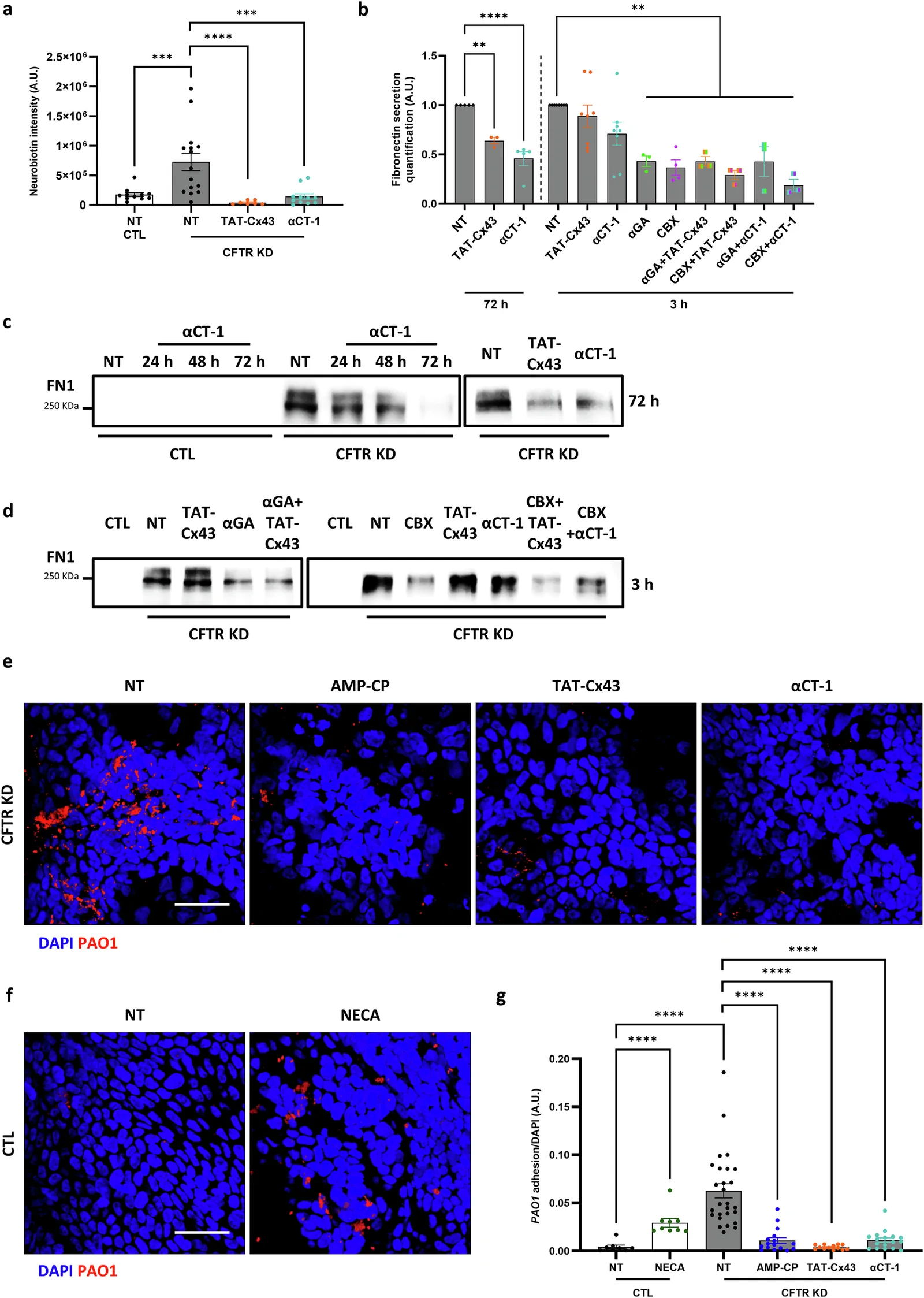
Cx43 inhibition by mimetic peptides reduces apical fibronectin detection and PAO1 adhesion to CFTR KD HAECs. **a** Quantification of intracellular neurobiotin dye uptake in polarized CTL and CFTR KD Calu-3 cells. CFTR KD cells were apically pre-treated with TAT-Cx43 or αCT-1 for 3 h before neurobiotin incubation. Three biological replicates, *n* = 7–15, ***p = 0.0006 (NT), ****p < 0.0001 (TAT-Cx43), ***p = 0.0004 (αCT-1). Quantifications (**b**) and representative Western blots showing fibronectin (FN1) detection in the apical secretomes collected 24 h (**c**) and 3 h (**d**) after incubation of saline on top of polarized cells. The left blot in (**c**) depicts polarized CTL and CFTR KD cells treated with αCT-1 for 24, 48, and 72 h, while the right blot in (**c**) displays CFTR KD cells treated with TAT-Cx43 or αCT-1 for 72 h. The left blot in (**d**) represents polarized CFTR KD treated with TAT-Cx43, αGA or both for 3 h, while the right blot shows CFTR KD cells treated with CBX, TAT-Cx43, αCT-1 or CBX + TAT-Cx43 and CBX + αCT-1 for 3 h. Fibronectin band intensity was quantified for all collected secretomes (**b** 3–8 biological replicates, 72 h: **p = 0.0014, ****p < 0.0001, 3 h: **p < 0.01 (αGA *p* = 0.0043, CBX *p* = 0.0004, αGA + TAT-Cx43 *p* = 0.0041, αGA + αCT-1 *p* = 0.0039, CBX + TAT-Cx43 *p* = 0.0002, CBX + αCT-1 *p* < 0.0001). **e**, **f** Representative top views of the apical epithelial surface from 3D reconstructed confocal images of polarized Calu-3 cells apically infected with 1 × 10^5^ CFU of PAO1 (red). The adherent PAO1 was analyzed 1 h post-infection in CFTR KD cells, basally treated or not with AMP-CP, TAT-Cx43, or αCT-1 for 72 h (**e**), and CTL cells after basal treatment with NECA for 72 h (**f**). Nuclei were stained with DAPI (blue). Scale bar: 40 μm. **g** The signal intensity of adherent PAO1 was normalized to DAPI staining to account for cell number (3–4 biological replicates, *n* = 8–27, ****p < 0.0001). *n* represents the total number of analyzed images per condition (**a**, **g**). Data are presented as mean ± s.e.m.; two-tailed Mann–Whitney test and one-way ANOVA test (**a**, **b**, **g**).

Connexin 43 may represent a target to reduce apical secretion of fibronectin in CF HAECs. In this context, connexin mimetic peptides are valuable experimental tools for blocking Cx43 activity^40^. CFTR KD Calu-3 cells were treated with TAT-Cx43_266–283_ (TAT-Cx43) and αCT-1, two membrane-permeant mimetic peptides with sequences corresponding to different parts of human Cx43 C-terminus^41,42^. Neurobiotin uptake experiments confirmed that both peptides block Cx43 HC activity (Fig. 7a). Next, we studied in CFTR KD cells the effects of αCT-1 or TAT-Cx43 on apical fibronectin secretion as a function of time of treatment. To this end, fresh peptides were added to the basal side of the CFTR KD epithelium every 24 h for up to 72 h of treatment. We also included in these experiments two conventional chemical connexin channel inhibitors, αGA and carbenoxolone (CBX), as confirmed by dye coupling (Supplementary Fig. 8g). A first series of experiments showed that detection of fibronectin in the apical secretomes decreased with time in CFTR KD cells treated with αCT-1 or TAT-Cx43 (Fig. 7b, c), reaching significance at 72 h. These results, which are consistent with the kinetics needed for AMP-CP to turn down CD73-dependent signaling, underline that prolonged targeting of Cx43 HCs abrogates the adenosine-fueled fibronectin secretion in CFTR KD cells. In a second series of experiments, we tested the effect of αGA and CBX on apical secretion of fibronectin. Again, the connexin channel inhibitors strongly decreased apical fibronectin detection, but the effect was observed already after 3 h of treatment (Fig. 7b, d). These observations indicate that targeting Cx43 channels is an efficient approach to restrict fibronectin production at the luminal side of CF HAECs.

### Targeting Cx43 reduces *P. aeruginosa* adhesion to the CF airway epithelium

Apical deposition of fibronectin drives the formation of bacterial docking stations at the surface of CF epithelium, which facilitates *Pa* adhesion^19,24^. This led us to explore if targeting Cx43 can efficiently prevent *Pa* attachment to the CFTR KD epithelium. To this end, Calu-3 cells grown at ALI were apically infected with 10^5^ CFU PAO1, a *Pa* laboratory strain. PAO1 adhesion was analyzed by confocal microscopy using a specific antibody against the bacteria. In line with our previous findings, CFTR KD cells showed increased PAO1 adhesion 1 h post-infection (Fig. 7e, g, NT condition). Modulation of adenosine signaling in CFTR KD cells with AMP-CP (Fig. 7e) and in CTL cells with NECA (Fig. 7f) differently affected PAO1 binding to the cell surface. Consistently with their effects on fibronectin secretion, activation of P1 receptors in CTL cells increased PAO1 adhesion, whereas CD73 inhibition in CFTR KD prevented its adhesion (Fig. 7g). Importantly, treatment with TAT-Cx43 or αCT-1 for 72 h reduced PAO1 adhesion to the surface of CFTR KD cells (Fig. 7e, g) in keeping with the effects of the Cx43 hemichannel channel blockers on apical fibronectin secretion.

**Fig. 8:**
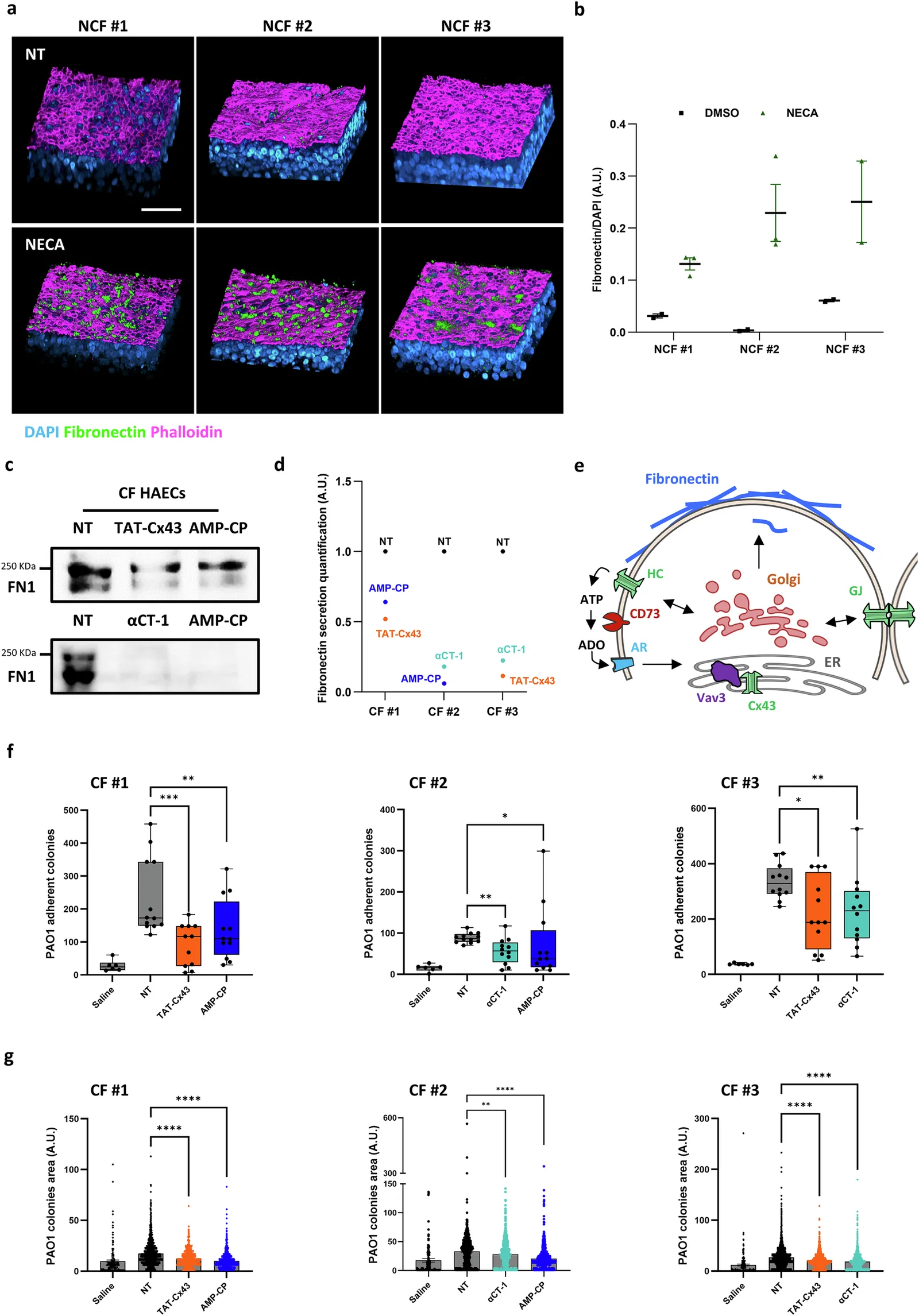
Cx43 targeting regulates fibronectin production and the associated PAO1 adhesion in CF primary HAECs. **a** Representative 3D projection of z-stack confocal images providing a comprehensive view of the apical deposition of fibronectin (green) in 3 non-CF (NCF) primary HAECs treated with NECA for 72 h. Actin filaments are stained with phalloidin (magenta) and nuclei with DAPI (blue). **b** The intensity of fibronectin signal was normalized to DAPI staining to account for cell number. Data are from 3 independent NCF donors, shown separately. Scale bar: 40 μm. **c**, **d** Representative Western blots depicting fibronectin (FN1) detection in the apical secretome collected 24 h after incubation of saline on top of fully differentiated F508del-CFTR (CF) primary HAECs basally treated with AMP-CP, TAT-Cx43, or αCT-1 for 72 h. Western blot images shown in (**c**) are representative of 2 independent donors and illustrate all conditions tested. **d** Shows the quantification of fibronectin band intensity measured in secretomes collected from 3 independent CF donors. Each treatment was tested in 2 different donors. **e** Graphical summary of the proposed model linking persistent Cx43, secretory axis impairment, and apical fibronectin deposition in CF HAECs. HC hemichannel, GJ gap junction, ER endoplasmic reticulum, ADO adenosine, AR adenosine receptor. Image created with BioRender.com and Servier Medical Art (https://smart.servier.com, licensed under CC BY 4.0 (https://creativecommons.org/licenses/by/4.0/)). **f**, **g** The apical secretomes of CF primary HAECs treated with AMP-CP, TAT-Cx43, or αCT-1 were collected after 24 h and used to coat coverslips that were then infected with 10^5^ CFU of PAO1. The number of PAO1 adherent colonies was quantified, data for each donor are shown separately (**f** *n* = 6–12, CF #1, ***p = 0.0005, **p = 0.0064; CF #2, **p = 0.0017, *p = 0.0371; CF #3, *p = 0.0292, **p = 0.0083) and their areas (**g** *n* = 6–12, CF #1, ****p < 0.0001; CF #2, **p = 0.0023, ****p < 0.0001; CF #3, ****p < 0.0001) using analysis particle function. The saline condition represents the adhesion of PAO1 to coverslips coated with control saline. *n* represents the total number of analyzed images per condition (**f**, **g**). Data are presented as box-and-whisker plots (**f**, **g**). Two-tailed Mann–Whitney test (**f**, **g**).

Primary CF HAEC cultures exhibit apicobasal polarity defect indicated by reduced TEER (Supplementary Fig. 6c) and deposition of fibronectin to their apical surface^19,24^. We examined the impact of modulating the adenosine pathway or blocking Cx43 channels on ectopic fibronectin detection in fully differentiated cultures derived from 3 NCF and 3 CF donors. On one hand, treatment of NCF cultures with NECA for 72 h stimulated the apical production of fibronectin, as revealed by immunostaining and 3D projection of confocal z-stack images (Fig. 8a, b). On the other hand, inhibiting adenosine production by AMP-CP or directly targeting Cx43 with TAT-Cx43 or αCT-1 in CF cultures for 72 h induced a robust decrease in apical fibronectin detection in the secretomes by Western blot (Fig. 8c, d). These results indicate that a Cx43-mediated adenosine signaling stimulates the apical secretion of fibronectin in CF HAECs. A summary diagram of the findings obtained so far is shown Fig. 8e.

To study *P. aeruginosa* adhesion, apical secretomes were collected from CF HAEC cultures treated with AMP-CP, αCT-1, or TAT-Cx43 in saline and coated on glass coverslips. As a negative control, coverslips were also coated with non-conditioned saline. PAO1 adhesion to these coated surfaces was monitored after 1 h, as previously described^19^. Representative images of PAO1 adhesion to the coated coverslips with the various secretomes are shown in Supplementary Fig. 9. The number of adherent colonies (Fig. 8f) as well as their areas (Fig. 8g) in response to the different treatments were quantified for each CF HAEC culture. In comparison to saline alone, PAO1 adhesion was significantly increased when coverslips were coated with CF secretomes. Like our findings on polarized CFTR KD Calu-3 cells, CD73 inhibition or Cx43 targeting in CF primary HAECs significantly reduced PAO1 adhesion to the secretomes-coated surfaces. These results confirm in primary cultures the deleterious effect of a sustained adenosine signaling in apical fibronectin production and demonstrate that Cx43 represents a novel target to restore apicobasal polarity and to prevent bacterial binding to the CF airway epithelium surface.

## Discussion

Partial or total loss of apicobasal polarity is witnessed in many diseases and is exploited by pathogens to infect tissues. Distinct receptor segregation between apical and basolateral domains underlies *Pa*-induced epithelial injury^43^. For example, *Pa* creates a local microenvironment by converting apical surfaces into protrusions composed of basolateral constituents that facilitate its colonization and internalization^44^. In this context, we recently reported that Vav3 overexpression in sterile CF HAECs or CRISPR-edited Calu-3 cell lines predisposes the airway epithelium to *Pa* adhesion by constructing an apical protein complex composed of β1 integrin and fibronectin^19^, two basolateral proteins that bind *Pa*^45–48^. We now show that this complex, which represents a key player in the ectopic communication between the host and its luminal environment, is sustained over time through a Cx43-dependent adenosine signaling. Furthermore, we demonstrate that targeting Cx43 channels with mimetic blocking peptides prevents *Pa* adhesion to the CF airway epithelium via inhibition of luminal fibronectin accumulation.

Vav3 exhibits a sophisticated structural organization functioning as both a signaling adaptor and an enzymatic GEF^49,50^. A key regulatory node linking CFTR dysfunction to Vav3 overexpression involves the RNA-binding protein HuR that increases Vav3 mRNA stability^24^. Among its targets, we showed that HuR also stabilizes Cx43 mRNA, promoting the overexpression of the gap junction protein in primary CF HAECs as well as in CFTR KD Calu-3 cells during the polarization process. HuR-dependent stabilization of Cx43 mRNA has been previously reported in liver and intestinal cells, enhancing GJIC and shown to control the junctional network integrity^51,52^. Newly translated Cx43 are transported from the ER to the Golgi as monomeric form stabilized by ERp29^53^. Interestingly, this ER chaperone was also shown to facilitate CFTR trafficking^54^, a process altered in F508del-CFTR expressing cells. Cx43 exit from the ER is favored by GTP-loaded small-GTPases^14^, followed by its oligomerization in the trans-Golgi network and trafficking to the plasma membrane^11^. Strikingly, PLA revealed that Vav3 and Cx43 are in proximity in the ER. As a GEF that favors GDP/GTP exchange on small-GTPases, Vav3 overexpression could lead to the supply of oligomerized Cx43 available to form HCs and GJs by enhancing the GTP-restricted forms and interfering with ER/Golgi architecture by regulating cytoskeletal dynamic^33^. A naturally occurring Cx43 C-terminal isoform of 20 kDa generated via internal translation, which localizes to the Golgi apparatus, has been reported to regulate full-length Cx43 trafficking during epithelial-mesenchymal transition (EMT)^55–57^. Whether this isoform contributes to hemichannels and gap junction formation in CF HAECs remains to be determined. In addition to forming channels, Cx43 can anchor microtubule distal ends to GJs, which might influence cytoplasmic scaffolding properties in coupled cells^15^. Cx43 was indeed identified as a microtubule-binding protein in glioblastoma stem-like cells, contributing to cancer progression^58^. Knocking down Cx43 in Calu-3 cells not only blunts HC and GJ channel activity but also re-established spatial organization of the secretory axis, polarity, and barrier properties of the epithelium. Our results point to an interplay between Vav3 and Cx43, thus potentially influencing secretion pathways.

Apicobasal polarization in epithelial cells relies on tightly coordinated secretion pathways that direct cargo from the ER to specific membrane domains. Golgi positioning is critical in various aspects of cell polarization. In polarized epithelia, the Golgi localizes between the nucleus and apical membrane, with the Golgi ribbon positioned near the centrosome, facilitating directional vesicle trafficking^59–61^. Repositioning and fragmentation of the Golgi complex also occur during wound repair and differentiation^62^, key cellular processes that are perturbed in the pathological context of CF^63^. This perturbation was predicted by bioinformatics analysis of primary CF HAECs and CFTR KD cells and was confirmed at the cellular level with disorientation of the centrosome and dispersal of the Golgi ribbons. Several studies have demonstrated that Cx43 dynamically interacts with secretory pathway components, influencing ER-Golgi-centrosome-vesicle coordination^53,64–66^. For example, Golgi stacking alterations and reduced Golgi reorientation during collective cell migration have been reported in HeLa cells stably expressing Cx43^17^. Although the molecular mechanism remains to be investigated, our results suggest that elevated Vav3 expression and increased Cx43 expression in CF HAECs destabilize actin-microtubule dynamics, impairing Golgi structure, positioning, and polarized secretion. Indeed, Golgi morphology and polarized vesicular transport are supported by Golgi-originating actin and microtubules^67,68^. This destabilization may thus explain the ectopic accumulation of fibronectin to the CF airway epithelial surface while rescuing the secretion pathways by Cx43 knockdown redirects detection of the ECM protein at the basal side of the epithelium. Our results indicate that persistent Cx43 expression is a key player of apicobasal polarity breaking in the pathological context of CF. These findings position Cx43 as a critical rheostat that regulates cellular differentiation states, with perturbations preventing the cells from completing a transition process, thus compromising tissue repair and promoting pathological plasticity. This is consistent with the consensus that the CF airway epithelium does develop a partial EMT phenotype, where cells retain epithelial identity while acquiring mesenchymal traits^28,69–71^.

Emerging evidence suggests that eATP and purinergic signaling contribute to hybrid epithelial-mesenchymal phenotypes, positioning purinergic signaling as a key modulator of EMT^72,73^. On the one hand, EMT upregulates CD73, increasing extracellular adenosine, and on the other hand, adenosine stabilizes partial EMT^73^. We found increased CD73 expression and increased adenosine signaling in CFTR KD cells. Pharmacological manipulation of CD73 activity or P1 receptor activity was linked to the apical detection of fibronectin at the surface of the airway epithelium. Stimulation with NECA induced apical fibronectin in control cells within 72 h, while its disappearance from the CF secretome after CD73 inhibition by AMP-CP also required 72 h. In addition, NECA and AMP-CP modulated Vav3-Cx43 proximity in the ER as well as the delivery of functional connexons to the plasma membrane. Two connexins mimetic peptides (αCT-1 and TAT-Cx43), which blocked Cx43 HC activity^74^, prevented fibronectin detection at the apical surface of CFTR KD cells with similar kinetics. As undocked connexons can function as ATP-permeant membrane channels, overactive Cx43 HC may release the nucleotide to the extracellular space, providing substrates for CD73 and adenosine signaling. Coupling between P1 receptors and Cx43 HCs has been reported, for example, in astrocytic primary cultures under Alzheimer’s disease-like conditions^75^. This scenario is supported by the detection of enhanced ATP hydrolysis and total purine quantity measured in cell-free sputum from CF donors^76^. We also confirmed enhanced levels of adenosine in secretomes of CFTR KD cells. Thus, ATP release from Cx43 HCs would initiate an amplification loop whereby adenosine signaling strengthens the proximity of Vav3 and Cx43 in the ER, breaking the secretion pathways over time and thus maintaining the ectopic production of fibronectin. In fact, it has been reported in osteocytes that mechanical forces can activate the fibronectin receptor α5β1 integrin, which in turn opens Cx43 HCs^77^. This mechanism is supported by increased membrane tension and elevated levels of β1 integrin at the apical membrane of CFTR KD cells^20,22^. In addition, the C-terminus of Cx43 is known to interact with ZO-1, preventing undocked connexons aggregation into GJs^78^. In CF HAECs, the loss of ZO-1 at cell-cell contacts^21,63^ may also contribute to GJs formation and increase GJIC. It is noteworthy that widely used gap junction blockers (αGA and CBX) were, in terms of kinetics, more effective in blunting fibronectin apical detection than αCT-1 and TAT-Cx43. In the absence of specific inhibitors of Cx43-made gap junction channels that do not block Cx43 HCs, we cannot, however, rule out the contribution of GJIC to the altered secretion pathways in CF HAECs. Together, our results indicate that targeting eATP/CD73/adenosine pathways with Cx43 HCs blockers offers therapeutic strategies to resolve partial EMT in CF HAECs.

The airway epithelium possesses a range of defense mechanisms against pathogens, partly relying on a tightly regulated balance between ATP release and degradation. Recently, airway epithelial tuft cells activated by *Pa* infection were shown to release ATP via Pannexin 1 channels, triggering innate immune responses^79^. The role of eATP-dependent purinergic modulation is also well described in airway epithelial cells to modulate airway surface liquid volume and mucociliary clearance via regulation of chloride secretion^80^. This equilibrium is likely altered in CF HAECs, where enhanced Cx43 HC activity and CD73 expression would favor adenosine-P1 receptor signaling over P2 receptor activation. This scenario is in keeping with the report that inhibition of eATP hydrolysis restores P2Y receptor-dependent airway surface liquid production in CF HAECs^76^. Here, we show that blocking the Cx43 HCs-adenosine-P1 signaling with Cx43 mimetic peptides efficiently reduced *Pa* adhesion to the apical environment of CF HAECs. Thus, targeting Cx43 HCs in CF HAECs could circumvent the impaired airway epithelial defenses contributing to recurrent *Pa* infections, which are a major cause of morbidity and mortality in people with CF (pwCF).

The recent approval of triple-modulator therapy for CFTR represents one of the greatest advances in CF care by improving pulmonary function in pwCF. Despite improving their quality of life, clinical trials revealed variable responses to this drug formulation, with some individuals showing limited benefit as they remained infected with *Pa* over 3 years of treatment^81–83^. In addition, about 10% of pwCF are not eligible for triple-modulator therapy due to the class of CFTR mutation^84^. We recently reported that short-term triple-modulator therapy did not correct the apicobasal polarity defect in primary CF HAECs, which maintained fibronectin accumulation at their apical surface^23^. Moreover, analysis of available RNA-seq datasets indicates that Cx43 expression remains unchanged even 2 years after ETI therapy, suggesting that Cx43 dysregulation may persist despite long-term treatment^85^. Thus, pwCF may benefit from connexins-based therapeutic approaches either alone or in combination with CFTR modulators to eliminate apical fibronectin deposition, restore the defense capacity of HAECs, and prevent bacterial infections.

## Methods

### Human donor-derived airway epithelial cells

Primary cultures of HAECs were obtained from Epithelix Sàrl. Non-cystic fibrosis (NCF) and CF cells derived from bronchial biopsies were cultured on Transwell® inserts using MucilAir™ Culture Medium (Epithelix Sàrl) and differentiated at ALI for 2 months. Two series of primary cultures were used, with donors/patients' clinicopathological characteristics provided in Supplementary Tables 1 and 2.

### Cell culture

The Calu-3 airway epithelial cell line (ATCC® HTB-55™) was cultured in minimum essential medium (MEM) GlutaMAX™, supplemented with 1% non-essential amino acids (NEAA, 100X), 1% HEPES (1 M), 1% sodium pyruvate (100X), 10% heat-inactivated fetal bovine serum (FBS), and penicillin/streptomycin/amphotericin B. Cells were maintained at 37 °C in a humidified atmosphere with 5% CO₂. To obtain polarized Calu-3 cells, 1.75 × 10⁵ cells were seeded onto polyester Transwell® inserts (0.4 μm pore size, 0.33 cm² surface area; Costar®) and maintained under liquid–liquid interface conditions for 5 days. Upon reaching tight confluency (designated day 0, D0), cells were exposed to ALI and cultured for 14 days to achieve polarization (D14).

CFTR knockdown in Calu-3 cells (CFTR KD) was performed using CRISPR-Cas9 with sgRNA targeting sequence corresponding to nucleotides 117,504,274 to 117,504,296 in *CFTR* exon 2^31^. Cx43 knockdown (Cx43 KD) Calu-3 cells and their respective controls (scramble) were generated using CRISPR-Cas9 lentiviral transduction (GeneCopoeia™). Calu-3 cells expressing Cas9 (GFP^+^) were seeded at 6 × 10^4^ and infected lentiviral particles carrying either Cx43-targeting sgRNA (promoter: U6, reporter: mCherry, resistance marker: puromycin resistance gene, Cat.No.: LPPHCP207411L03-1-50-01) or scrambled sgRNA lentivirus (promoter: U6, reporter: mCherry, resistance marker: puromycin resistance gene, Cat.No.: LPPCCPCTR01L03-100-cs) in presence of polybrene (Sigma-Aldrich) for 4 h at 37 °C in humidified 5% CO_2_ atmosphere. Cells were selected with puromycin to enrich for double-positive GFP⁺/mCherry⁺ populations. All cell lines were confirmed to be free of mycoplasma contamination using the MycoAlert® Mycoplasma Detection Kit (Lonza).

### Experimental conditions

CD73 activity was inhibited by basal incubation with the CD73-specific inhibitor α,β-methyleneadenosine 5′-diphosphate (AMP-CP, 100 μM, Sigma-Aldrich). To activate adenosine receptors, the non-selective agonist 5′-(N-ethylcarboxamido) adenosine (NECA, 10 μM, Tocris) was applied to the apical, basal or both surfaces. Two pharmacological inhibitors, 18-α-glycyrrhetinic acid (αGA, 25 μM, Sigma-Aldrich) and carbenoxolone (CBX, 25 μM, Sigma-Aldrich), were used to inhibit Cx43 channel activity. The hemichannel activity of Cx43 was selectively inhibited using two mimetic peptides, TAT-Cx43_266–283_ (50 μM, GenScript) and αCT-1 (5 µΜ, synthesized by the “Peptides” Core Facility of the Faculty of Medicine, University of Geneva, Geneva).

### RNA-sequencing

Total RNA was extracted from primary HAEC cultures derived from 7 CF and 6 NCF donors, non-wounded or undergoing repair^30^. Mechanical wounding was performed using an airbrush linked to a pressure regulator^86^. Total RNA was also isolated from polarized CTL and CFTR KD Calu-3 cells grown on Transwell® inserts^87^. RNA samples were processed using the TruSeq stranded mRNA library preparation protocol (Illumina). Libraries were sequenced on an Illumina HiSeq 4000 platform using a single‑end 50 bp configuration. Sequencing yielded 16–29 million reads per sample, consistent with the recommended depth for bulk gene‑level expression profiling. FastQ reads were mapped to the ENSEMBL reference genome (GRCh38.80) using STAR version 2.4.0j with standard settings, except that any reads mapping to more than one location in the genome (ambiguous reads) were discarded. The average mapping rate was 97.67% for primary HAECs and 95.8% for Calu‑3 cells, indicating high alignment quality across sample types. All reads overlapping the exons of each unique gene model were reported using featureCounts version 1.4.6-p1. Gene expressions were reported as raw counts and, in parallel, normalized in RPKM in order to filter out genes with low expression value (1 RPKM) before calling for differentially expressed genes. Library size normalizations and differential gene expression calculations were performed using the package edgeR designed for the R software. Genes were ranked by their calculated fold-changes (decreasing ranking). A gene set analysis using the GSEA package Version 2.2 (Broad Institute) was used to analyze the pattern of differential gene expression between the two groups. Gene set permutations were performed 1000 times for each analysis. The normalized enrichment score (NES) was calculated for each gene set. GSEA results with a nominal FDR < 0.05 and abs(NES) > 1 were considered significant.

### 3′UTR region analysis

The AU-rich element motifs in the 3′UTR region of Cx43 mRNA were mapped using the public database AREsite2 (http://rna.tbi.univie.ac.at/AREsite).

### Ribonucleoprotein immunoprecipitation (RNP-IP)

Calu-3 cells, treated or untreated with CMLD-2 (20 μM, Sigma-Aldrich), were lysed in polysome lysis buffer (20 mM Tris-HCl, pH 7.5, 5 mM MgCl₂, 150 mM NaCl, 1 mM DTT, 0.5% NP-40, cOmplete™ Protease Inhibitor Cocktail (Roche), and RiboLock RNase Inhibitor (100 U)). Lysates were clarified by centrifugation at 10,000 × *g* for 15 min at 4 °C, and protein concentration was determined using the Pierce BCA Protein Assay Kit (Thermo Fisher Scientific). For preclearing, 800 µg of total protein was incubated with 50 µl of Protein A/G magnetic beads (PureProteome™) for 2 h at 4 °C. The supernatant was then incubated overnight at 4 °C with either anti-HuR antibody (IP HuR) or without (NoIP), in the presence of 1 µl DTT (1 M), 40 U RiboLock RNase Inhibitor, and 33 µl EDTA (0.5 M). To isolate HuR–mRNA complexes, 50 µl of Protein A/G magnetic beads were added and incubated for 1 h at 4 °C. Beads were subsequently washed with NT2 buffer (50 mM Tris-HCl, 150 mM NaCl, 1 mM MgCl₂, 0.05% NP-40) at 4 °C and resuspended in RLT buffer. The RNA bound to HuR was extracted using the RNeasy Mini Kit (Qiagen) according to the manufacturer’s protocol.

### RT-qPCR

Total RNA was extracted using the RNeasy Mini Kit (Qiagen) according to the manufacturer’s instructions. RNA concentration and purity were assessed using a NanoDrop 2000 spectrophotometer (Thermo Fisher Scientific). Prior to reverse transcription, genomic DNA contamination was eliminated using the gDNA Wipeout Buffer (Qiagen) by incubating samples for 2 min at 42 °C. Complementary DNA (cDNA) was then synthesized using the QuantiTect Reverse Transcription Kit (Qiagen). Quantitative reverse transcription PCR (RT-qPCR) was carried out on a StepOnePlus™ Real-Time PCR System using PowerUp™ SYBR™ Green Master Mix. mRNA expression levels were calculated as absolute values (2^−ΔCt^), normalized to 18s rRNA expression. All primers were obtained from Microsynth AG, and the specific sequences used are the following: Cx43 (forward 5′–3′ CGGTTGAGTCAGCCTGG; reverse 5′–3′ TCTCTTCCTTTCGCATCACATA), Cx26 (forward (F)/5’–3’ TGGCCTACCGGAGACAT; reverse (R)/5′–3′ CGTCGTACATGACATAGAAGAC), Cx30 (F/5′–3′ TTCCTTTACAATGGGTACCA; R/5′–3′ TTAAGCAGCATGCAAATCAC), Cx31 (F/5′–3′ CGACAACGCAGGCAAGAA; R/5′–3′ GTAGGTCGGGCAATGTAGCAGT) and 18s (F/5′–3′ GTAACCCGTTGAACCCCATT; R/5′–3′ CCATCCAATCGGTAGTAGCG). To assess Cx43 mRNA stability, cells were treated with actinomycin D (Sigma-Aldrich) at a final concentration of 5 µg/mL to inhibit transcription. At indicated post-treatment times (0, 2, and 4 h), total RNA was extracted and Cx43 mRNA expression monitored by RT-qPCR. The relative abundance of mRNA at each time point was calculated and expressed relative to time zero (*t* = 0 h).

### Immunofluorescence

Primary HAECs and Calu-3 cells were fixed with 4% PFA for 30 min at room temperature (RT) and permeabilized for 15 min at RT with 0.1% Triton 100X buffer. Cryosections of non-wounded CF and NCF primary HAECs, embedded in Tissue-Tek® O.C.T. compound, were fixed in 4% paraformaldehyde (PFA) for 30 min on ice. Following fixation, sections were permeabilized with 0.1% Triton X-100 in phosphate-buffered saline (PBS) for 15 min at RT. After permeabilization, non-specific binding sites were blocked by incubating sections in 1% BSA in PBS for 30 min at RT. Samples were then incubated overnight at 4 °C with primary antibodies specific to the proteins of interest (detailed in Supplementary Table 3). After several washes with PBS, secondary antibodies conjugated to AlexaFluor® 488, AlexaFluor® 568, or AlexaFluor® 647 (depending on the species of the primary antibody) were applied for 1 h at RT. To amplify the Cx43 signal in primary HAECs, the TSA Opal™ method (Akoya Biosciences) was employed according to the manufacturer’s instructions. This method is based on tyramide signal amplification (TSA), in which an HRP-conjugated secondary antibody catalyzes the deposition of fluorescently labeled tyramide molecules (Opal dyes) at the site of the primary antibody. Following permeabilization, endogenous peroxidase activity was quenched using a multiplex HRP blocking solution, followed by blocking with BSA. Samples were then incubated overnight at 4 °C with the primary antibody against Cx43. The next day, an HRP-conjugated secondary antibody (listed in Supplementary Table 3) was applied for 1 h at RT, followed by PBS washes. Sections were then incubated with Opal™ 570 reagent (Akoya Biosciences) for 30 min at 37 °C. DAPI was used for counterstaining to visualize cell nuclei. Immunofluorescence images, including z-stack reconstructions, were captured using an LSM800 confocal microscope (Zeiss) and processed using ZEN software (Zeiss).

Quantification of fluorescent signals was performed using ImageJ (NIH). For each region of interest (ROI), we measured the *integrated density* of the marker channels and their corresponding DAPI channels. To correct variations in cell number across images, the *integrated density* of the markers was normalized to the DAPI signal. Multiple independent ROIs were acquired either from the same culture well, from separate cryosections, or from different Transwells®. Each quantified image is represented as a single point on graphs. The total number of images analyzed per condition represents the cumulative number of images (*n*) across all donors or biological replicates.

### Duolink® proximity ligation assay (PLA)

Proximity ligation assay was performed to monitor protein–protein proximity using the Duolink® In Situ Red Starter Kit (Sigma-Aldrich) according to the manufacturer’s instructions. Briefly, after treatment, polarized Calu-3 cells were fixed with 4% PFA for 30 min at RT and permeabilized with 0.1% Triton X-100 in PBS for 15 min. To visualize protein proximity in relation to the ER, cells were prelabeled with the ER-tracker blue-white DPX dye (Thermo Fisher Scientific). Briefly, cells were incubated with the dye (1 µM) in Hankʼs Balanced Salt Solution with calcium and magnesium (HBSS/Ca^2+^/Mg^2+^, Gibco) for 30 min at 37 °C, then washed twice with PBS to remove excess dye. After labeling, cells were processed further for the proximity ligation assay. First, non-specific binding was blocked with Duolink® In Situ Blocking Solution for 1 h at RT. Next, cells were incubated overnight at 4 °C with primary antibodies specific for the proteins of interest (detailed in Supplementary Table 3). Following primary antibody incubation, cells were washed in Duolink® Wash Buffer A and incubated with PLA probes (anti-rabbit PLUS and anti-mouse MINUS) for 1 h at 37 °C. Probes were then ligated with the Duolink® In Situ Ligation Kit by incubating for 30 min at 37 °C to form a circular DNA strand only when the two target proteins were in close proximity (<40 nm). Following ligation, the amplification step was performed using the Duolink® In Situ Amplification Kit, which involves adding fluorescently labeled oligonucleotides. This amplifies the signal and allows visualization of protein–protein interactions via fluorescence microscopy. PLA signals were imaged using an LSM800 confocal microscope (Zeiss). The PLA-positive spots (indicating protein–protein proximity) were quantified using ImageJ.

### Transmission electron microscopy (TEM)

Polarized Calu-3 cells were fixed in 2.5% glutaraldehyde and 2% paraformaldehyde (PFA) in 0.1 M sodium cacodylate buffer, pH 7.4, for 1 h at RT. Samples were rinsed three times in 0.1 M Caco buffer and post-fixed with 1% osmium tetroxide and 1.5% ferrocyanide for 1 h, immediately followed by 1% osmium tetroxide in 0.1 M Caco alone for an additional 1 h at RT. After two washes for 5 min in double distilled water (ddH2O) samples were en block stained with 1% uranyl acetate in water overnight at 4 °C. Samples were then rinsed twice for 5 min in ddH_2_O and dehydrated through a graded ethanol series (2 × 50%, 70%, 90%, 95%, and 2 × 100% for 10 min each). Samples were infiltrated into Epon resin by graded infiltration of resin diluted with ethanol: 1:2, 1:1, and 2:1 for 30 min each, then pure Epon resin 2 × 30 min and pure Epon for 2–4 h. The membrane was cut out from the insert, flat-embedded and polymerized for 48 h at 60 °C. Ultrathin sections (50 nm) from cured resin block were cut using Leica EM UC7 ultramicrotome (Leica) and diamond knife (DiATOME) and collected on copper hexagonal mesh EM grids coated with formvar support plastic film. Sections were examined using a Tecnai 12 G2 transmission electron microscope (FEI) operating at 80 kV acceleration voltage and equipped with a side-mounted MegaView III CCD camera controlled by iTEM acquisition software (both Olympus-Soft Imaging Solution).

### Wound healing assay and cell proliferation assay

Calu-3 cells were seeded onto glass coverslips placed in 24-well plates and cultured until confluence. A linear scratch was introduced into the monolayer using a sterile 200 μL pipette tip, and wells were gently washed with PBS to remove cell debris and non-adherent cells. The wound healing was followed at 1, 3, 8, and 24 h post-scratch. Images were acquired using an AMG EVOS microscope, and the wound area was measured with the ImageJ macro “ColonyArea.” The percentage wound closure was quantified relative to the 1 h measurement.

Cell proliferation was assessed using a colorimetric MTT assay. Calu-3 cells were seeded in 24-well plates at equal density and cultured under standard conditions. At 24, 48, and 72 h post-seeding, cells were incubated with MTT reagent (0.5 mg/mL; Sigma-Aldrich) for 1 h at 37 °C. Following incubation, the medium was removed, and the resulting formazan crystals were solubilized in DMSO. Absorbance was measured at 570 nm using a microplate reader (Hidex).

### Golgi and centrosome orientation analysis

Golgi and centrosome orientation were determined in leading-edge cells after wounding primary HAECs or Calu-3 cells grown as monolayers on glass coverslips. Cells were fixed at 3 h (Calu-3) or 5 h (primary HAECs) post-wounding with PFA, and immunostained for giantin or pericentrin, followed by DAPI counterstaining. The orientation of the Golgi and centrosome was manually determined in relation to the nucleus and the wound edge. Giantin and pericentrin fluorescent signals were classified based on their positioning relative to the nucleus: forward (toward the wound), backward (away from the wound), lateral (perpendicular to the wound edge), or none when Golgi staining overlapped with the nucleus. For each image, the percentage of cells displaying each orientation was calculated relative to the total number of leading-edge cells. Each data value in the graphs corresponds to a single analyzed image. Microtubule branching was quantified using the “skeletonize” function in ImageJ to convert binarized microtubule images into skeleton representations, which enables structural tracing and analysis of filamentous networks. The skeleton metrics (branch counts, branch lengths, junctions, and junction voxels) were quantified using the Analyze Skeleton plugin in ImageJ.

Golgi orientation relative to the wound edge was also quantified using a custom ImageJ macro. Nuclei and Golgi were segmented from fluorescence images using automated thresholding and size filtering, and centroid coordinates were extracted. The wound edge was manually traced, and for each nucleus, the nearest point along the wound edge was identified. Cells located more than 150 µm from the wound edge were excluded from analysis. For each included cell, the nearest Golgi centroid was assigned. Golgi orientation was defined by the angle between two vectors: a nucleus-to-Golgi vector and a nucleus-to-wound vector. Angles were calculated using vector dot and cross products and expressed in degrees. Absolute angle values ranging from 0° to 180° were used for quantification, where smaller values indicate Golgi alignment toward the wound edge.

The same custom macro was used to quantify centrosome orientation. Centrosomes were identified using triangle thresholding with a minimum size cutoff of 5 pixels. Centroid coordinates were extracted for all segmented objects. The wound edge was manually traced using the freehand line tool, and its coordinates were used as a spatial reference. For each nucleus, the shortest distance to the wound edge was calculated, and only cells within 100 µm of the wound were included. Each nucleus was assigned to its nearest centrosome based on Euclidean distance. Centrosome orientation was defined as the angle between the vector connecting the nuclear centroid to the centrosome and the vector connecting the nucleus to the closest point on the wound edge. Angles were calculated using a two-dimensional “atan2” function and expressed in degrees. Absolute angle values (0–180°) were used for all analyses, where 0° indicates centrosome orientation toward the wound edge and 180° indicates orientation away from the wound.

### Golgi dispersal analysis

Golgi dispersal was quantified using ImageJ by threshold-based segmentation of the Golgi marker fluorescence followed by particle analysis. Raw images were converted to 8-bit grayscale images that were subjected to an identical thresholding method and parameters to generate binary masks of Golgi structures. Segmentation was based on pixel connectivity, allowing separation of continuous Golgi structures into discrete elements. The “analyze particles” function was then applied to identify each discrete particle as an individual Golgi signal. The total number of particles per area was referred to as the dispersed Golgi signal counts. Identical acquisition and analysis parameters were applied to all samples.

### Western blot

Protein extraction was performed using Nonidet-P40 (NP-40) lysis buffer composed of 150 mM sodium chloride, 50 mM Tris-HCl (pH 8.0), 1% NP-40, and the cOmplete™ Protease Inhibitor Cocktail (Roche). Lysates were centrifuged at 14,000 × *g* for 15 min, and protein concentrations were determined using the Pierce™ BCA Protein Assay Kit (Thermo Fisher Scientific). Equal amounts of protein were resolved by SDS-PAGE and transferred onto a Macherey-Nagel™ NCP Porablot nitrocellulose membrane. Membranes were blocked for 1 h at RT in 3% BSA diluted in PBS-Tween (0.01%) and subsequently incubated overnight at 4 °C with primary antibodies (listed in Supplementary Table 3) under gentle agitation. Loading consistency was assessed by Ponceau S staining and confirmed with GAPDH immunoblotting. Following primary antibody incubation, membranes were washed with PBS-Tween (0.01%) and incubated with horseradish peroxidase (HRP)-conjugated secondary antibodies. Protein detection was carried out using the SuperSignal™ West Pico PLUS chemiluminescent substrate (Thermo Fisher Scientific), and signals were visualized using the Fusion chemiluminescence imaging system (Vilber). Band intensities were analyzed with Quantity One Analysis Software (Bio-Rad).

### Transepithelial ion transport and electrical resistance (TEER) measurements

For transepithelial ion transport measurements, Transwell® inserts were mounted in Ussing chambers and short-circuit current (Isc) was recorded using a VCC MC6 voltage–current clamp amplifier (Physiologic Instruments). Data acquisition was performed using a DI-720 interface (DataQ Instruments) and Acquire & Analyze software (v2.3, Physiologic Instruments). The basolateral chamber was filled with Krebs solution (in mM: 115.5 NaCl, 25 NaHCO₃, 2.4 K₂HPO₄, 0.4 KH₂PO₄, 1.2 CaCl₂·2H₂O, 1.2 MgCl₂·6H₂O, and 10 glucose), while the apical chamber contained a low-chloride Krebs solution (in mM: 100.5 Na-gluconate, 15 NaCl, 25 NaHCO₃, 2.4 K₂HPO₄, 0.4 KH₂PO₄, 1.2 CaCl₂·2H₂O, 1.2 MgCl₂·6H₂O, and 10 glucose). Solutions were maintained at 37 °C and continuously gassed with 95% O₂ and 5% CO₂. The transepithelial potential difference was clamped to 0 mV, and the resulting Isc was recorded. cAMP-dependent currents, including CFTR-mediated currents, were stimulated by the addition of forskolin (10 µM; Sigma) and IBMX (100 µM; Sigma) to both chambers. CFTR activity was inhibited by apical application of Inh-172/GlyH-101 (20 µM; Merck).

TEER values were recorded from primary HAECs or Calu-3 cells cultured on Transwell® inserts at ALI using an electrical resistance meter (EVOM; World Precision Instruments). Data are represented as ohms × square centimeter (Ω.cm²) after subtracting the background of cell-free filters. Higher values indicate intact TJs and a well-formed epithelial barrier.

### Dye uptake assay

To assess hemichannel (HC) activity, Calu-3 cells were polarized at ALI and were apically incubated with neurobiotin (Vector Laboratories), a small (MW ~286 Da), biotinylated tracer permeable to open HC. Cells were first washed twice with sterile PBS. Neurobiotin (Vector Laboratories) was prepared at a final concentration of 0.5 mg/ml in a Krebs-Ringer buffer (KRB) solution (pH adjusted to 7.4). To maximize HC opening, the epithelial surface was pre-exposed to a Ca²⁺-free KRB solution, in which CaCl₂ was omitted, and 3 mM EGTA was added. Cells were then apically incubated with neurobiotin-containing KRB (with or without Ca^2+^) solution for 15 min at 37 °C. Following dye uptake, cells were washed three times with PBS and fixed with 4% paraformaldehyde (PFA) for 30 min at RT. Fixed cells were blocked with 1% BSA for 30 min, and incubated with streptavidin-Alexa Fluor 647 (Thermo Fisher Scientific) in PBS with 1% BSA for 1 h at RT. Samples mounted using VECTASHIELD antifade mounting medium (Vector Laboratories) and imaged using a Zeiss LSM 800 confocal microscope. Identical acquisition settings were used for all conditions within each experiment. The mean fluorescence intensity was measured using ImageJ.

### Adenosine assay

Apical adenosine was quantified using the MET-5090 Adenosine Assay Kit (Cell Biolabs) according to the manufacturer’s instructions in CTL and CFTR KD Calu-3 cells polarized at ALI. To fully open Cx43 HCs, cells were apically incubated with 100 µL of calcium-free extracellular PBS for 3 h at 37 °C. Where indicated, cells were pretreated with 25 µM αGA for 30 min prior to calcium-free incubation and maintained in the solution until supernatant collection. Apical supernatants were collected and processed for adenosine quantification according to the kit instructions, and fluorescence was measured at 590/20 nm on a microplate reader (Hidex).

### Neurobiotin microinjection for gap junctional intercellular communication

To assess GJIC, neurobiotin was introduced into individual cells by intracellular microinjection. Micropipettes were pulled from borosilicate glass capillaries micropipette using a PC-100 puller (Narishige) and were backfilled with 2% neurobiotin solution (Vector Laboratories). Using a micromanipulator, the tracer was injected into a single Calu-3 cell cultured at ALI, and diffusion was allowed to proceed for 3 min in KRB. Up to 5 microinjections were performed per culture within a 15 min time window. Cells were then washed twice with PBS and fixed with 4% PFA for 30 min at RT. Following fixation, cells were blocked with 1% BSA for 30 min and incubated with streptavidin-Alexa Fluor 647 (Thermo Fisher Scientific) in PBS with 1% BSA for 1 h at RT. Samples mounted using VECTASHIELD antifade mounting medium (Vector Laboratories) and imaged using AMG EVOS microscope (Thermo Fisher Scientific). Dye coupling was quantified by counting the number of neurobiotin-positive neighboring cells surrounding the injected cell, using identical acquisition parameters and a consistent intensity threshold across all conditions. Only cells exhibiting a signal clearly above background and spatially connected to the injected cell were included, in line with standard practices. No differences in the extent of dye coupling was observed between the first and the last injections made within the 15 min time window.

### *Pseudomonas aeruginosa* adhesion assay

*Pseudomonas aeruginosa* (*Pa*) adhesion was assessed using the PAO1 laboratory reference strain obtained from our institutional strain collection^88^. Bacteria were cultured overnight in Lysogeny Broth (LB) at 37 °C with shaking. Calu-3 cells were pre-treated for 72 h with mimetic peptides or pharmacological modulators. 24 h prior to infection, the culture medium was replaced with antibiotic-free medium. Cells were then apically infected with 1 × 10⁵ colony-forming units (CFU) of PAO1 and incubated for 1 h at 37 °C in a 5% CO₂ humidified atmosphere. Following infection, cells were washed thoroughly with PBS to remove non-adherent bacteria and fixed with 4% paraformaldehyde (PFA) for 30 min at RT. Cells were then permeabilized, and adherent bacteria were visualized using a *Pa*-specific primary antibody. Confocal imaging was performed using a Zeiss LSM 800 microscope, and bacterial adhesion was quantified using ImageJ. For CF primary HAECs, apical secretomes were collected 24 h after saline incubation. The resulting conditioned saline was used to coat sterile glass coverslips for 1 h at 37. These coated coverslips were subsequently infected with PAO1 (1 × 10⁵ CFU) under the same conditions described above. After 1 h of incubation, coverslips were washed, fixed, stained, and analyzed using the same imaging and quantification protocol as described earlier.

### Statistics and reproducibility

Data are presented as mean ± standard error of the mean (s.e.m.). All experiments were performed with a minimum of three independent replicates. Sample sizes and number of replicates are provided in the corresponding figure legends. Statistical analyses were conducted using GraphPad (Prism). Comparisons between two groups were performed using an unpaired two-tailed Student’s *t* test, the non-parametric Mann–Whitney *U* test or one-way ANOVA, as appropriate. For comparisons involving more than two groups, two-way ANOVA was used. Not significant (ns), *p* > 0.05; *p < 0.05; **p < 0.01; ***p < 0.001; and ****p < 0.0001.

### Reporting summary

Further information on research design is available in the Nature Portfolio Reporting Summary linked to this article.

## Supplementary information

Transparent Peer Review File

Supplementary Information

Reporting Summary

## Data Availability

All data supporting the findings of this study are available from the corresponding author upon reasonable request. The source data file for all datasets generated and analyzed during this study is deposited in our institutional data repository Yareta: https://doi.org/10.26037/yareta:ii6b2gb7bfgfzbrjd6tky2l73q). Supplementary Figs. and tables are provided in the Supplementary Information file. RNA sequencing data were generated in previous studies^30,87^ and are available in Gene Expression Omnibus (GEO) under accession numbers GSE127696 and GSE133495. Uncropped Western blot scans, including size markers, are provided in the Supplementary Information.
