# Supplementary material for: Cx43 levels guide apicobasal polarity in regenerating airway epithelial cells
Source: Commun Biol. 2026 Jun 12;9:1264. doi: 10.1038/s42003-026-10413-7 (PMC13616896; doi:10.1038/s42003-026-10413-7)
Supplement: Supplementary file 1 [file 42003_2026_10413_MOESM1_ESM.pdf]

nature portfolio

Peer Review File

Cx43 levels guide apicobasal polarity in regenerating airway epithelial cells

Corresponding Author: Dr Mehdi Badaoui

This file contains all reviewer reports in order by version, followed by all author rebuttals in order by version.

Version 0:

Reviewer comments:

Reviewer #1

(Remarks to the Author)
The manuscript entitled “Cx43 levels guide apico-basal polarity in regenerating airway epithelial cells” was submitted by Badaoui and colleagues.
In this study, the authors propose that Cx43 plays a central regulatory role in the altered polarization of CF bronchial epithelial cells. To do so, they showed that Cx43 expression persists in primary CF bronchial cells during epithelial regeneration and during polarization of CFTR KD Calue-3 cells, due to post-transcriptional stabilization of mRNAs, and with a mainly apical localization of proteins. Their results suggested that persistent Cx43 expression disrupts the ER-Golgi axis with Golgi fragmentation and destabilizes microtubule organization, leading to impaired tight junction network and apical fibronectin secretion. They demonstrated that CX43 depletion in CFTR KD Calue-3 cells reorganizes the ER-Golgi axis, reduces Golgi fragmentation and dispersion, improves the tight junction network and trans-epithelial electrical resistance, and redirects fibronectin secretion to the basal pole of the epithelium. Overexpression of CD73, a membrane-bound ectonucleotidase involved in the conversion of extracellular ATP to adenosine, was demonstrated in CFTR KD Calue-3 cells, as was the involvement of P1 adenosine receptors in fibronectin secretion. They also showed that fibronectin expression and secretion result from the activation of P1 receptors by adenosine produced by CD73 from extracellular ATP, as well as increased adenosine-dependent activity of Cx43 in CFTR KD Calue-3 cells. All these findings led the authors to propose that persistent CX43 expression and increased activity fuel adenosine-dependent apical production and secretion of fibronectin in CF cells. This hypothesis was verified using connexin mimetic peptides and chemical inhibitors of connexin channels, which led to a decrease in fibronectin secretion. Finally, they demonstrated that modulation of P1 receptors alters the adhesion of the Pseudomonas aeruginosa PAO1 strain to the surface of Calue-3 cells, and validated their results using ALI cultures of non-CF and CF primary cells.

This is a very elegant and comprehensive study. The results are convincing. However, the reviewer has some concerns, mainly related to the presentation of the results and the methodology used for the quantifications.

For each presentation of immunofluorescence labeling with confocal microscope acquisition, it would be useful to present orthogonal projections of Z-stack images rather than (or in addition to) 3D projections in order to truly appreciate the location of the labels.

The reviewer does not understand the quantification of markers (Cx43, ZO-1, CD73, PAO1 adhesion) in relation to DAPI. In general, the various quantifications using ImageJ must be clearly and precisely explained. It is unclear what each point corresponds to in the graphs: for example, in Figure 1d, it is specified that cultures from 3 CF donors and 3 non-CF donors were analyzed, and a value of n=11-14 is given. What does this value of n correspond to? Is it the number of culture wells? The number of images analyzed? If so, how many different wells/images per patient? It is further specified that patient characteristics are given in Supplementary Table 1, which presents 5 non-CF subjects and 5 CF subjects. However, Figure S1 presents results obtained with 6 non-CF donors and 7 CF donors

The heat map in Figure 1 should show data for each patient with and without cystic fibrosis, and the graphs in Figure S1 would be easier to understand if they showed average values for each time point (rather than values for each patient), with a statistical analysis.
At what level relative to the wound were the photos taken in Figure 1? Was it in the previously damaged area, or behind it? This needs to be clarified.

There appears to be an error in Figure 1m, which shows CD73 expression instead of CX43 expression.

Regarding the polarization of Calu-3 cell cultures, this is achieved when the cells are confluent and then placed at the air-liquid interface. In this case, how do the authors explain the fact that, in the photos, there are many areas that do not show cells (figures 1l, 2d, 2f, 2h, 3c, 7i, S2, S3)?

Page 7 line 162-163: how to determine that the colocalization is mostly observed in cytoplasmic compartment rather than at the cell membrane with orthogonal projections of Z-stack images?

Figure 3: it is difficult to see a “perinuclear” organization of the ER and a fragmentation of the Golgi. When looking at the images, what allows the author to claim that the Golgi apparatus is fragmented?

Figure 4: The reviewer does not understand how the orientation of the organelles is determined. In relation to what? We assume that the orientation of the nucleus depends on the position of the cell in relation to the wound... but how many rows of cells border the wound? How is this standardized so that conditions can be compared? How can orientation be expressed as a percentage? What do the points in the graphs correspond to? Does Cx43 depletion alter the speed or orientation of cell migration? It would be interesting to add the statistical value of the Scr and Cx43 KD comparisons in the CTL condition.

Figure 5: how was determined the Golgi fragments counts (5b)? It would be interesting to add the statistical value of the Scr and Cx43 KD comparisons of TEER values in the CTL condition

Figure 6: how is standardized the quantification of positive cells between conditions (fig 6l)?

Figure 8: Do the values shown in graph 8d correspond to the quantification of the Western blot experiment in the same figure (8b)? Because it is specified in the caption that the Western blot was performed with cultures from two donors, and there are three donors in the graph.

Page 8, line 170: The reviewer does not understand the “anterograde” notion in this case

Reviewer #2

(Remarks to the Author)
The authors studied connexin 43, a gap junction protein, focusing on mRNA stabilization, expression, and its role in regenerating airway epithelial cells. They demonstrated that 3` UTR of Cx43 mRNA contains an ARE element and is stabilized by HuR during in vitro airway epithelial wound repair in Calu-3 cell model and primary human epithelial cells. They provide evidence that primary airway epithelia express Cx43 protein differentially (due to mRNA stabilization) in monolayers encoding pathological CF-causing alleles compared to monolayers originating from healthy donors. They present an equivalent finding using CFTR KD in Calu-3 model. They show that Vav3 (a GEF protein) colocalizes with Cx43 in the ER and, via small GTPases, may facilitate Cx43 trafficking to the plasma membrane. They also demonstrate an altered Golgi orientation and fragmentation during wound repair in CFTR-KD Calu-3 cells compared with control cells. Additionally, they show that overexpression of Cx43 protein leads to apical fibronectin deposition in epithelia with diminished CFTR expression. They provide evidence that this atopic deposition of fibronectin enhances adhesion of P. aeruginosa using CFTR KD Calu-3 and by the use of excretome studies in primary CF airway epithelial cells. Finally, they reveal a purinergic positive feedback loop between Cx43 and CD73 that further enhances atopic fibronectin expression, which can be mitigated by inhibiting CD73 or Cx43. The manuscript will be of great interest to the field of cystic fibrosis. Statistical analysis used in the described studies is adequate.

Questions and comments:
1) The efficiency of CFTR KD in Calu-3 cells would be desirable to show on protein levels (Western Blots) and whether Cx43 KD has any effect on the CFTR KD efficiency (Figure 5).
2) It would be preferable to demonstrate a nearly equivalent level of HuR protein expression in both CFTR KD and control Calu-3 cells to support the findings in Figures 2b and 2c. This would provide evidence that manipulating CFTR expression does not selectively or nonspecifically increase HuR protein levels.
3) The author states that cells shown in Figure 2d, 2f, and 2h are polarized. To claim full polarization of airway epithelial cells, the cells should appear as continuous monolayers on Transwell support, not patchy structures like those shown in Figure 2d, 2f, and 2h. Holes in monolayers, especially those demonstrated in Figure 2f on the CFTR KD immunofluorescent image, illustrate a growing non-polarized state. If cells were grown on coverslips, full polarization of cells cannot be claimed.
4) The author states that CFTR KD cells demonstrate enhanced intracellular Vav3/Cx43 proximity compared to control cells, as shown in Figure 2f and g. Is it possible that the CFTR KD cells grow more slowly and do not reach full polarization by the time of ALI D14? In my opinion, measuring comparable transepithelial electrical resistance between control and CFTR KD ALI monolayers—indicating full polarization regardless of the time on ALI—would provide a more accurate assessment of differences in Cx43-Vav3 co-localization and enhancement, if cells were grown on Transwells, not on coverslips.
5) Similarly, the difference between control and CFTR KD cell polarization states in experiments demonstrated by Figure 3 is questionable. Days on ALI do not determine the maximum polarization of a cell culture monolayer. Equivalent transepithelial electrical resistance would be a better method for assessing comparable cell polarization between control and CFTR KD monolayers, regardless of the days of polarization.
6) The author states that giantin immunostaining shows increased Golgi fragmentation and dispersal in CFTR KD cells polarized at ALI (Figure 3d). Actively dividing cells also display a dispersed Golgi structure, underscoring the importance of

imaging control and CFTR KD cell monolayers in a similarly well-polarized state.
7) An increase in microtubule branching is also a sign of dividing cells during mitotic spindle formation. The literature shows that loss of Cx43 triggers cells to enter the cell cycle and enhances cancer cell invasion. As I mentioned previously, CFTR KD cell growth or cell duplication may be compromised (slowed) by a non-specific mechanism (a non-specific effect of KD), resulting in decreased cell duplication and mitotic spindle formation. Additionally, Calu-3 cells are cancer cells with an altered growth rate, and Cx43 KD may not further increase their growth rate. (Figure 4.f) With CFTR KD, cell growth and microtubule branching may decrease, a reversal that can be achieved with Cx43 KD. It would be beneficial to repeat this experiment with non-cancerous, CFTR-expressing epithelial cells, such as 16HBE or primary cells, if possible. Furthermore, demonstrating similar cell duplication times between control and CFTR KD cells would be advantageous.
8) The question remains the same with data presented in Figure 5 regarding the difference in the state of cell polarization between sample groups. Additionally, cell monolayers with compromised CFTR expression (CF primary airway epithelial cells or CF cell models) can develop transepithelial electrical resistance similar to that of WT-expressing counterparts, demonstrating a well-polarized state in vitro. (Also see Supplemental table 1, TEER date on CF and non-CF primary epithelial airway cells) This fact indicates a similar ZO-1 organization in well-polarized CF and non-CF monolayers.
9) Why was NECA not used to treat CFTR KD cells in the experiments presented in Figure 6c? In a similar manner, why was AMP-CP not used to treat control samples in experiments depicted in Figure 6d? These additional experiments may add to our understanding of pathological mechanisms.

Reviewer #3

(Remarks to the Author)
The author's work is very interesting. The potentially describe an important aspect of airway epithelial biology that is relevant to disease. The findings are novel. The major drawback is that the majority of the work is conducted in Calu3 cell line. I feel the authors may be conflating the findings from this cancer cell line with primary airway epithelial cells. There may be some similarities, but the authors need to validate the major findings in primary CF epithelial cells. As the authors already have access to multiple cell lines (Table 1, Figure 1, Figure 8) this is something that they should do for publication.

1) I would ensure that whenever the authors refer to CF HAEC this should be only when referring to primary cells and not cell lines. There are a number of places where this is found (see 2).

2) Figure 2: On line 170-171 the authors state that "Vav3 and Cx43 are in close proimxity in the R, which may facilitate Cx43 anterograde transport toward the Golgi and trafficking of Cx43 HCs to the plasma membrane in *CF HAECs*". The experiments were conducted in Calu3 cell line with CFTR knockout. This cell line is derived from an adenocarcinoma and has many different properties than primary cells. For this statement to be correct, I would suggest that the authors do a similar proximity ligation assay in primary CF airway epithelial cells (which they have access to as seen in Fig 1 and 8).

3) Figure 3: Would be helpful to validate the ER and Golgi staining in primary CF airway epithelial cells as this is a critical finding.

4) It would be important to test the effect of HEMT on abnormal ER localization and Cx43 expression. I know the authors have previously demonstrated the lack of effect on fibronectin after 72h of treatment. A timecourse of when these abnormalities resolve or if they do not would be very insightful. Based on the Calu3 data it seems that CFTR is somehow relevant for this dysfunction. Assessment of the rescue with modulators would be very applicable here.

Other major comments:

- A phallodin stain would have been helpful to see the cell boundaries and localize where the Cx43 is found throughout the studies. At homeostasis and particularly after wounding this would have been extremely informative information to have. One would be able to visualize the leading edge of cell migration here. Providing cell boundaries on the data for which you are able would be very helpful.

- Figure 1: There is quite a bit of heterogeneity in the Cx43 expression on the CF ALI. Does this correspond to different cell types? Staining would be great to validate on your primary ALI with Cx43 staining with markers of basal, ciliated, secretory, and rare cells if able. Alternatively, you can look at the existing scRNAseq data from CF and non-CF epithelia to see the cell type specific distribution. However, I do worry about the scRNAseq data as it seems mRNA levels do not correspond to protein levels based on your S1A and 1B-D.

- Validation of the Cx43 KD and CFTR KD would be imperative to include.

- Interesting that the neurobiotin uptake is completely reduced back to baseline with the Cx43 KD. Does this imply that none of the other connexins function in Calu3 cells? Can you validate that the Cx43 KD is specific for Cx43 and the others are not affected? Do Calu3 cells have different connexins compared to primary airway epithelial cells? These differences could heavily influence the implications of the work.

- Including a summary diagram of the proposed mechanism would be nice.

- It seems that the critical piece here is that Cx43 facilitates adenosine transport that leads to fibronectin secretion. Is there a

way to demonstrate that loss of Cx43 or blocking the hemichannels effects adenosine levels?

Minor suggestions:

- The reasoning for the following 2 comments is that it establishes that Cx43 is different than the other connexins look at. Though it is not completely clear based on gene expression alone from the data provided. Does not diminish the story but would be helpful to clarify:

1A: Should probably make this color-blind friendly. Little strange color scheme. Can you make this a gradient? Black makes it seem there is no expression while that is still elevated over baseline.
Supplemental 1A: Can you include the mean levels with statistics? The claim is that GJA1 is different than the others in that gene expression remained elevated in CF. It seems that compared to uninjured, GJB2, GJB3, GJB6 are all elevated in CF. Or perhaps some clarification that this is in relation to the wounded condition. Or is the differential increase in CF much greater than compared to the differential increase in non-CF? This needs clarification.

Other minor comments:
- Figure 4 would benefit from a diagram to explain what you mean by forward, backward, lateral or none in terms of staining overlap.

- Figure 5B-C and 5E-F: Are Cx43 KD in CTL and CFTR KD similar or different? Additional statistics would be helpful here.

- More details on how the primary airway epithelial cells were grown would be helpful. How long were they differentiated at Air liquid interface before the studies?

Version 1:

Reviewer comments:

Reviewer #3

(Remarks to the Author)
The reviewers addressed the major concerns for this reviewer. I would be sure that all data (including box and whisker plots) display all the data points as per Nature Journal policy.

Open Access This Peer Review File is licensed under a Creative Commons Attribution 4.0 International License, which permits use, sharing, adaptation, distribution and reproduction in any medium or format, as long as you give appropriate credit to the original author(s) and the source, provide a link to the Creative Commons license, and indicate if changes were made.
In cases where reviewers are anonymous, credit should be given to 'Anonymous Referee' and the source.
The images or other third party material in this Peer Review File are included in the article’s Creative Commons license,

unless indicated otherwise in a credit line to the material. If material is not included in the article's Creative Commons license and your intended use is not permitted by statutory regulation or exceeds the permitted use, you will need to obtain permission directly from the copyright holder.
To view a copy of this license, visit https://creativecommons.org/licenses/by/4.0/

Answers to the Reviewers

Answers to the Reviewers' comments are in **bold**. Changes in the revised manuscript are highlighted in yellow in the "Marked Up" version.

Reviewer #1:

This is a very elegant and comprehensive study. The results are convincing. However, the reviewer has some concerns, mainly related to the presentation of the results and the methodology used for the quantifications.

We thank the Reviewer for her/his comments on our study. As indicated below, we have now improved the presentation of the results and the methodology used for quantifications.

1) For each presentation of immunofluorescence labeling with confocal microscope acquisition, it would be useful to present orthogonal projections of Z-stack images rather than (or in addition to) 3D projections in order to truly appreciate the location of the labels.

We agree that orthogonal views are important when assessing the spatial localization of signals. Accordingly, we have included orthogonal projections (new Supplementary Figure 1c and Supplementary Figure 7) or representative images from different focal planes (new Supplementary Figure 1b, Supplementary Figure 2a and Supplementary Figure 6a) when z-axis localization is essential, which has led to changes in the text of the Results section (p.6, l.127-130, p.11, l.270-271, p.12, l.292). We have also specified in the revised figure legends whether images correspond to a single confocal plane or 3D projection of z-stacks.

2) The reviewer does not understand the quantification of markers (Cx43, ZO-1, CD73, PAO1 adhesion) in relation to DAPI. In general, the various quantifications using ImageJ must be clearly and precisely explained. It is unclear what each point corresponds to in the graphs: for example, in Figure 1d, it is specified that cultures from 3 CF donors and 3 non-CF donors were analyzed, and a value of n=11-14 is given. What does this value of n correspond to? Is it the number of culture wells? The number of images analyzed? If so, how many different wells/images per patient? It is further specified that patient characteristics are given in Supplementary Table 1, which presents 5 non-CF subjects and 5 CF subjects. However, Figure S1 presents results obtained with 6 non-CF donors and 7 CF donors

We thank the reviewer for pointing this out. For each image, we measured the *Integrated Density* of the marker channel (Cx43, ZO-1, CD73 or PAO1) and of the corresponding DAPI channel. With this approach, the intensity of the markers' fluorescence is expressed as a function of nucleus number per

image. This normalization is used to ensure that measurements reflect true biological differences rather than differences in cell density. The quantification procedure is now clarified in the Methods section (p.24, l.614-621).

In Figure 1d and 1e, each value corresponds to one quantified image. The reported *n*=11–14 therefore represents the total number of images analyzed across the 3 donors in each group (CF and NCF). Typically, this corresponds to 3–5 images per donor acquired under the same experimental condition, as indicated by the color code (one color per donor). The number of images per donor depends on the number of available Transwells® that could be successfully wounded and processed.

Regarding the primary cultures obtained from 7 NCF donors and 6 CF patients, Figure 1 and Supplementary Figure 1 were generated from a previously published transcriptomic dataset from our laboratory, which was cited in the manuscript (reference 30). This is now clearly indicated in the Results section (p.6, l.114-115) and the Methods section (p.22, l.550-551). We have also implemented the characteristics of these patients in a new Supplementary Table 1 (the original supplementary Table 1 becoming Supplementary Table 2 in the revised manuscript), as indicated in the Methods section (p.21, l.513-518).

3) The heat map in Figure 1 should show data for each patient with and without cystic fibrosis, and the graphs in Figure S1 would be easier to understand if they showed average values for each time point (rather than values for each patient), with a statistical analysis.

We thank the reviewer for this helpful suggestion. The heatmap in Figure 1 has been revised accordingly so that it now displays the data for each individual NCF and CF donor.

Supplementary Figure 1a has been updated to present the mean expression levels at each time point, normalized to the corresponding non-wounded condition for each donor. The figure also includes the appropriate statistical analysis (Two-way ANOVA, Tukey's multiple comparisons test). This has led to changes in legends of Figure 1a and Supplementary Figure 1a.

4) At what level relative to the wound were the photos taken in Figure 1? Was it in the previously damaged area, or behind it? This needs to be clarified.

At times of wound closure (WC) and 2-days post WC (pWC), the photos shown in Figure 1a were taken at the area that was damaged and subsequently repopulated during epithelial repair. This is now clarified in the revised manuscript (p.6, l.121-123).

5) There appears to be an error in Figure 1m, which shows CD73 expression instead of CX43 expression.

We thank the reviewer for noting this error. Figure 1m has been corrected accordingly.

6) Regarding the polarization of Calu-3 cell cultures, this is achieved when the cells are confluent and then placed at the air-liquid interface. In this case, how do the authors explain the fact that, in the photos, there are many areas that do not show cells (figures 1l, 2d, 2f, 2h, 3c, 7i, S2, S3)?

We apologize for the lack of clarity in the description of these images. At the air–liquid interface (ALI), Calu-3 cells progressively change morphology from a flat, confluent monolayer to a taller, pseudostratified epithelium. As a result, not all cells are present within the same focal plane when images are acquired at a single z-level. Consequently, regions that appear devoid of nuclei in the images do not reflect an absence of cells, but rather areas where nuclei/cells are positioned outside the focal plane. This is corroborated by the absence of "empty areas" in 3D reconstructions of the full epithelium (Figure 5i and Figure 6f). This precision is now systematically provided in legends of Figures showing single focal plane images (Figure 1i and l; Figure 2d, 2f and 2h; Figure 5i; Supplementary Figure 1b; Supplementary Figure 2a; Supplementary Figure 6a), or 3D projections with a focus on the apical epithelial surface in Figure 3c and Figure 7i (now Figure 7e).

7) Page 7 line 162-163: how to determine that the colocalization is mostly observed in cytoplasmic compartment rather than at the cell membrane with orthogonal projections of Z-stack images?

The sentence in line 162-163 has been deleted. Evidence that the close Vav3/Cx43 proximity in CFTR KD cells mostly occurs in the cytoplasm comes from PLA experiments combined with ER marker staining (Figure 2f-h). The text has been modified accordingly (p.8, l.173-175).

8) Figure 3: it is difficult to see a "perinuclear" organization of the ER and a fragmentation of the Golgi. When looking at the images, what allows the author to claim that the Golgi apparatus is fragmented?

We thank the reviewer for this important remark. In the original orthogonal projection shown in Figure 3c (CTL), the displayed plane was not aligned with the nuclei, which made the perinuclear ER organization less apparent. We have now adjusted the z-plane for the same image to better show nuclei surrounded by ER staining. This adjustment, which demonstrates the perinuclear localization of the ER in CTL cells, has led to revision of Figure 3 (new Figure 3b).

We agree that immunofluorescence staining with the Golgi marker giantin only showed a dispersed punctate pattern in CFTR KD cells compared with the compact perinuclear staining in CTL cells. To provide additional and more direct evidence of the Golgi ultrastructure in CFTR KD cells, we performed transmission electron microscopy (TEM) experiments. As shown in panel d of the new Figure 3, TEM images clearly show the breakdown of the typical stacked cisternal organization of the Golgi into smaller, dispersed elements, corroborating the immunofluorescence observations and

consistent with Golgi dispersal at the ultrastructural level. We have therefore revised the manuscript to include this TEM data and adapted Figure 3d legend, the Results section (p.9, l.201-206) and the Methods section (p.25-26, l.643-659), accordingly.

9) Figure 4: The reviewer does not understand how the orientation of the organelles is determined. In relation to what? We assume that the orientation of the nucleus depends on the position of the cell in relation to the wound... but how many rows of cells border the wound? How is this standardized so that conditions can be compared? How can orientation be expressed as a percentage? What do the points in the graphs correspond to?

We thank the reviewer for this comment and the opportunity to improve Figure 4. The orientation of the Golgi and centrosome was determined in relation to the nucleus and the wound edge. Only cells in the first row bordering the wound (leading-edge cells) were analyzed to ensure consistency across samples. Golgi (giantin) and centrosome (pericentrin) fluorescent signals were classified based on their positioning relative to the nucleus: forward (toward the wound), backward (away from the wound), lateral (perpendicular to the wound edge), or none when Golgi staining overlapped with the nucleus. For each image, the percentage of cells displaying each orientation was calculated relative to the total number of leading-edge cells. Each value in the graphs corresponds to a single analyzed image, allowing reproducible comparisons across experimental conditions. This is now mentioned in the Results section (p.9, l.227-229) and described in the Methods section (p.26-27, l.672-682) of the revised manuscript.

To standardize these measurements, we implemented a custom automated ImageJ macro, with minimal manual input limited to tracing the wound edge. This approach, which measured the orientation angle for Golgi and centrosome relative to the wound edge is now detailed in the Methods section of the revised manuscript (p.27, l.687-706) and in the new Supplementary Figure 4b-d. The text of the Results section has been modified accordingly (p.10, l.234-240).

Does Cx43 depletion alter the speed or orientation of cell migration? It would be interesting to add the statistical value of the Scr and Cx43 KD comparisons in the CTL condition.

A detailed analysis of migration speed and directionality at the single-cell level would require individual cell tracking. This is not technically feasible in Calu-3 cells, which migrate as cohesive colonies. We did, however, perform wound healing assay in CFTR KD and Cx43/CFTR KD monolayers. Wound closure was normalized to the gap measured 1 h post-wounding. As shown in a new Supplementary Figure 4f, both conditions reached wound closure at 24 h with no significant difference, indicating that overall cell migratory capacity is not affected by Cx43 depletion. The

wound healing assay and its outcome have led to changes in the Results (p.10, l.252-254) and Methods (p.26, l.660-666) sections of the revised manuscript.
The statistical comparison between Scr and Cx43 KD conditions in CTL cells has now been included in Figures 4b and 4d, as requested. No significant differences were observed.

10) Figure 5: how was determined the Golgi fragments counts (5b)? It would be interesting to add the statistical value of the Scr and Cx43 KD comparisons of TEER values in the CTL condition
The method of quantification has been expanded in the revised Methods section (p.27-28, l.707-715). In brief, Golgi dispersal was quantified using ImageJ by threshold-based segmentation of the Golgi marker fluorescence followed by particle analysis. Raw images were converted to 8-bit grayscale images that were subjected to an identical thresholding method and parameters to generate binary masks of Golgi structures. Segmentation was based on pixel connectivity, allowing separation of continuous Golgi structures into discrete elements. The "Analyze Particles" function was then applied to identify each discrete particle as an individual Golgi signal cluster. The total number of particles per area was referred to as Golgi signal counts. Identical acquisition and analysis parameters were applied to all samples.
To improve visualization, Golgi staining is now presented without DAPI in the Figure 5a, like the ZO-1 images in panel d. The corresponding orthogonal views, including DAPI staining for both Golgi and ZO-1, are provided in the new Supplementary Figure 7a and b.
The statistical value of the Scr and Cx43 KD comparisons of TEER is now given in Figure 5f, as requested.

11) Figure 6: how is standardized the quantification of positive cells between conditions (fig 6l)?
The dye injection method, which is routine in the laboratory, is now clearly described in the revised manuscript (p.30, l.777-778 and l.784-788). To ensure standard conditions and reliable quantification across experiments, each dye injection was done for 3 min and up to 5 injections were made per Transwell® within 15 min before fixation of the cells. Quantification of dye coupling was performed by counting the number of neurobiotin-positive cells per injection site, using identical acquisition parameters and a consistent intensity threshold across all conditions. Only cells exhibiting a signal clearly above background and spatially connected to the injected cell were included, in line with standard practices. No difference in the extent of dye coupling was observed between the first and the last injections made within the 15 min time window.

12) Figure 8: Do the values shown in graph 8d correspond to the quantification of the Western blot experiment in the same figure (8b)? Because it is specified in the caption that the Western blot was performed with cultures from two donors, and there are three donors in the graph.

The values shown in Figure 8d correspond to the quantification of the Western blot experiments presented in Figure 8b (now Figure 8c) and of an additional Western blot from a third CF donor. Figure 8b (now Figure 8c) shows representative Western blot images from 2 donors that include all experimental conditions tested (TAT-Cx43, αCT-1, and AMP-CP), thereby providing a complete visual representation of the treatments. We have revised the legend of Figure 8 to clarify this point and avoid potential confusion.

Page 8, line 170: The reviewer does not understand the "anterograde" notion in this case

This is indeed an assumption. We thus deleted the sentence from the Results section of the revised manuscript.

Reviewer #2:

The manuscript will be of great interest to the field of cystic fibrosis. Statistical analysis used in the described studies is adequate.

We thank the Reviewer for her/his comments on our manuscript.

Questions and comments:

1) The efficiency of CFTR KD in Calu-3 cells would be desirable to show on protein levels (Western blots) and whether Cx43 KD has any effect on the CFTR KD efficiency (Figure 5).

The efficiency of CFTR KD in Calu-3 cells has been reported previously (references 19 and 31). To validate CFTR knockdown efficiency, we measured CFTR short-circuit current in Ussing chambers to provide a functional readout of CFTR-dependent transepithelial ion transport. This approach is widely considered as a sensitive and physiologically relevant method to evaluate CFTR activity, and it reflects both protein expression and channel function at the epithelial surface. Using this method, we confirmed efficient CFTR knockdown in Calu-3 cells. Importantly, CFTR-dependent currents were not altered by Cx43 knockdown, indicating that Cx43 KD does not alter CFTR KD efficiency. These new data have been included in the revised manuscript (p.9, l.212-216, p.28-29, l.731-744) and in new Supplementary Figure 3c and d.

2) It would be preferable to demonstrate a nearly equivalent level of HuR protein expression in both CFTR KD and control Calu-3 cells to support the findings in Figures 2b and 2c. This would provide evidence that manipulating CFTR expression does not selectively or nonspecifically increase HuR protein levels.

The point here is that manipulating CFTR expression does increase HuR protein levels. Our previous work demonstrated that HuR protein is intrinsically upregulated in primary CF HAECs and that CFTR depletion in Calu-3 cells recapitulated this phenotype (reference 24). Thus, achieving equivalent HuR protein levels between CFTR KD and CTL Calu-3 cells is not feasible within this biological context.

The data presented in Figures 2b and 2c should be interpreted considering this established difference, where increased Cx43 mRNA stability in CFTR-deficient cells is due to elevated HuR expression. We have rewritten the Results section to better explain the causal relationship between HuR expression and Cx43 mRNA stability in the CF context (p.7, l.152-154).

3) The author states that cells shown in Figure 2d, 2f, and 2h are polarized. To claim full polarization of airway epithelial cells, the cells should appear as continuous monolayers on Transwell support, not patchy structures like those shown in Figure 2d, 2f, and 2h. Holes in monolayers, especially those demonstrated in Figure 2f on the CFTR KD immunofluorescent image, illustrate a growing non-polarized state. If cells were grown on coverslips, full polarization of cells cannot be claimed.

The polarized Calu-3 cells shown in Figures 2d, 2f, and 2h were grown exclusively on Transwell® inserts and not on coverslips. Briefly, Calu-3 cells are seeded onto the apical side of the Transwell® membrane. At this stage, both apical and basolateral compartments contain culture medium. Once a tight confluent monolayer is reached, the medium is removed from the apical side to create an air-liquid interface (ALI), mimicking the native condition of an airway epithelium. This is now clarified in the Results (p.6, l.137-138) and Methods (p.21, l. 526-527) sections of the revised manuscript.

With time of polarization at ALI, the cells progressively change morphology from a flat monolayer to a taller, pseudostratified epithelium. As a result, not all cells are present within the same focal plane when images are acquired at a single z-level. Regions that appear as "holes" do not reflect an absence of cells but rather areas where nuclei are positioned outside of the focal plane. These patchy structures are not observed in 3D reconstructions of the full epithelium (Figure 5i and Figure 6f). This information is now systematically provided in the legend of Figures showing single z-level images (Figure 1i and l; Figure 2d, 2f and 2h; Figure 5i; Supplementary Figure 1b; Supplementary Figure 2a; Supplementary Figure 6a), or 3D projections with a focus on the apical epithelial surface in Figure 3c and Figure 7i (now Figure 7e).

4) The author states that CFTR KD cells demonstrate enhanced intracellular Vav3/Cx43 proximity compared to control cells, as shown in Figure 2f and g. Is it possible that the CFTR KD cells grow more slowly and do not reach full polarization by the time of ALI D14? In my opinion, measuring comparable transepithelial electrical resistance between control and CFTR KD ALI monolayers—indicating full polarization regardless of the time on ALI—would provide a more accurate assessment of differences in Cx43-Vav3 co-localization and enhancement, if cells were grown on Transwells, not on coverslips.

We thank the reviewer for this insightful comment. We agree that difference in growth between CTL and CFTR KD cells may affect their polarity. However, tight monolayers of Calu-3 cells decreased their proliferation when switched at ALI, as previously evaluated using the Ki67 marker (reference 23). In fact, D14 is chosen to compare CTL and CFTR KD cells because proliferation is nil at ALI D14 in both cell lines (reference 23). This is now clarified in the revised manuscript (p.6-7, l.139-140). It is also important to state that alteration of junctional complexes is a phenotype of CF HAECs that is recapitulated in CFTR depleted Calu-3 cells. With polarization at ALI, CFTR KD Calu-3 cells

reproducibly develop lower TEER values compared to CTL cells at later time points (e.g., D14 in Figure 5f), consistent with our previous report (reference 21). This reduction in TEER is a well-established consequence of CFTR deficiency and reflects the widely reported intrinsic alterations in epithelial barrier integrity (reference 63). To illustrate this point, we have added TEER measurements of primary NCF and CF HAEC cultures (p.11, l.273-274 and Supplementary Figure 6c).

We believe that our observations indicate that the enhanced intracellular Vav3/Cx43 proximity shown in Figures 2f and 2g is unlikely to result from differences in proliferation but instead reflects true biological changes in protein localization associated with CFTR deficiency.

5) Similarly, the difference between control and CFTR KD cell polarization states in experiments demonstrated by Figure 3 is questionable. Days on ALI do not determine the maximum polarization of a cell culture monolayer. Equivalent transepithelial electrical resistance would be a better method for assessing comparable cell polarization between control and CFTR KD monolayers, regardless of the days of polarization.

As mentioned above, TEER and junctional complexes organization are intrinsically altered in primary CF HAECs and in CFTR KD Calu-3 cells. The difference in polarization state is a hallmark of the CF phenotype, and it is proposed that the CF airway epithelium exhibits a partial epithelial-mesenchymal transition state. This notion is now emphasized in the revised manuscript (p.14, l.364-365, p.18, l.449-451). Figure 3 shows that persistent Cx43 expression is associated with polarity defect of CFTR KD cells.

6) The author states that giantin immunostaining shows increased Golgi fragmentation and dispersal in CFTR KD cells polarized at ALI (Figure 3d). Actively dividing cells also display a dispersed Golgi structure, underscoring the importance of imaging control and CFTR KD cell monolayers in a similarly well-polarized state.

As explained above, Calu-3 cells are not actively dividing at D14 of ALI. Therefore, the Golgi dispersal observed in CFTR KD cells by giantin immunostaining reflects a CFTR-dependent defect in epithelial organization.

7) An increase in microtubule branching is also a sign of dividing cells during mitotic spindle formation. The literature shows that loss of Cx43 triggers cells to enter the cell cycle and enhances cancer cell invasion. As I mentioned previously, CFTR KD cell growth or cell duplication may be compromised (slowed) by a non-specific mechanism (a non-specific effect of KD), resulting in decreased cell duplication and mitotic

spindle formation. Additionally, Calu-3 cells are cancer cells with an altered growth rate, and Cx43 KD may not further increase their growth rate. (Figure 4.f) With CFTR KD, cell growth and microtubule branching may decrease, a reversal that can be achieved with Cx43 KD. It would be beneficial to repeat this experiment with non-cancerous, CFTR-expressing epithelial cells, such as 16HBE or primary cells, if possible. Furthermore, demonstrating similar cell duplication times between control and CFTR KD cells would be advantageous.

We agree with the Reviewer that increased microtubule branching can be associated with mitotic spindle formation in actively dividing cells, and that proliferation-related effects should be carefully controlled in this context. As correctly pointed out, experiments shown in Figure 4f were performed on cells grown on coverslips, which are in a proliferative state. To address this concern, we monitored cell growth using an MTT assay in Calu-3 cells during their active proliferation phase (p.26, l.667-671). As shown in the new Supplemental Figure 4e, we observed comparable growth rates between CTL and CFTR KD cells. Importantly, proliferation remained similar upon Cx43 knockdown in both genotypes, as mentioned in the Results section of the revised manuscript (p.10, l.249-252).

We acknowledge the Reviewer's request to validate the findings in primary cells. We were able to measure Golgi/centrosome orientation and microtubule branching in one primary HAEC CF and NCF culture, and the outcome reproduced the phenotypes observed in Calu-3 cell models. These new results have been added to the revised manuscript, leading to changes in the Results section (p10, l.254-257) and in Supplementary Figures 5 and 6. Although the sample size precludes formal statistical analysis, this result supports that the changes in microtubule organization are not attributable to altered cell proliferation or intrinsic properties of cancer-derived cells but rather reflect a CFTR-dependent phenotype

8) The question remains the same with data presented in Figure 5 regarding the difference in the state of cell polarization between sample groups. Additionally, cell monolayers with compromised CFTR expression (CF primary airway epithelial cells or CF cell models) can develop transepithelial electrical resistance similar to that of WT-expressing counterparts, demonstrating a well-polarized state in vitro. (Also see Supplemental table 1, TEER date on CF and non-CF primary epithelial airway cells) This fact indicates a similar ZO-1 organization in well-polarized CF and non-CF monolayers.

Indeed, CFTR KD cells and primary CF HAECs develop TEER. Primary CF HAECs also differentiates into a mucociliated airway epithelium. Nevertheless, the CF airway epithelium exhibits barrier integrity defects and disorganization/mislocalization of epithelial markers, including ZO-1, which are hallmarks of the disease (reference 28). We systematically measure TEER of primary HAECs upon receipt. We confirmed a significant decrease in the TEER of CF primary HAECs

compared to NCF controls (8 NCF donors and 8 CF patients). These results are now shown in Supplementary Figure 6c of the revised manuscript.

TEER is highly dependent on culture conditions and differentiation state, thus direct comparison with independently generated datasets should be made with caution. Please note that the TEER values reported in Supplemental Table 1 (now Supplementary Table 2) are provided by the manufacturer from the different batches of production, for which detailed experimental conditions are not available. These given values do not correspond to the Transwells® that were used for this study.

9) Why was NECA not used to treat CFTR KD cells in the experiments presented in Figure 6c? In a similar manner, why was AMP-CP not used to treat control samples in experiments depicted in Figure 6d? These additional experiments may add to our understanding of pathological mechanisms.

AMP-CP was not applied to control cells because of the low expression level of CD73, and inhibition would therefore be unlikely to produce a detectable effect. Conversely, NECA was not used on CFTR KD cells because CD73 expression is elevated in these cells, resulting in a near-maximal NECA-mediated response; additional treatment was not expected to further enhance the effect. We nevertheless performed the suggested experiments, which are now shown in a new Supplementary Figure 8a and b. No significant differences in fibronectin expression were observed, confirming that the original experimental design appropriately captures the relevant CD73/adenosine signaling effects. These new results have led to changes in the Results (p.12, l.297-299) and Discussion (p.18, l.458-459) sections of the revised manuscript.

Reviewer #3:

The author's work is very interesting. The potentially describe an important aspect of airway epithelial biology that is relevant to disease. The findings are novel. The major drawback is that the majority of the work is conducted in Calu3 cell line. I feel the authors may be conflating the findings from this cancer cell line with primary airway epithelial cells. There may be some similarities, but the authors need to validate the major findings in primary CF epithelial cells. As the authors already have access to multiple cell lines (Table 1, Figure 1, Figure 8) this is something that they should do for publication.

We thank the Reviewer for her/his positive and pertinent comments. Please note that because of the efficiency of highly effective modulator therapy, airway epithelial cells cultures from CF patients are exceptionally difficult to obtain from our provider currently.

1) I would ensure that whenever the authors refer to CF HAEC this should be only when referring to primary cells and not cell lines. There are a number of places where this is found (see 2).

We went carefully through the manuscript to clearly refer to primary CF HAECs or CFTR KD Calu-3 cells, as underlined in the Marked Up revised version.

2) Figure 2: On line 170-171 the authors state that "Vav3 and Cx43 are in close proximity in the ER, which may facilitate Cx43 anterograde transport toward the Golgi and trafficking of Cx43 HCs to the plasma membrane in *CF HAECs*". The experiments were conducted in Calu3 cell line with CFTR knockout. This cell line is derived from an adenocarcinoma and has many different properties than primary cells. For this statement to be correct, I would suggest that the authors do a similar proximity ligation assay in primary CF airway epithelial cells (which they have access to as seen in Fig 1 and 8).

We thank the reviewer for this valuable suggestion. Unfortunately, Duolink proximity ligation assays could not be performed in primary cells under our experimental conditions, as Cx43 detection relied on OPAL tyramide signal amplification, which is not compatible with Duolink methodology. To address this limitation, we performed co-immunostaining for Vav3 following Cx43 OPAL staining on sections from CF and non-CF primary HAECs. Pearson's correlation analysis revealed increased colocalization of Vav3 and Cx43 in CF cells compared to NCF controls, supporting the observations obtained in Calu-3 CFTR KD cells. These new data are shown in new Supplementary Figure 2b and c (p.8, l.179-183).

3) Figure 3: Would be helpful to validate the ER and Golgi staining in primary CF airway epithelial cells as this is a critical finding.

We agree that validation in primary CF HAECs would strengthen the relevance of our findings. ER validation in primary cells was not feasible, as the ER probe requires live-cell incubation, which could not be applied to the limited number of CF primary HAEC cultures available at this time.
However, we were able to determine Golgi orientation and Golgi dispersal in one primary CF HAEC culture. Golgi/centrosome misalignment, microtubule branching defect and dispersed Golgi phenotype were observed as in the CFTR KD cell line model. While single sample does not allow statistical generalization, this result provides biological confirmation that the secretory axis is also altered in primary CF HAECs. These new results are now provided in the revised manuscript (p.10, l.254-257), leading to new Supplementary Figures 5 and 6.

4) It would be important to test the effect of HEMT on abnormal ER localization and Cx43 expression. I know the authors have previously demonstrated the lack of effect on fibronectin after 72h of treatment. A time course of when these abnormalities resolve or if they do not would be very insightful. Based on the Calu3 data it seems that CFTR is somehow relevant for this dysfunction. Assessment of the rescue with modulators would be very applicable here.
We thank the reviewer for this important suggestion and agree that a 72 h treatment period may be too short to observe effects on connexin expression. While detailed investigation of connexin regulation by ETI therapy is beyond the scope of the current study, we examined available RNA-seq datasets from the literature. In a recent 2025 study, nasal brushings of the inferior turbinate were collected from 30 participants (28, 24, and 23 participants at baseline (BL), 6 months, (6 mo.) and 2 years (2yr.) post-ETI therapy, respectively (reference 85). Analysis of these datasets indicates that expression of Cx43 (*GJA1*) was not reduced by ETI treatment, even two years post-therapy. This observation suggests that Cx43 dysregulation may persist despite longer ETI therapy; however, these findings should be confirmed at the protein level and warrant further investigation in future studies. This information is now provided in the Discussion of the revised manuscript (p.19, l.505-507).

BL vs 6 mo.			BL vs. 2 yr.			6 mo. vs. 2 yr.		
Gene	log2FC	adj. p-val	Gene	log2FC	adj. p-val	Gene	log2FC	adj. p-val
GJA1	0.058	0.627918	*GJA1*	-0.00193	0.855519	*GJA1*	-0.06017	0.877384

5) Other major comments:
- A phallodin stain would have been helpful to see the cell boundaries and localize where the Cx43 is found throughout the studies. At homeostasis and particularly after wounding this would have been extremely informative information to have. One would be able to visualize the leading edge of cell migration here. Providing cell boundaries on the data for which you are able would be very helpful.

In primary HAECs, Cx43 immunostaining was performed at wound closure and 48 h post-wound closure; at these points the wounded area was already completely repopulated by new cells, so the leading edge cannot be seen. To better visualize cell boundaries, we performed Cx43 immunostaining combined with phalloidin at 48 h post-wound closure, a time when the differences between NCF and CF cells are most pronounced during repair. These images, acquired using z-stack confocal imaging, reveal both membrane-associated and cytoplasmic Cx43 signals, with localization varying depending on the basal, middle, or apical focal plane. Representative images have been included in the revised manuscript (Supplementary Figure 1b).

- Figure 1: There is quite a bit of heterogeneity in the Cx43 expression on the CF ALI. Does this correspond to different cell types? Staining would be great to validate on your primary ALI with Cx43 staining with markers of basal, ciliated, secretory, and rare cells if able. Alternatively, you can look at the existing scRNAseq data from CF and non-CF epithelia to see the cell type specific distribution. However, I do worry about the scRNAseq data as it seems mRNA levels do not correspond to protein levels based on your S1A and 1B-D.

We agree that the observed heterogeneity in Cx43 expression across the CF ALI epithelium may reflect differences between cell types. While detailed co-staining for basal, ciliated, secretory, and rare cell markers in primary ALI cultures is beyond the scope of the current study, we bring to the Reviewer's attention that a single-cell multiomic study is currently ongoing in our laboratory, which will specifically address *GJA1* expression across these epithelial cell types in CF and NCF epithelia. Although we cannot publish these data at this time, we can already share that *GJA1* is expressed in all cell types, except Tuft cells and Ionocytes.

- Validation of the Cx43 KD and CFTR KD would be imperative to include.

Validation of the Cx43 knockdown is already shown in Supplementary Figure 3b. For CFTR knockdown, as also requested by Reviewer 2, we performed electrophysiology experiments in Ussing chambers, which directly measure CFTR-dependent transepithelial ion transport and provide an informative readout of CFTR protein function. These experiments confirmed robust CFTR knockdown in Calu-3 cells and showed that Cx43 knockdown did not affect CFTR silencing. These data have now been included in the revised manuscript (p.9, l.212-216, p.28-29, l.731-744) and in new Supplementary Figure 3c and d.

- Interesting that the neurobiotin uptake is completely reduced back to baseline with the Cx43 KD. Does this imply that none of the other connexins function in Calu3 cells? Can you validate that the Cx43 KD is

specific for Cx43 and the others are not affected? Do Calu3 cells have different connexins compared to primary airway epithelial cells? These differences could heavily influence the implications of the work.

We thank the reviewer for this important point. To assess specificity of the Cx43 knockdown and potential contributions of other connexins, we performed qPCR analysis in Calu-3 cells for *GJB2*, *GJB3* and *GJB6*, the connexin mRNAs measured in primary HAECs. Whereas *GJB3* and *GJB6* transcripts were not detected, *GJB2* expression was at the limit of detection. None of them showed compensatory upregulation in the Cx43/CFTR KD cells. This information is now provided in the Results section of the revised manuscript (p.9, l.216-219 and Supplementary Figure 3e) and confirms that intercellular communication in Calu-3 cells is specifically dependent on Cx43.

- Including a summary diagram of the proposed mechanism would be nice.

We have included a summary diagram in the revised version of the manuscript (Figure 8e), as suggested by the Reviewer (p.14, l.373-374).

- It seems that the critical piece here is that Cx43 facilitates adenosine transport that leads to fibronectin secretion. Is there a way to demonstrate that loss of Cx43 or blocking the hemichannels effects adenosine levels?

We thank the reviewer for this suggestion. To directly test whether Cx43 hemichannels regulate adenosine levels, we measured extracellular adenosine using the MET-5090 kit (Cell Biolabs, p.29-30, l.764-771). As expected, using calcium-free solution to open hemichannels led to a robust increase in adenosine concentration (≈20µM) in the apical secretome of CFTR KD cells 3 h post-incubation. Importantly, this increase was inhibited by αGA pretreatment, strengthening the specific role of Cx43 hemichannels in facilitating adenosine release. These new results are now shown in Supplementary Figure 8f (p.13, l.323-327, p.18, l.468-469).

6) Minor suggestions:

- The reasoning for the following 2 comments is that it establishes that Cx43 is different than the other connexins look at. Though it is not completely clear based on gene expression alone from the data provided. Does not diminish the story but would be helpful to clarify:

1A: Should probably make this color-blind friendly. Little strange color scheme. Can you make this a gradient? Black makes it seem there is no expression while that is still elevated over baseline. Supplemental 1A: Can you include the mean levels with statistics? The claim is that GJA1 is different than the others in that gene expression remained elevated in CF. It seems that compared to uninjured, GJB2, GJB3, GJB6 are all elevated in CF. Or perhaps some clarification that this is in relation to the wounded

condition. Or is the differential increase in CF much greater than compared to the differential increase in non-CF? This needs clarification.

Figure 1a has been completely revised following this comment and those of Reviewer 1. The heatmap now displays data for each individual patient (NCF and CF) using a color gradient, as recommended, to improve clarity and accessibility.

Following both Reviewers' comments, Supplementary Figure 1a has also been updated to present the mean expression levels of the most highly expressed connexin genes at each time point, normalized to the corresponding non-wounded condition for each donor. In addition, appropriate statistical analyses have now been included (two-way ANOVA with Tukey's multiple comparisons test).

This revised analysis clarifies that while *GJB2*, *GJB3*, and *GJB6* exhibit a transient increase during epithelial repair, *GJA1* (Cx43) is the only connexin gene that remains significantly elevated after wound closure in CF samples.

7) Other minor comments:

- Figure 4 would benefit from a diagram to explain what you mean by forward, backward, lateral or none in terms of staining overlap.

We thank the reviewer for this suggestion. To clarify the classification of Golgi and centrosome orientation (forward, backward, lateral, or none), we have added a diagram illustrating these categories, now shown in Supplementary Figure 4a. In addition, following a request from Reviewer 1, we included a new quantification based on precise angle measurements using a custom automated ImageJ macro (p.27, l.687-706). This approach provides a complementary, reproducible assessment of Golgi and centrosome orientation, improving the rigor and clarity of the data presented in Figure 4a-d (p.10, l.234-240).

- Figure 5B-C and 5E-F: Are Cx43 KD in CTL and CFTR KD similar or different? Additional statistics would be helpful here.

Statistics related to CTL/Cx43 KD and Cx43/CFTR KD cells are now explicitly shown in revised Figure 5. There are no significant differences.

- More details on how the primary airway epithelial cells were grown would be helpful. How long were they differentiated at Air liquid interface before the studies?

We thank the reviewer for this comment. Primary airway epithelial cell cultures (MucilAir) were obtained from Epithelix (Plan-les-Ouates, Switzerland). These cultures on Transwells® were fully differentiated at ALI for 2 months post-seeding. As Mucilair cultures were received ready for

experimentation, we do not have additional details regarding the specific differentiation protocol used by the Supplier.
