# Supplementary material for: Cx43 levels guide apicobasal polarity in regenerating airway epithelial cells
Source: Commun Biol. 2026 Jun 12;9:1264. doi: 10.1038/s42003-026-10413-7 (PMC13616896; doi:10.1038/s42003-026-10413-7)

Supplementary Information

Cx43 levels guide apicobasal polarity in regenerating airway epithelial cells

Mehdi Badaoui[1,*], Alexandre Luscher[2], Mengjie Ma[1], Marc Bacchetta[1], Christian van Delden[2], Thilo Köhler[2], Arantxa Tabernero[3], Marc Chanson[1,4,5]

[1]*Department of Cell Physiology & Metabolism, Faculty of Medicine, University of Geneva, Switzerland*

[2]*Department of Microbiology & Molecular Medicine, Faculty of Medicine, University of Geneva, Switzerland*

[3]*Department of Biochemistry and Molecular Biology, Neuroscience Institute of Castilla y León, Institute for Biomedical Research of Salamanca, University of Salamanca, Salamanca, Spain*

[4]*Department of Pediatrics, Gynecology and Obstetrics, Faculty of Medicine, University of Geneva, Switzerland*

[5]*Geneva Center for Inflammation Research (GCIR), Faculty of Medicine, University of Geneva, Switzerland*

***Corresponding author:**

Mehdi Badaoui, PhD

Department of Cell Physiology & Metabolism

Faculty of Medicine (CMU)

1, Rue Michel-Servet, 1211 Geneva (Switzerland)

Tel: +41 22 37 95 206; **Fax:** +41 22 37 95 260

Email: Mehdi.Badaoui@unige.ch

Supplementary Figures and legends

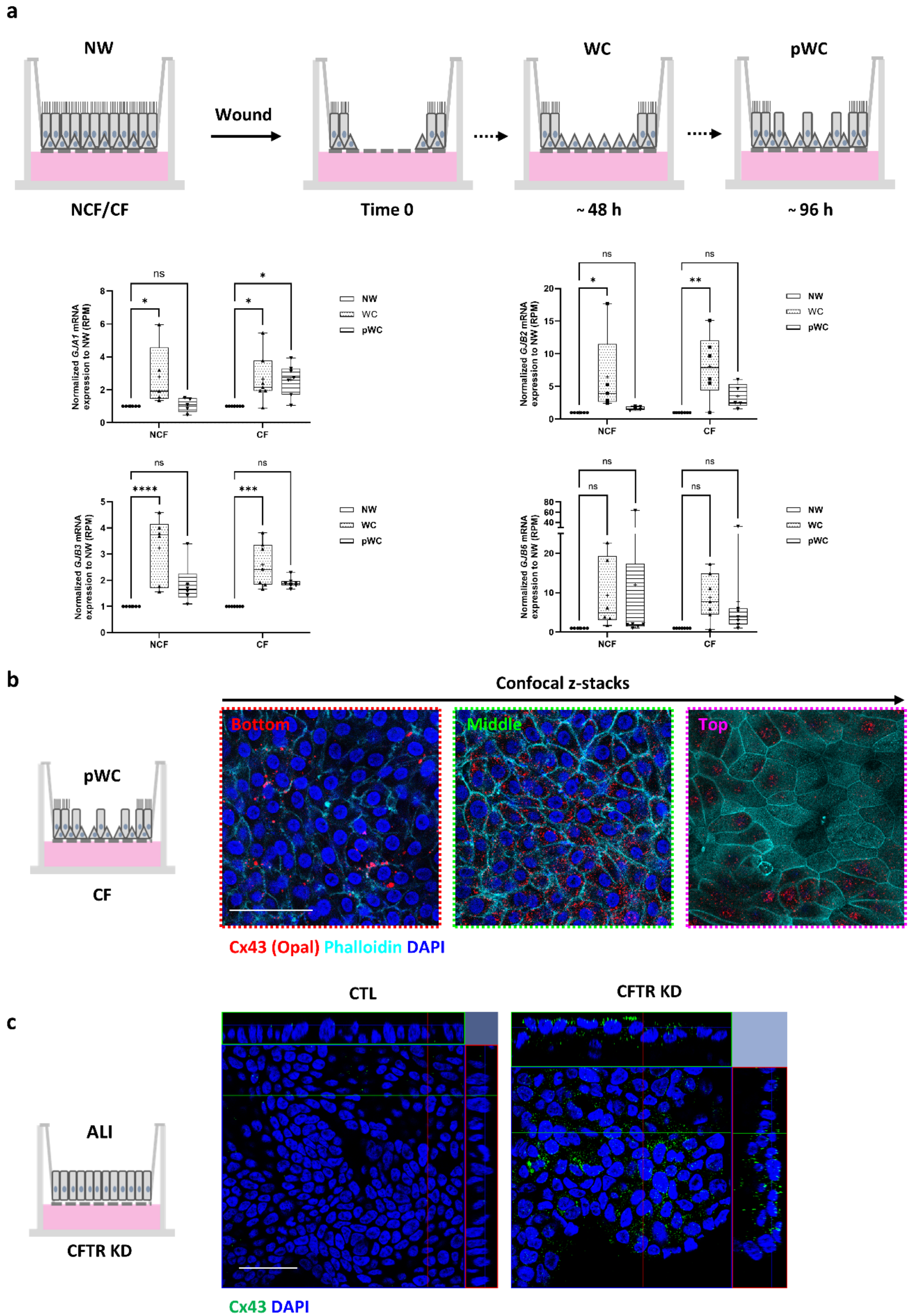

Supplementary Figure 1. Gene expression of the top detected connexin isoforms in repairing primary HAECs and Cx43 localization in polarized Calu-3 cells.

a Schematic representation of the wound repair process in fully differentiated primary HAECs generated from F508del-CFTR (CF) and non-CF (NCF) donors (Top panels). The non-wounded (NW) condition represents the intact epithelium prior to injury. At time 0, the cultures were wounded using an airbrush, leaving the peripheral epithelium untouched. After injury, activated basal cells (triangles) repopulate the wounded area through active migration and proliferation processes. Complete wound closure (WC) occurred approximately 48 h after injury while basal cells initiate the differentiation process (rectangles) 48 h post-wound closure (pWC; corresponding to 96 h post-wounding). Bottom panels show the mean expression levels at each time point of the top detected connexin isoforms (*GJA1*, *GJB2*, *GJB3*, *GJB6*) normalized to the corresponding NW condition for each donor. Data are represented as box-and-whisker plots; Two-way ANOVA with post hoc Tukey's multiple comparison test. **b** Representative single focal plane images of Cx43 OPAL™ immunostaining (red) on CF primary HAECs at pWC. z-stack imaging reveals Cx43 localization at the cell membrane and in the cytoplasm, with distinct patterns depending on the basal (red outline), middle (green outline), or apical (pink outline) focal plane. Phalloidin staining (cyan) highlights cell boundaries. Nuclei were stained with DAPI (blue). Scale bar: 50 µm. **c** Orthogonal views of z-stack confocal images (corresponding to Figure 1i) showing Cx43 immunostaining (green) in polarized CTL and CFTR KD at ALI. Nuclei are stained with DAPI (blue). The yz-views depict Cx43 distribution across the cells. Scale bar: 50 µm.

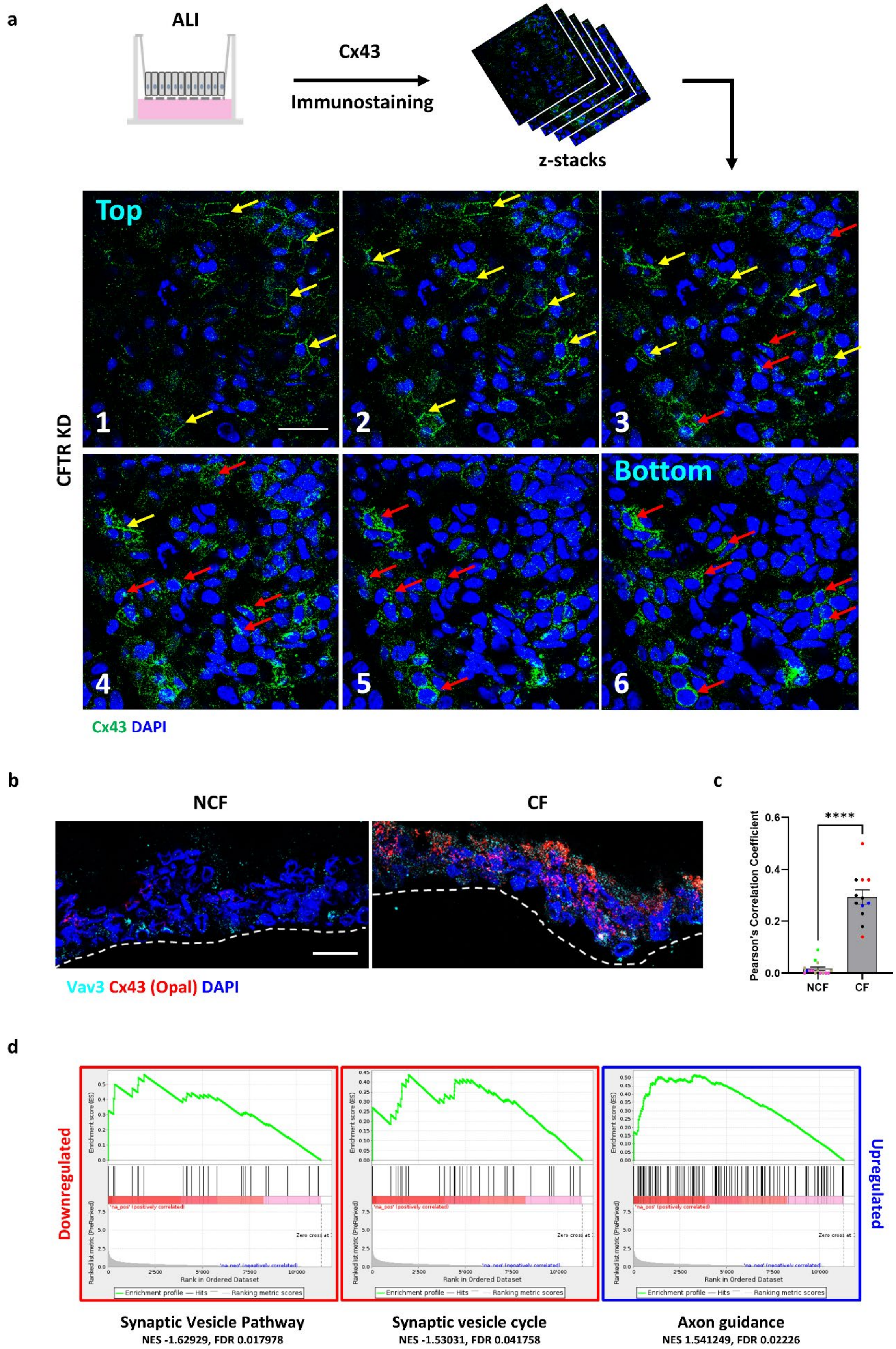
a
ALI
Cx43
Immunostaining
z-stacks
Top
Bottom
CFTR KD
1
2
3
4
5
6
Cx43 DAPI
b
NCF
CF
Vav3 Cx43 (Opal) DAPI
c
Pearson's Correlation Coefficient
****
NCF
CF
d
Downregulated
Upregulated
Synaptic Vesicle Pathway
NES -1.62929, FDR 0.017978
Synaptic vesicle cycle
NES -1.53031, FDR 0.041758
Axon guidance
NES 1.541249, FDR 0.02226

Supplementary Figure 2. Cx43 localization across the polarized CFTR KD epithelium and colocalization with Vav3 in primary HAECs.

a Representative single focal plane images of Cx43 immunostaining (green) in polarized CFTR KD Calu-3 cells throughout the epithelium, from the apical (top) to the basal region (bottom). Nuclei are stained with DAPI (blue). Scale bar: 30 µm. Cx43 is observed at cell–cell junctions (yellow arrows) as well as within intracellular compartments (red arrows), indicating its distinct spatial distribution. **b** Representative confocal images of Cx43 (red) and Vav3 (cyan) immunostaining on cryosections from well-differentiated primary HAECs. Nuclei were stained with DAPI (blue). Dashed lines depict basal side. Scale bar: 20 µm. **c** Colocalization between the red (Cx43) and cyan (Vav3) fluorescent signals was quantified using Pearson's correlation coefficient (3 NCF donors, 3 CF donors, n=12-15, ****p<0.0001). Each donor is represented by a distinct color, and n represents the total number of cryosections analyzed per condition across all donors. Data are shown as mean ± s.e.m.; Two-tailed unpaired Student's t-test. **d** Gene set enrichment analysis (GSEA) of RNA-seq data performed on polarized CTL and CFTR KD Calu-3 cells. Enrichment plots showing two downregulated pathways (highlighted in red) and one upregulated pathway (highlighted in blue). The name of the pathways, the normalized enrichment score (NES) and the false discovery rate (FDR) are indicated at the bottom of each plot.

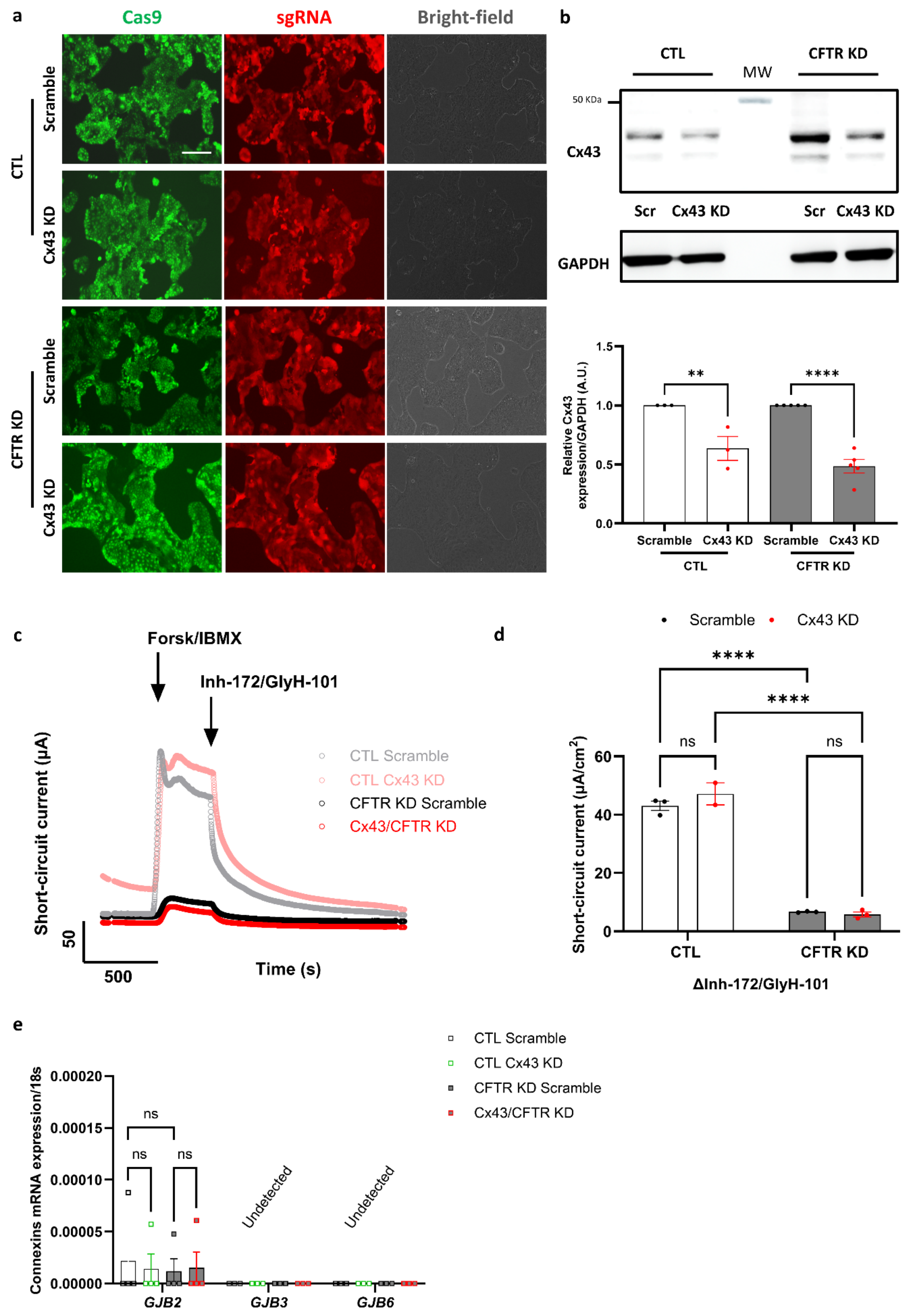
a
Cas9
sgRNA
Bright-field
CTL
Scramble
Cx43 KD
CFTR KD
Scramble
Cx43 KD
b
CTL
MW
CFTR KD
50 KDa
Cx43
Scr
Cx43 KD
Scr
Cx43 KD
GAPDH
Relative Cx43 expression/GAPDH (A.U.)
1.5
1.0
0.5
0.0
**
****
Scramble
Cx43 KD
Scramble
Cx43 KD
CTL
CFTR KD
c
Forsk/IBMX
Inh-172/GlyH-101
CTL Scramble
CTL Cx43 KD
CFTR KD Scramble
Cx43/CFTR KD
Short-circuit current (μA)
50
500
Time (s)
d
Scramble
Cx43 KD
****
****
ns
ns
Short-circuit current (μA/cm²)
60
40
20
0
CTL
CFTR KD
ΔInh-172/GlyH-101
e
CTL Scramble
CTL Cx43 KD
CFTR KD Scramble
Cx43/CFTR KD
Connexins mRNA expression/18s
0.00020
0.00015
0.00010
0.00005
0.00000
ns
ns
ns
Undetected
Undetected
GJB2
GJB3
GJB6

Supplementary Figure 3. Validation of Cx43 depletion by CRISPR-Cas9 gene editing in Calu-3 cells.

a GFP-fused Cas9 (green) and mCherry-co-expressed Scramble or Cx43 sgRNA (red) expression in Calu-3 cells (bright-field). Scale bar: 200 µm. **b** Representative Western blot depicting Cx43 knockdown efficiency in Calu-3 cells. GAPDH was used as a loading control. Molecular weight (MW), Scramble (Scr). The bands were quantified by densitometry (bottom panel), and Cx43 expression was normalized to GAPDH (3-5 biological replicates, **p=0.0061, ***p<0.0001). **c,d** CFTR-dependent ion transport in CFTR KD cells before and after Cx43 depletion. Representatives short-circuit currents (Isc, µA/cm²) are displayed following sequential addition of forskolin (Forsk)/IBMX (apical and basolateral) and Inh-172/GlyH-101 (apical). **d** ΔIsc response to Inh-172/GlyH-101 treatments (2-3 biological replicates, ****p<0.0001). **e** RT-qPCR analysis of *GJB2*, *GJB3*and *GJB6* mRNA expression in polarized CTL and CFTR KD cells depleted or not for Cx43 (3-4 biological replicates). Data are presented as mean ± s.e.m.; One-way ANOVA test (**b**); Two-way ANOVA with post hoc Tukey's multiple comparison test (**d, e**).

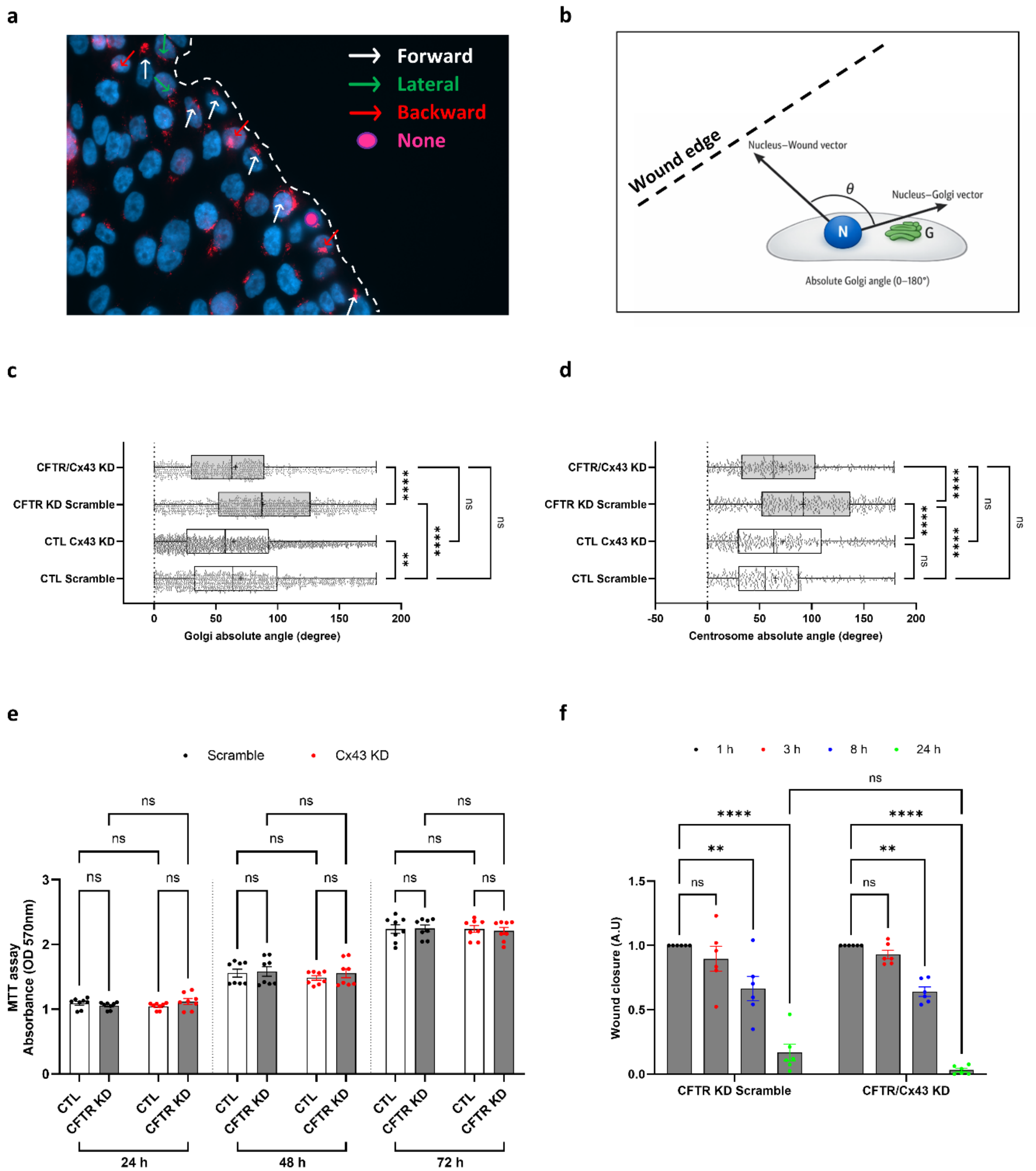

Supplementary Figure 4. Effects of Cx43 depletion on Golgi and centrosome orientation's angles and on proliferation/migration properties of CFTR KD cells.

a Representative image illustrating manual classification of Golgi orientation in leading-edge cells relative to the wound. Cells were annotated based on the position of the Golgi with respect to the nucleus as forward (toward the wound, white arrow), backward (away from the wound, red arrow), lateral (green arrow), or none (pink circle). Only first-row cells at the wound edge were considered. **b** Schematic representation of the automated angle-based quantification. Golgi orientation was defined by the angle between the nucleus-to-Golgi vector and the nucleus-to-wound edge vector. Angles were calculated in the range of 0–180°, where

smaller angles indicate orientation toward the wound edge and larger angles indicate orientation away from it. **c, d** Absolute angles distribution of Golgi (**c**)and centrosome (**d)** orientation relative to the wound edge in leading-edge cells. Absolute angles were calculated between the nucleus-to-organelle vector and the nucleus-to-wound edge vector and expressed in degrees (3 biological replicates, $^{**}p=0.0076$, $^{****}p<0.0001$). **e** Cell growth was assessed by MTT assay in CTL and CFTR KD cells depleted or not for Cx43 at 24, 48 and 72 h post-seeding in 24-well plates (3 biological replicates, $n=8$). *n* represents the total number of wells analyzed per condition. **f** Wound closure was assessed in CFTR KD cells depleted or not for Cx43 using a wound healing assay at 1, 3, 8 and 24 h post-wounding. The percentage of wound closure was quantified relative to the wound area at 1 h (3 biological replicates, $n=6$). *n* represents the total number of analyzed images per condition. Data are represented as box-and-whisker plots (**c, d**), where boxes indicate the interquartile range, the horizontal line represents the median, whiskers show the minimum and maximum values, and the mean is indicated by a "+" symbol. Data in panel **e** and **f** are presented as mean ± s.e.m. Two-way ANOVA with post hoc Tukey's multiple comparison test (**c-f**).

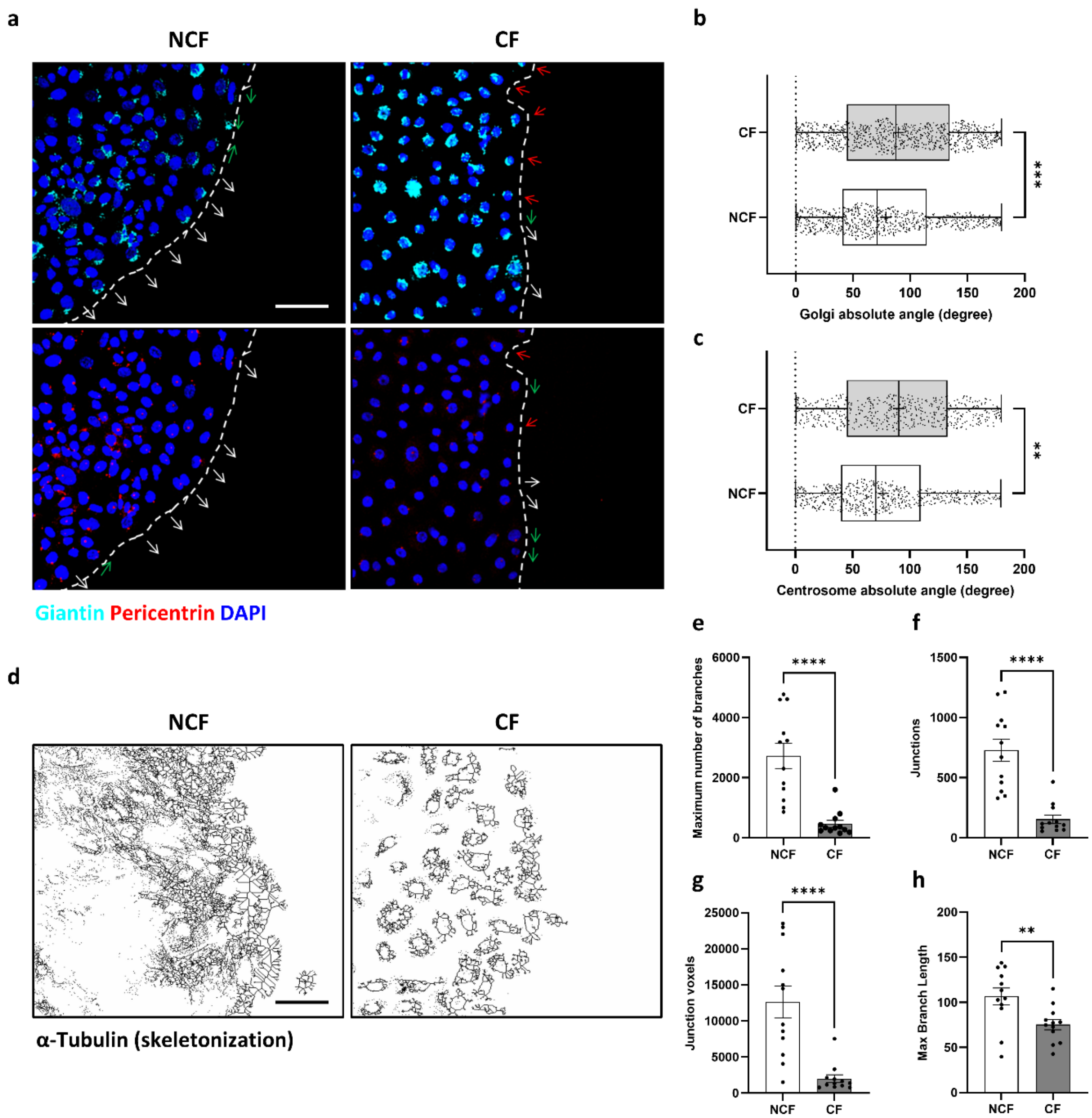

Supplementary Figure 5. Golgi/centrosome orientation and microtubule branching impairment in CF primary HAECs.

a Representative images for giantin (Golgi marker, cyan) and pericentrin (centrosome marker, red) immunostainings performed in repairing NCF and CF primary HAECs grown on coverslips. The orientation of the Golgi and centrosome in leading edge cells was assessed 5 h post-scratch. Nuclei were stained with DAPI (blue). The dashed lines indicate the edge of the wounds. Scale bar: 30 µm. At the leading edge, cells were categorized based on the orientation of the Golgi and centrosome: forward (white arrows), backward (red arrows) or lateral (green arrows). **b,c** Absolute angles distribution of Golgi and centrosome orientation relative to the wound in leading-edge cells. Absolute angles were calculated between the nucleus-to-organelle vector and the nucleus-to-wound edge vector and expressed in absolute degrees. Smaller angles indicate orientation toward the wound edge, whereas larger angles indicate orientation away from the wound (2 biological replicates, $***p=0.0009$, $**p=0.0057$). **d** Representative images showing α-tubulin

immunostaining skeletonization in repairing NCF and CF primary HAECs grown on coverslips. Microtubule networks are shown at 5 h post-wound. Nuclei were stained with DAPI (blue). Scale bar: 30 µm. **e-h** Quantification of the maximum number of branches (**e**, the total count of points in the network where microtubules branch out from an existing structure, 2 biological replicates, n=12, ****p<0.0001), the number of junctions (**f**, the count of points where multiple microtubule segments intersect or converge, 2 biological replicates, n=12, ****p<0.0001), junction voxels (**g**, where multiple microtubule branches meet, 2 biological replicates, n=12, ****p<0.0001), and the maximum branch length (**h,** 2 biological replicates, n=12, **p=0.0097). n represents the total number of analyzed images per condition. Data are represented as box-and-whisker plots, where boxes indicate the interquartile range, the horizontal line represents the median, whiskers show the minimum and maximum values, and the mean is indicated by a "+" symbol (**b,c**) or as mean ± s.e.m. (**e-h**); Two-tailed unpaired Student's t-test (**b,c,e-h**).

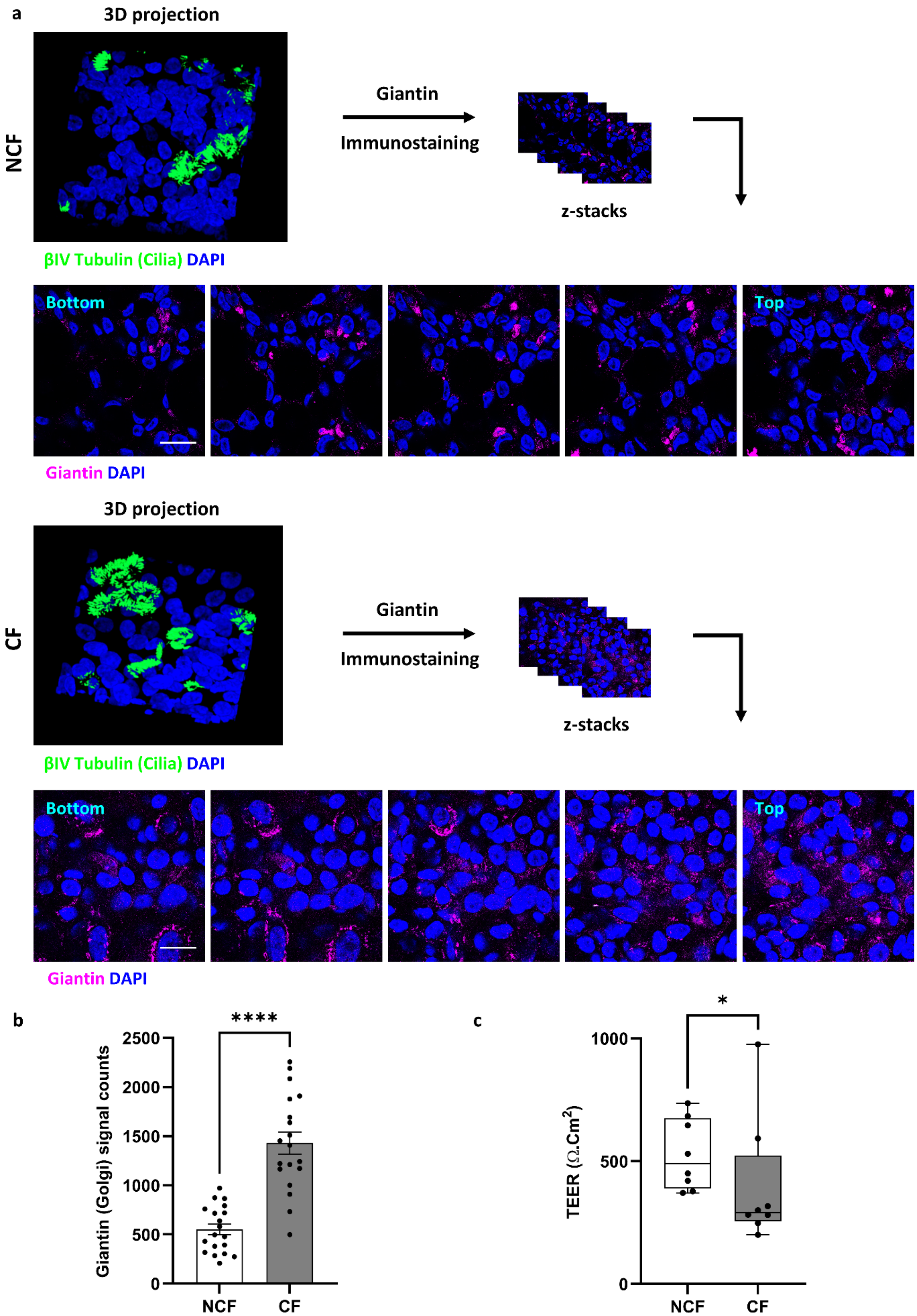
a
3D projection
NCF
Giantin
Immunostaining
z-stacks
βIV Tubulin (Cilia) DAPI
Bottom
Top
Giantin DAPI
3D projection
CF
Giantin
Immunostaining
z-stacks
βIV Tubulin (Cilia) DAPI
Bottom
Top
Giantin DAPI
b
****
Giantin (Golgi) signal counts
2500
2000
1500
1000
500
0
NCF
CF
c
*
TEER (Ω.Cm²)
1000
500
0
NCF
CF

Supplementary Figure 6. Golgi structure dispersal in NCF and CF primary HAECs.

a Representative 3D reconstitution of βIV tubulin (cilia marker, green) confocal images in well-differentiated NCF and CF primary HAECs. For each region of interest, images of giantin (Golgi marker, magenta) immunostaining at multiple single focal planes are shown throughout the epithelium (from the apical (top) to the basal region (bottom)). Nuclei are stained with DAPI (blue). Scale bars: 20 μm. **b** Quantification of Golgi dispersal expressed as number of giantin signal counts (2 biological replicates, n=19, ****p<0.0001) n represents the total number of analyzed images per condition. **c** Transepithelial electrical resistance (TEER) measured in primary HAECs. TEER values are expressed as $\Omega \cdot cm^2$ (8 NCF and 8 CF donors, *p=0.0499). Data are presented as mean ± s.e.m.; Two-tailed unpaired Student's t-test (**b**); Two-tailed Mann–Whitney test (**c**).

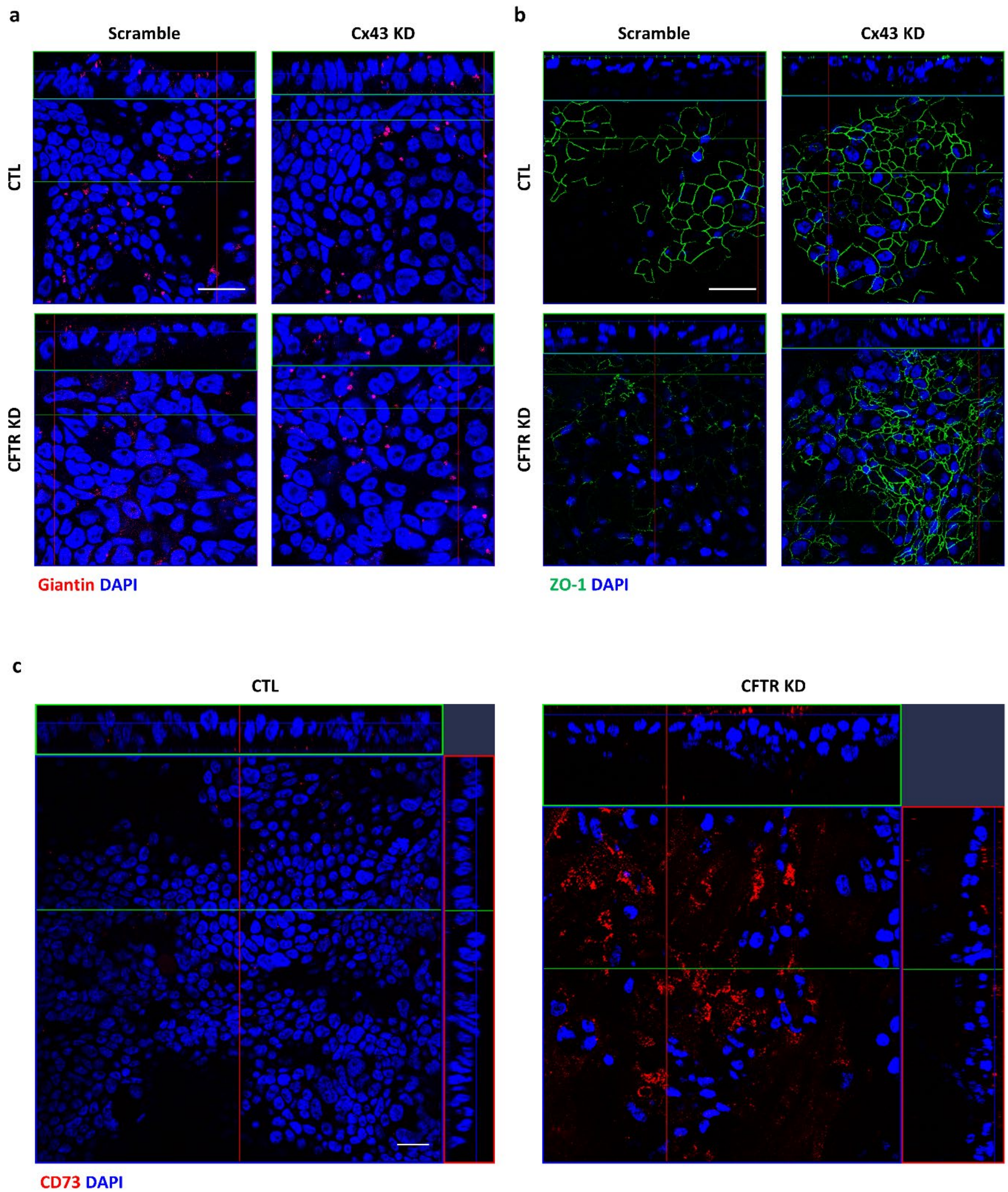

Supplementary Figure 7. Orthogonal projections of giantin, ZO-1 and CD73 z-stack images in polarized Calu-3 cells.

a-c Orthogonal projections of z-stack confocal images corresponding to Figure 5a (**a**), Figure 5d (**b**) and Figure 6b (**c**)**,** respectively showing giantin, ZO-1 and CD73 immunostainings (green) in polarized CTL and CFTR KD at ALI. Nuclei are stained with DAPI (blue). Scale bars: 20 µm.

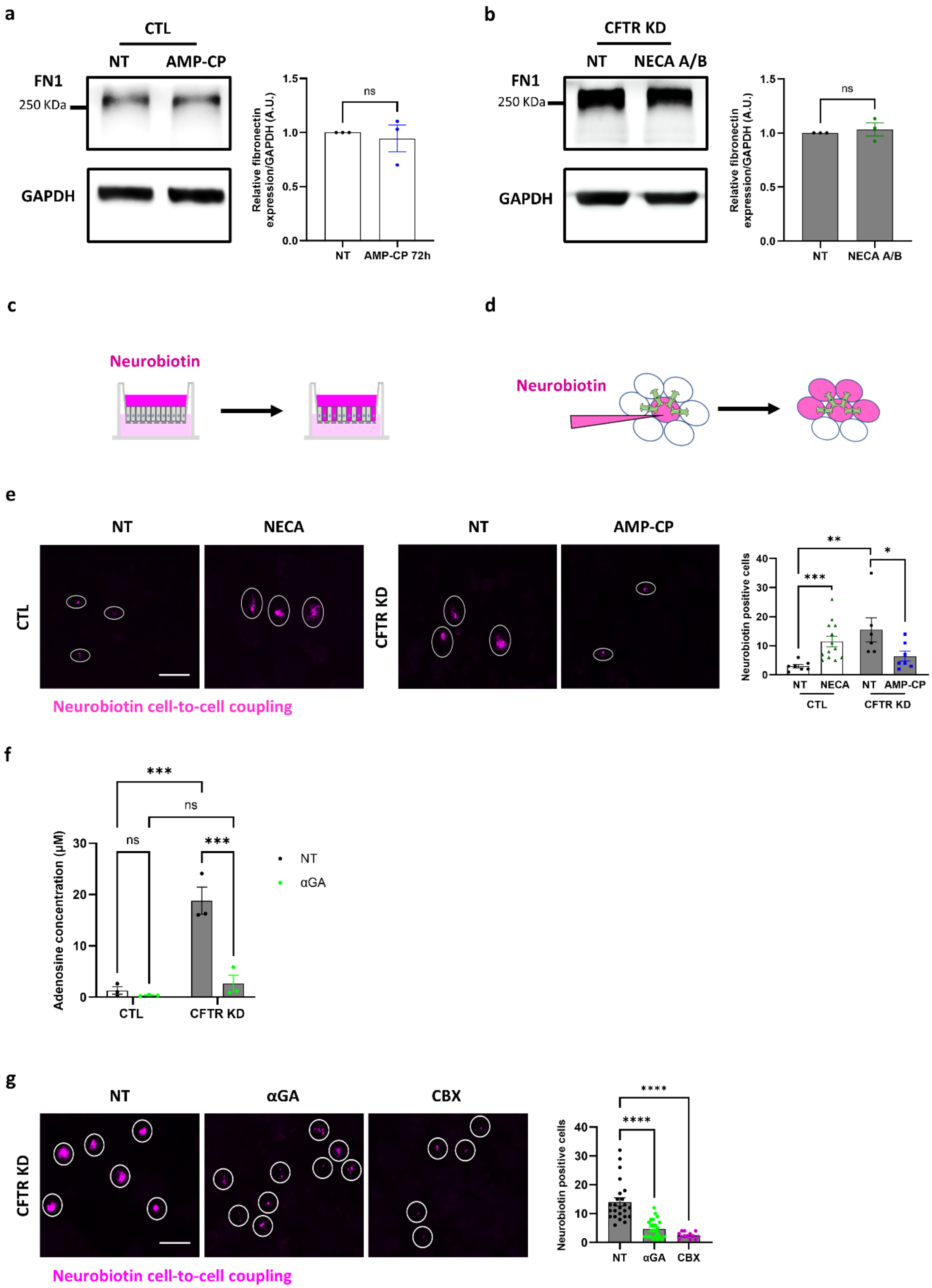
a
CTL
NT
AMP-CP
FN1
250 KDa
GAPDH
Relative fibronectin expression/GAPDH (A.U.)
ns
NT
AMP-CP 72h
b
CFTR KD
NT
NECA A/B
FN1
250 KDa
GAPDH
Relative fibronectin expression/GAPDH (A.U.)
ns
NT
NECA A/B
c
Neurobiotin
d
Neurobiotin
e
NT
NECA
NT
AMP-CP
CTL
CFTR KD
Neurobiotin cell-to-cell coupling
Neurobiotin positive cells
NT
NECA
NT
AMP-CP
CTL
CFTR KD
f
Adenosine concentration (µM)
ns
***
CTL
CFTR KD
NT
αGA
g
NT
αGA
CBX
CFTR KD
Neurobiotin cell-to-cell coupling
Neurobiotin positive cells
****
NT
αGA
CBX

Supplementary Figure 8. Modulation of gap junctional intercellular communication by adenosine signaling and connexin inhibitors in polarized Calu-3 cells.

a, b Representative Western blot depicting fibronectin (FN1) expression in polarized CTL (**a**) and CFTR KD (**b**) Calu-3 cells, respectively treated with AMP-CP or NECA (apically and basally (A/B)) for 72 h. GAPDH was used as a loading control. The bands were quantified by densitometry (right panel of each gel), and FN1 expression was normalized to GAPDH (3 biological replicates). **c** Schematic of the dye uptake protocol to assess hemichannel function in polarized Calu-3 cells**.** 15 min after apical application of neurobiotin at 0.5mg/ml, the cells were rinsed, fixed and neurobiotin uptake was revealed using streptavidin-Alexa Fluor 647 (magenta). **d** Schematic of the dye coupling assay to evaluate gap junctional intercellular communication (GJIC). The diagram illustrates the use of a microelectrode to inject neurobiotin into a single cell. Neurobiotin transfer to adjacent cells (neurobiotin cell-to-cell coupling) was allowed for 3 min to indicate functional GJIC. After multiple microinjections, the cells were fixed and the neurobiotin positive cells revealed using streptavidin-Alexa Fluor 647 (magenta). **e** Representative images showing neurobiotin cell-to-cell coupling in polarized CTL and CFTR KD cells respectively treated with NECA or AMP-CP for 72 h. Each circle indicates neurobiotin transfer to neighboring cells after 3 min post-microinjection in a single cell. Neurobiotin positive cells were labeled with streptavidin-Alexa Fluor 647 (magenta). Scale bar: 100 µm. The right panel shows quantification of neurobiotin-positive cells after each microinjection (3 biological replicates, n=6-17, *p=0.0321, **p= 0.0012, **p=0.0002). **f** Apical adenosine levels were measured in polarized Calu-3 cells following hemichannel opening under calcium-free conditions for 3 h. Both CTL and CFTR KD were pretreated or not with αGA for 30 min prior to calcium-free incubation (3 biological replicates, NT ***p=0.0003, CFTR KD +/- αGA ***p=0.0006). **g** Representative images showing neurobiotin cell-to-cell coupling in polarized CFTR KD cells respectively treated with αGA or CBX for 3 h. Each white circle indicates neurobiotin transfer to neighboring cells after 3 min post-microinjection in a single cell. Neurobiotin positive cells were labeled with streptavidin-Alexa Fluor 647 (magenta). Scale bar: 100 µm. The right panel shows quantification of neurobiotin-positive cells after each microinjection (3 biological replicates, n=13-24, ****p<0.0001). Data are presented as mean ± s.e.m.; Two-tailed unpaired Student's t-test (**a, b**); Two-tailed Mann–Whitney test (**e, f**); Two-way ANOVA with post hoc Tukey's multiple comparison test (**g**).

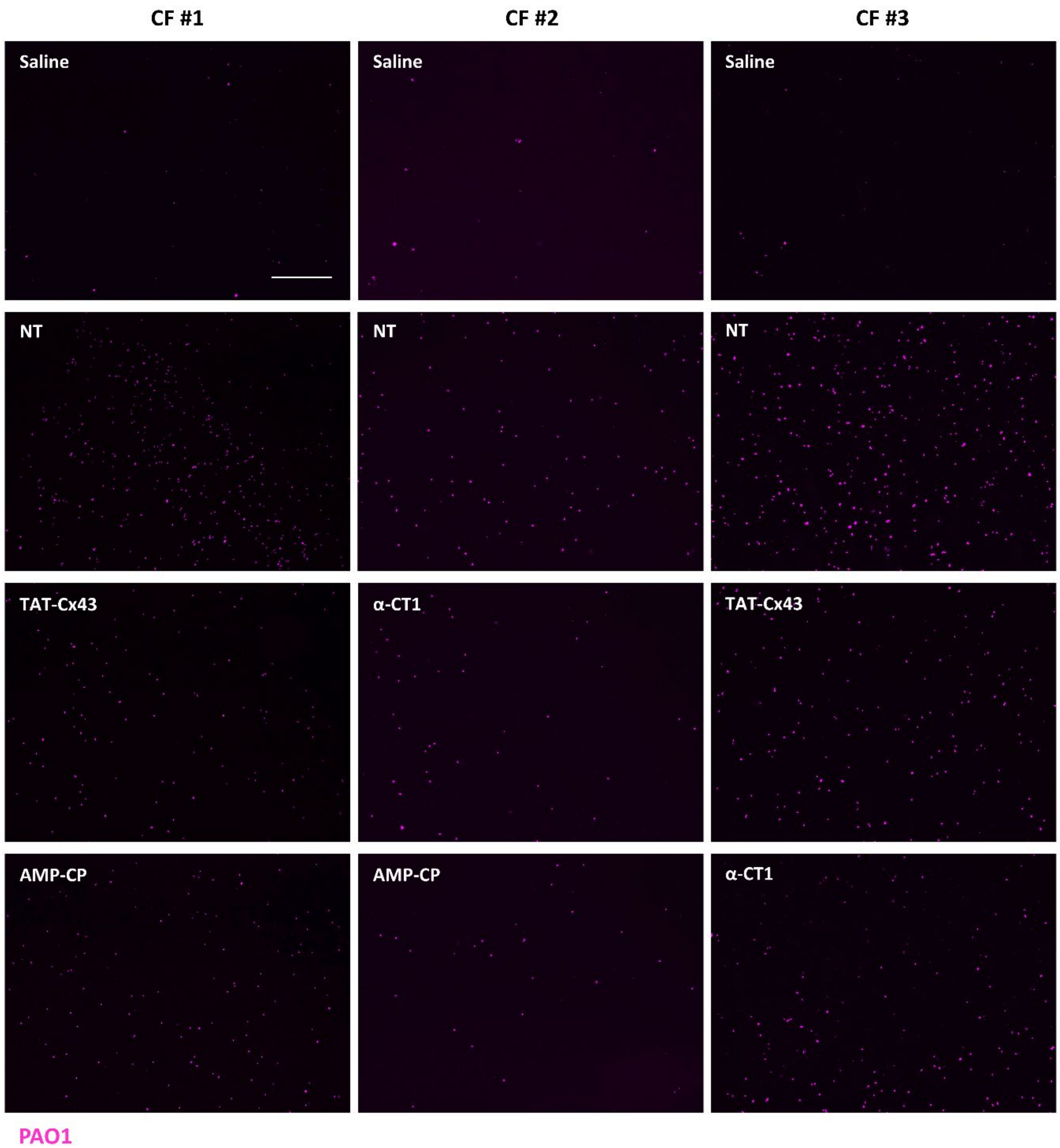

Supplementary Figure 9. PAO1 adhesion to apical secretome from primary HAECs treated with Cx43 mimetic peptides.

Representative images showing adherent PAO1 (magenta) to coverslips coated with apical secretomes collected from CF primary HAECs corresponding to 3 donors. The secretomes were collected from CF primary HAECs basally treated with AMP-CP, TAT-Cx43 or αCT-1 for 72 h. The saline condition represents the adhesion of PAO1 to coverslips coated with control saline. Scale bar: 100 µm.

Supplementary Tables

Supplementary Table 1. clinicopathological characteristics of NCF and CF donors from the transcriptomic datasets shown in Figures 1 and Supplementary figure 1.[1] Transepithelial electrical resistance.

Batch Number	Age	Sex	Smoker	Pathology	Mutation	Origin	TEER[1] (Ω.cm^2)	Cilia beating frequency (Hz)
CF MD0220	29	Female	No	CF	Homozygous F508del	Bronchial	398+/-11	7.0+/-0.2
CF MD0445	25	Female	No	CF	Homozygous F508del	Bronchial	419+/-10	7.4+/-0.2
CF MD0453	35	Male	No	CF	Homozygous F508del	Bronchial	439+/-11	7.5+/-0.1
CF MD0585	-	-	No	CF	Homozygous F508del	Bronchial	-	-
CF MD0592	24	Female	No	CF	Homozygous F508del	Bronchial	227+/-17	8.4+/-0.1
CF MD0485	25	Not known	No	CF	Homozygous F508del	Bronchial	296+/-10	9.2+/-0.1
CF MD0526	16	Female	No	CF	Homozygous F508del	Bronchial	615+/-31	9.0+/-0.5
NCF MD0537	61	Male	No	No Pathology reported	-	Bronchial	431+/-09	7.3+/-0.1
NCF MD0560	66	Male	No	No Pathology reported	-	Bronchial	331+/-12	7.8+/-0.1
NCF MD0201	67	Female	No	No Pathology reported	-	Bronchial	356+/-09	7.1+/-0.2
NCF MD0646	60	Male	No	No Pathology reported	-	Bronchial	208+/-3	8.8+/-0.3

**NCF MD0141**	**60**	**Male**	**No**	**No Pathology reported**	**-**	**Bronchial**	**270+/-16**	**9.3+/-0.2**
**NCF MD0610**	**47**	**Female**	**No**	**No Pathology reported**	**-**	**Bronchial**	**474+/-17**	**10.7+/-0.7**

Supplementary Table 2. Supplier-provided clinicopathological characteristics of NCF and CF donors used in this study.[1] Transepithelial electrical resistance.

Batch Number	Age	Sex	Smoker	Pathology	Mutation	Origin	TEER[1] (Ω.cm^2)	Cilia beating frequency (Hz)
CF MD0637	22	Male	No	CF	Homozygous F508del	Bronchial	515+/-07	6.6+/-0.1
CF MD0567	39	Female	No	CF	Homozygous F508del	Bronchial	887+/-07	7.0+/-0.1
CF MD0220	29	Female	No	CF	Homozygous F508del	Bronchial	378+/-08	8.5+/-0.1
CF MD0679	30	Female	No	CF	Homozygous F508del	Bronchial	496+/-09	9.4+/-0.1
CF MD0673	23	Female	No	CF	Homozygous F508del	Bronchial	412+/-20	7.0+/-0.2
CF MD067302	24	-	No	CF	Homozygous F508del	Bronchial	311+/-22	8.0+/-0.1
CF MD0208	23	Female	No	CF	Homozygous F508del	Bronchial	504+/-17	7.3+/-0.2
CF MD0607	21	Female	No	CF	Homozygous F508del	Bronchial	509+/-09	8.2+/-0.1
NCF MD0782	61	Female	No	No Pathology reported	-	Bronchial	288+/-14	9.1+/-0.2
NCF MD0571	35	Male	No	No Pathology reported	-	Bronchial	521+/-17	8.3+/-0.1
NCF MD0855	72	Male	No	No Pathology reported	-	Bronchial	315+/-08	7.6+/-0.2

**NCF MD0812**	**42**	**Male**	**No**	**No Pathology reported**	**-**	**Bronchial**	**368+/-14**	**9.5+/-0.1**
**NCF MD0786**	**64**	**Male**	**No**	**No Pathology reported**	**-**	**Bronchial**	**333+/-07**	**8.8+/-0.1**
**NCF MD0895**	**21**	**Male**	**No**	**No Pathology reported**	**-**	**Bronchial**	**379+/-13**	**10+/-0.2**
**NCF MD0670**	**15**	**Male**	**No**	**No Pathology reported**	**-**	**Bronchial**	**754+/-31**	**8.1+/-0.2**
**NCF MD0889**	**17**	**Male**	**No**	**No Pathology reported**	**-**	**Bronchial**	**315+/-07**	**6.4+/-0.2**

Supplementary Table 3. Antibodies used in this study.

ANTIBODIES	SOURCE	DILUTIONS	IDENTIFIER
Cx43	BD Transduction Laboratories	1/100	610062
CD73	Cell Signaling	1/500	13160
HuR	Cell Signaling	1/500	12582
Vav3	Sigma-Aldrich	1/100	07-464
Pseudomonas aeruginosa	Abcam	1/100	68538
GAPDH	Millipore	1/3000	MAB374
Giantin	Geneva Antibody Facility	1/100	ABCD_AA341
Pericentrin	Abcam	1/1000	28144
α-Tubulin	Geneva Antibody Facility	1/500	ABCD_AA345
Fibronectin	Polyclonal antisera against human plasma fibronectin	1/10000	Clone 1801
Goat anti-Rabbit HRP	Sigma-Aldrich	1/5000	A8275
Goat anti-Mouse HRP	Sigma-Aldrich	1/5000	A5278
Alexa Fluor™ 647 Phalloidin	Thermo Fisher Scientific	1/400	A22287
Alexa Fluor™ 488 goat anti-rabbit (H+L)	Thermo Fisher Scientific	1/1000	A11078
Alexa Fluor™ 568 goat anti-rabbit (H+L)	Thermo Fisher Scientific	1/1000	A11011
Alexa Fluor™ 647 goat anti-rabbit (H+L)	Thermo Fisher Scientific	1/1000	A21245
Alexa Fluor™ 568 goat anti-mouse (H+L)	Thermo Fisher Scientific	1/1000	A11031
Alexa Fluor™ 647 goat anti-mouse (H+L)	Thermo Fisher Scientific	1/1000	A21236

Uncropped blot image shown in Figure 5.

Figure 5g

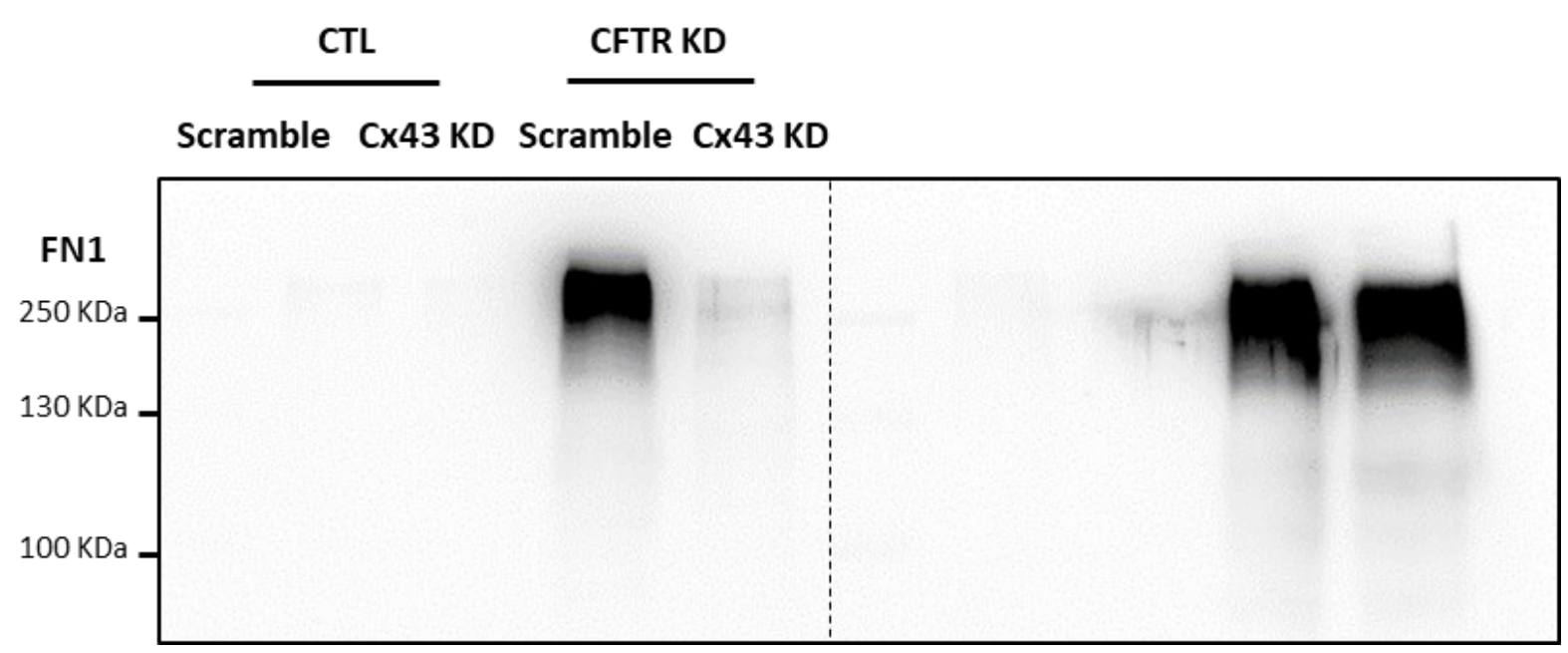

Uncropped blot images shown in Figure 6.

Figure 6a

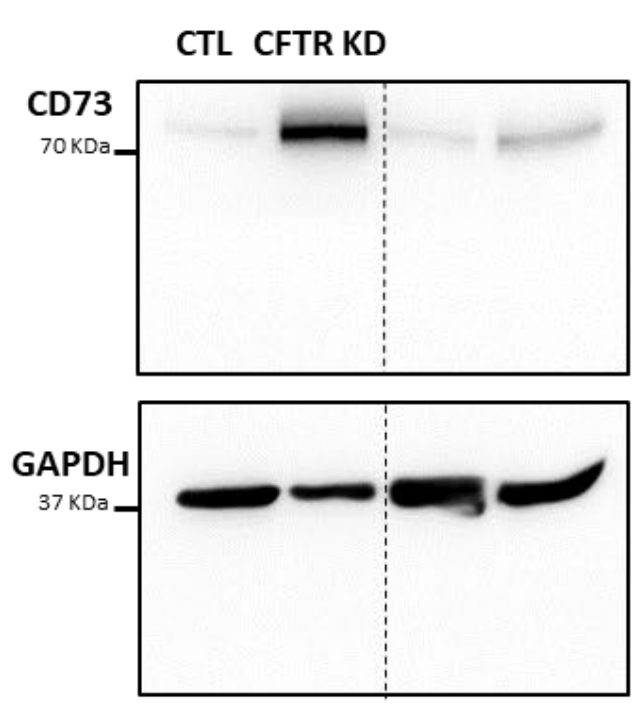

Figure 6c

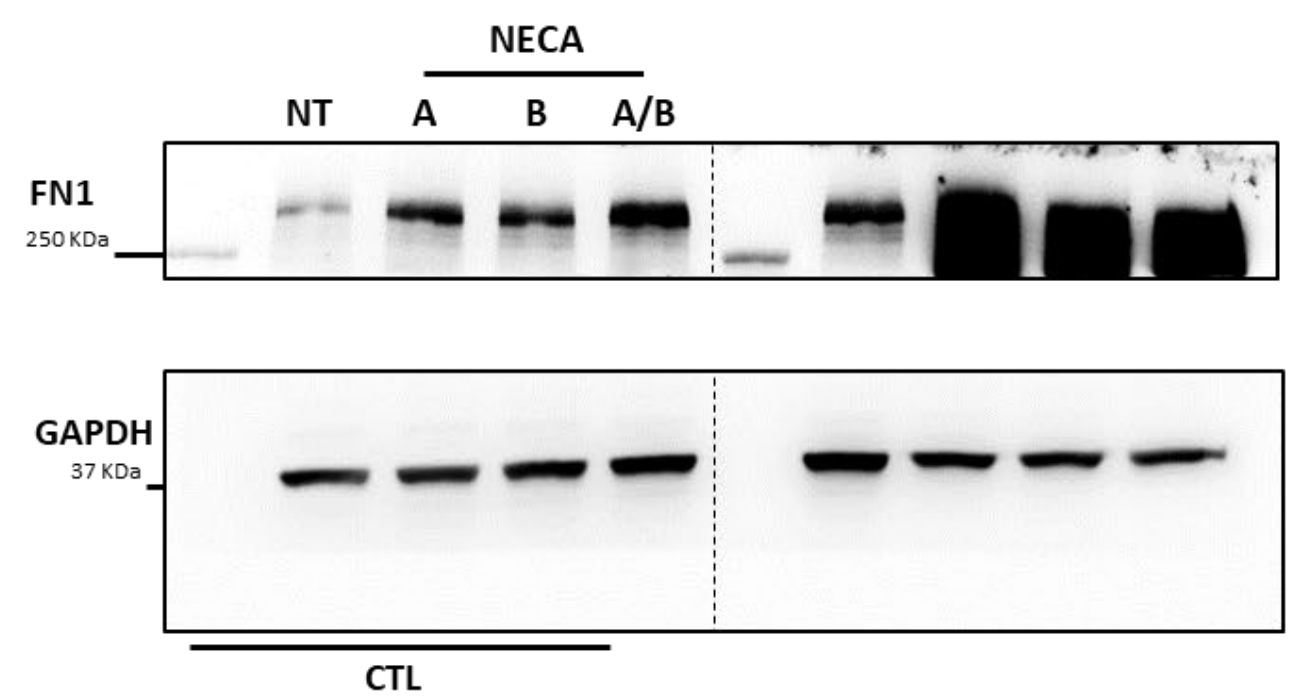

Figure 6d

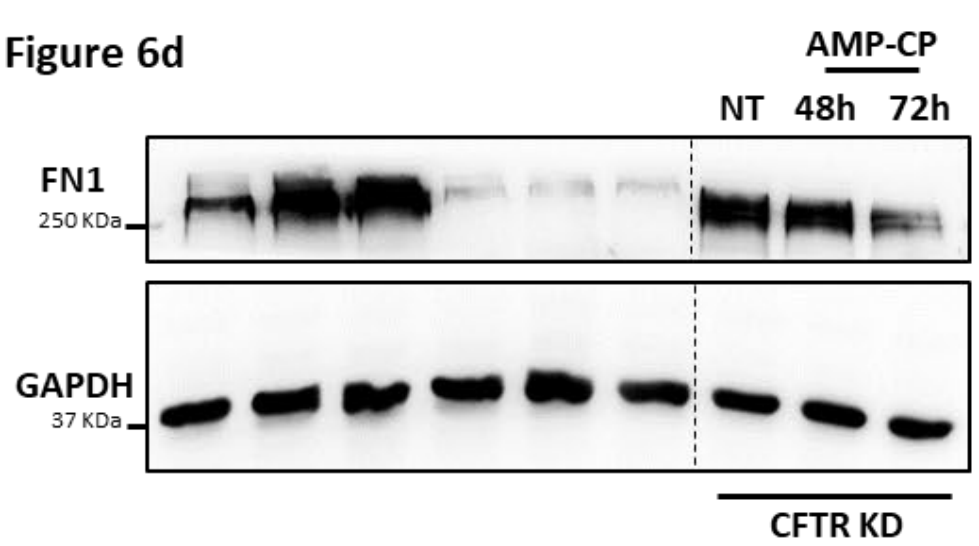

Figure 6e

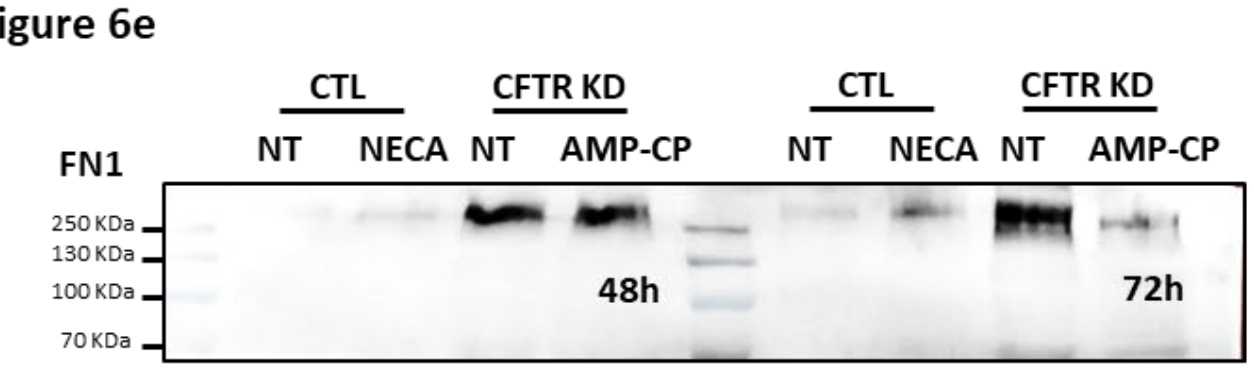

Uncropped blot images shown in Figure 7.

Figure 7c

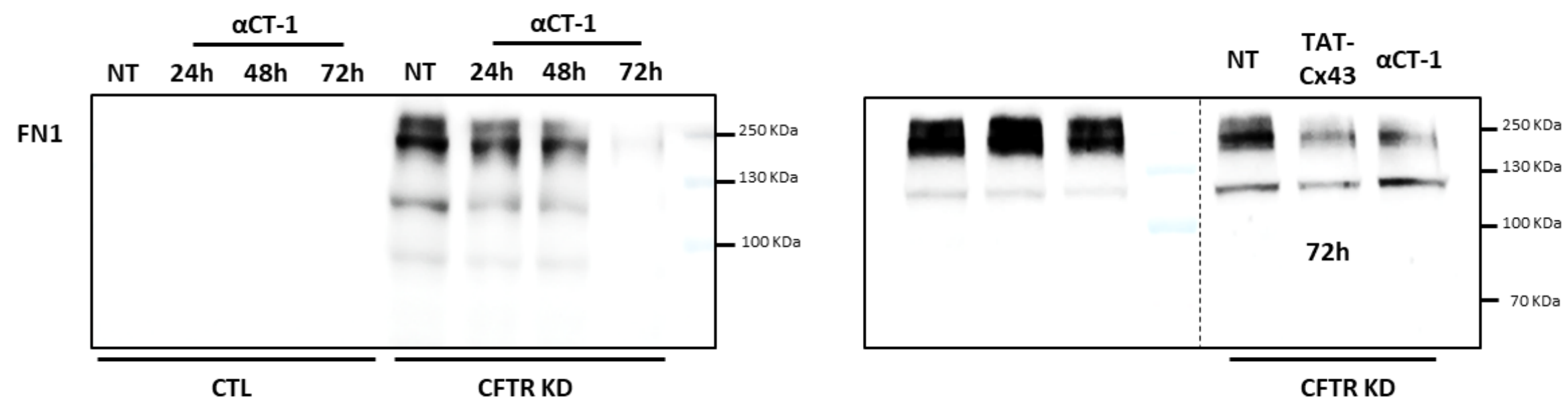

Figure 7d

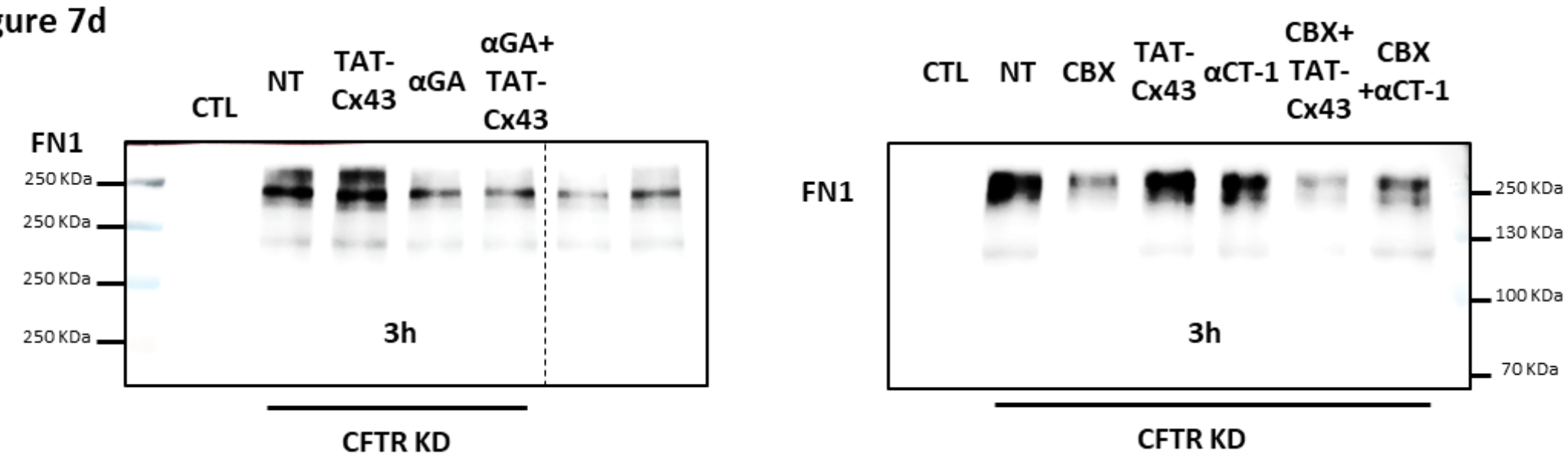

Uncropped blot images shown in Figure 8.

Figure 8c

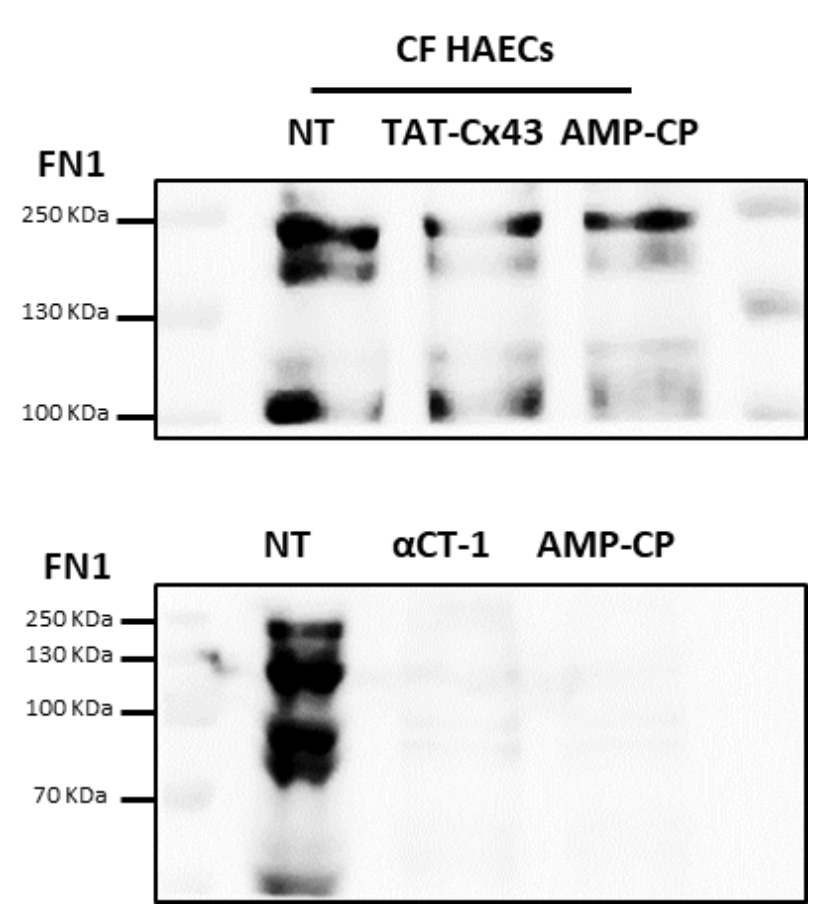

Uncropped blot images shown in Supplementary Figure 3.

Supplementary Figure 3b

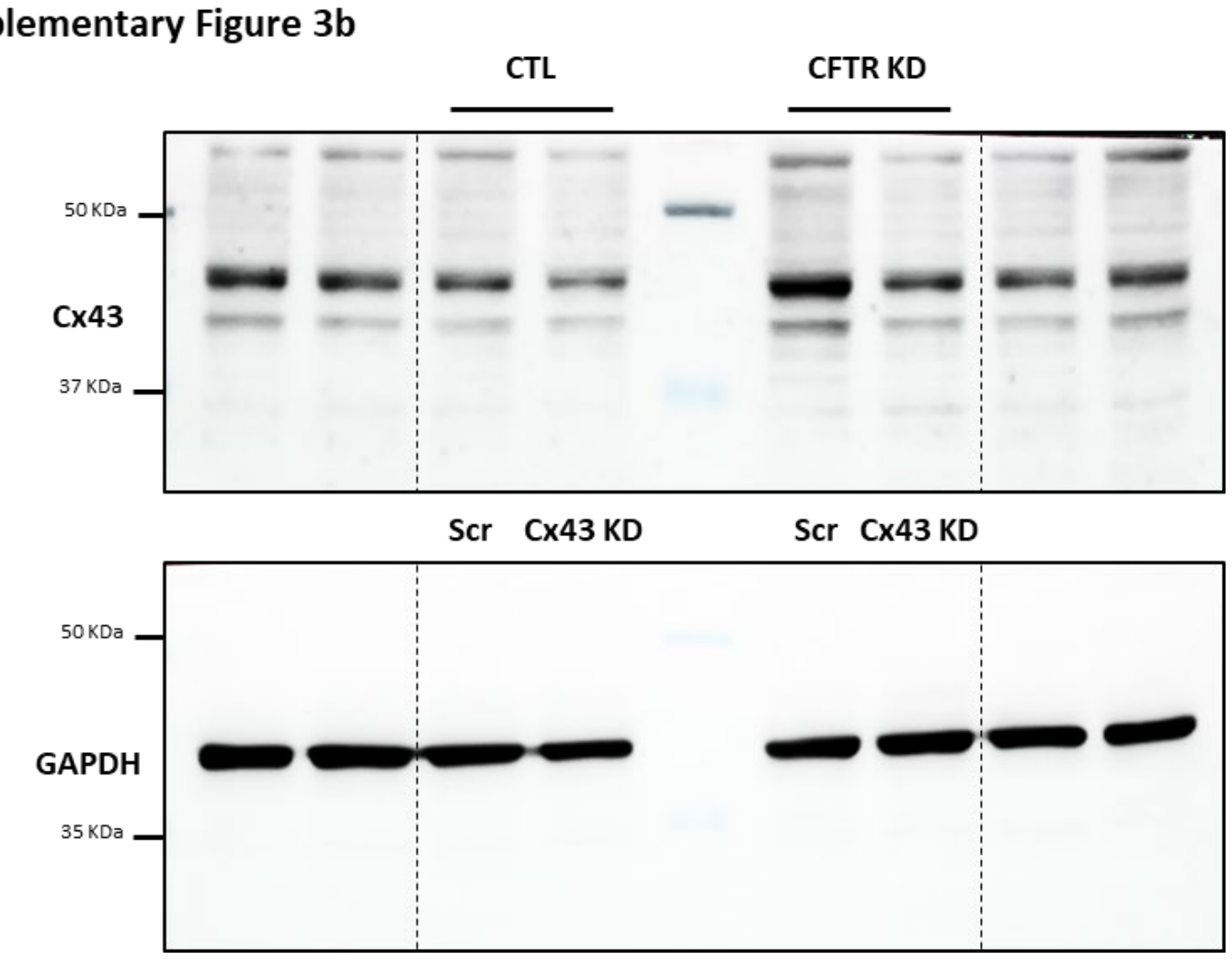

Uncropped blot images shown in Supplementary Figure 8.

Supplementary Figure 8a

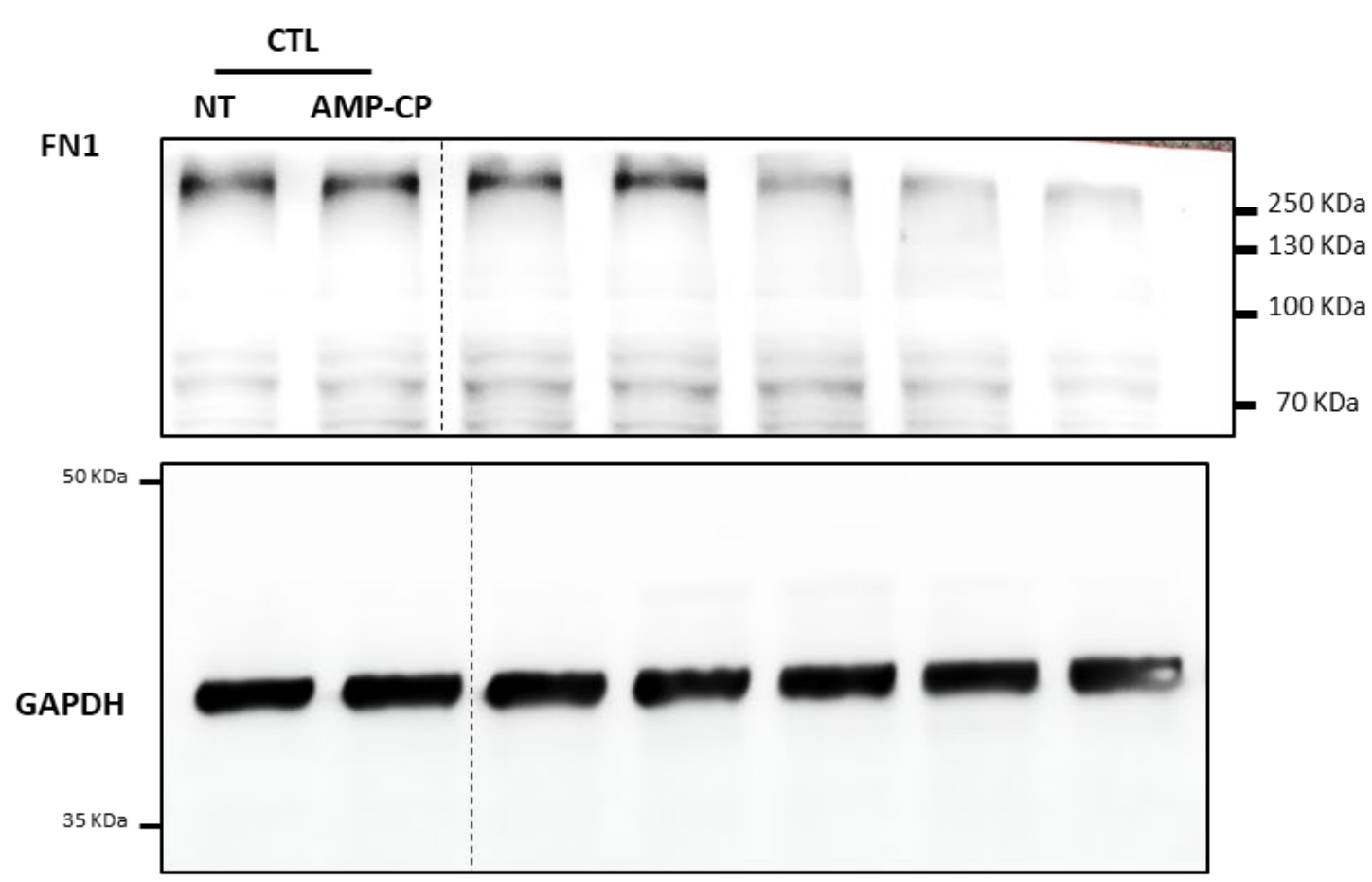

Supplementary Figure 8b

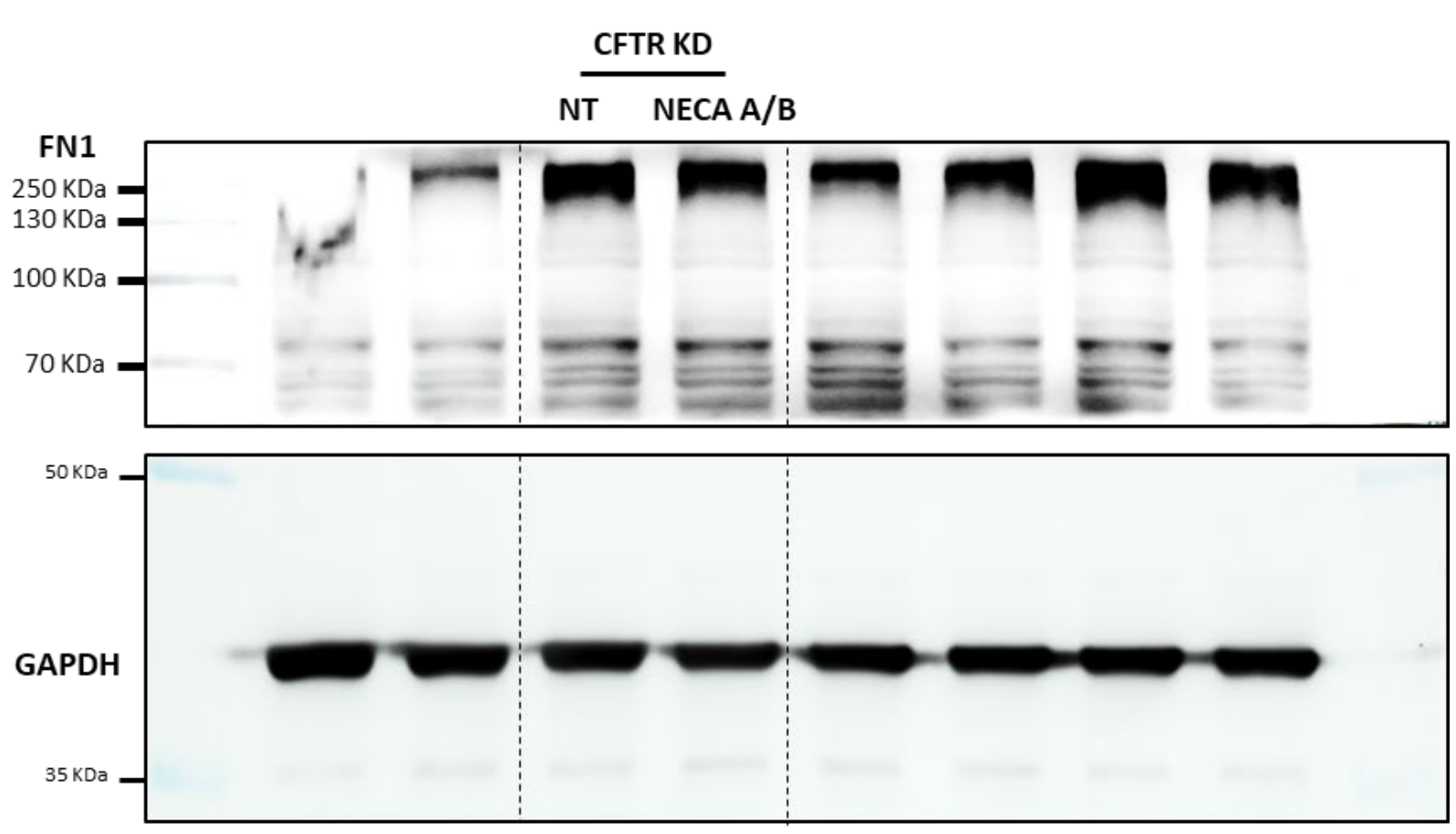

Supplement: Supplementary file 2 [file 42003_2026_10413_MOESM2_ESM.pdf]
